# Supplementary figures and images for: Challenges and opportunities for plastic versus mixed waste enterprises in Greater Accra and Kisumu: A qualitative study
Source: PLoS One. 2026 Jun 8;21(6):e0350670. doi: 10.1371/journal.pone.0350670 (PMC13245779; doi:10.1371/journal.pone.0350670)

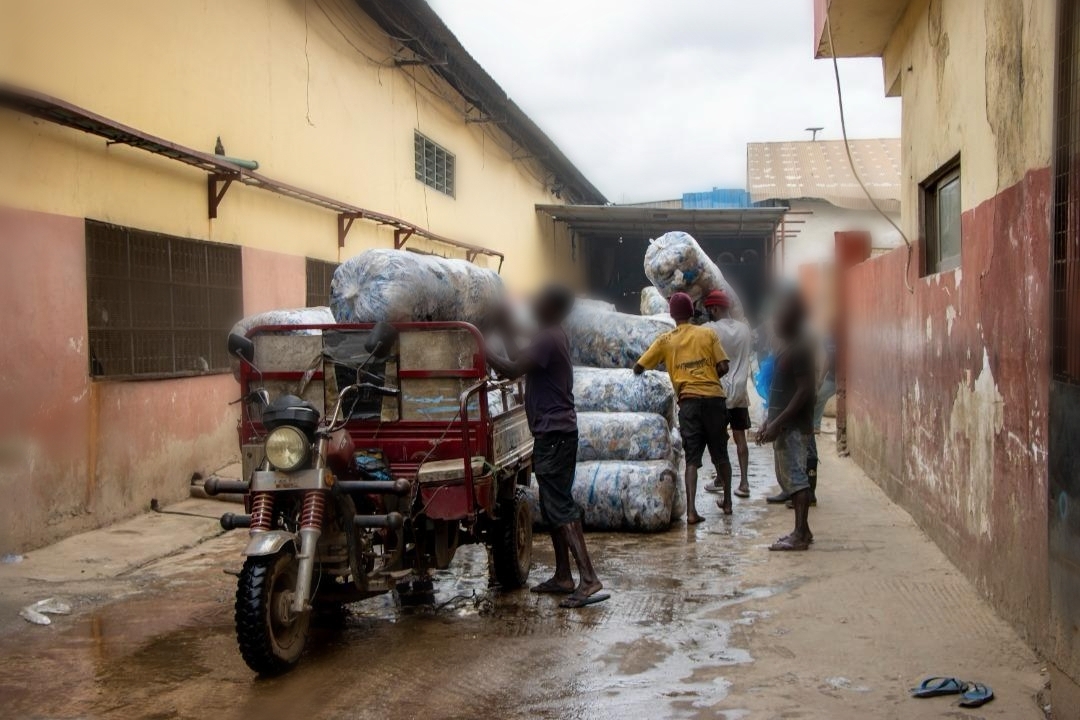

Supplement: S1 File — (JPG) [file pone.0350670.s001.jpg]
