# Supplementary material for: Challenges and opportunities for plastic versus mixed waste enterprises in Greater Accra and Kisumu: A qualitative study
Source: PLoS One. 2026 Jun 8;21(6):e0350670. doi: 10.1371/journal.pone.0350670 (PMC13245779; doi:10.1371/journal.pone.0350670)
Supplement: Attachment — Submitted filename: Comments.pdf [file pone.0350670.s002.pdf]

Reviewers comment

The article examines the "Challenges and opportunities for plastic versus mixed waste enterprises in Greater Accra and Kisumu: a qualitative study," focusing on challenges faced by key waste collection groups. The foundations of the study are enshrined in the Sustainable Development Goals and the AU Agenda 2063. Its context is relevant for Africa, establishing a stronger basis for improving plastic/waste recycling in the region.

The manuscript demonstrates a robust methodological framework, engages in nuanced discussions, and offers valuable insights pertinent to both academic circles and industry professionals, among other stakeholders.

Specific comments:

1. The section on abstract:
   The abstract presents a concise write-up about the topic. The background, objective and results of the study are summarised well. Although this seems to be sufficient, the following comments are suggested.
   - The role of formal-informal SWM collectors towards plastic waste collection is missing. This should be highlighted (in line 19) to establish a linkage and a stronger basis for the study. A more logical order will be: Who handles plastics? What challenges are they facing?
   - How many participants participated in each focus group?
2. The section on introduction:
   The authors have provided an extensive background to the study and supported this with adequate literature. However, the sections look disjointed and verbose. The introduction could be shortened and more direct. It is recommended that the sections are re-ordered to ensure a chronology of the study's background. This chronology could be interspersed or adopted: What is the global SWM (as already established in Lines 39 to 49), who are the actors of SWM, what are their specific roles in SWM, what is their socio-economic motivation, what research gaps remain in their specific role vis-à-vis plastic waste recycling?
3. The section on study sites:
   - How much plastic waste/recyclables is generated in Ghana
   - Line 123,…and processed waste. Introduce "and"
   - Authors may consider including a map of the study areas
   - The authors may consider including information on the number of registered members of the SWM associations, the number of waste management companies, the number of recycling companies, among others.
4. The section on methodology: The methodology discussed is robust and reproducible. This gives credence to the data collected.
5. The section on Results and Discussion:
   The results and discussions are well presented. The following modifications should be considered

- Line 290 to 29 Confirm if the requested amount is GHS30 or 300
- The dollar equivalent should be highlighted in brackets.
- Line 301 to 302 Modify the sentence…but before we you get… (we and you appear together)
- Under Table 5, under Kisumu SWM groups line 2, intermediaries should be formatted in terms of font and font size
- Maintain consistency in font size and type (sentence case, capitalisation) across all tables
- Line 458 to 462 How does this finding compare with other studies in the region or globally
- Authors should examine the influence of security agencies on collectors, particularly regarding the collection of illegal monies, intimidation, and its effects on (de)motivation for waste collectors.
- Line 535 provide a space between references and "chains"

6. The section on Conclusion: The conclusions and recommendations are sufficient.
