# Supplementary material for: Challenges and opportunities for plastic versus mixed waste enterprises in Greater Accra and Kisumu: A qualitative study
Source: PLoS One. 2026 Jun 8;21(6):e0350670. doi: 10.1371/journal.pone.0350670 (PMC13245779; doi:10.1371/journal.pone.0350670)
Supplement: Attachment — Submitted filename: PONE-D-25-23611_R1_Comments.pdf [file pone.0350670.s004.pdf]

PLOS ONE

Challenges and opportunities for plastic versus mixed waste enterprises in Greater Accra and Kisumu: a qualitative study

--Manuscript Draft--

**Manuscript Number:**	PONE-D-25-23611R1
**Article Type:**	Research Article
**Full Title:**	Challenges and opportunities for plastic versus mixed waste enterprises in Greater Accra and Kisumu: a qualitative study
**Short Title:**	Challenges and opportunities for plastic versus mixed waste enterprises
**Corresponding Author:**	Jim Wright<br>University of Southampton<br>Southampton, UNITED KINGDOM OF GREAT BRITAIN AND NORTHERN IRELAND
**Keywords:**	Informality; municipal solid waste management; plastic waste; sub-Saharan Africa; urban studies
**Abstract:**	Consumption of products packaged in plastics is growing in Sub-Saharan African cities, whose populations often lack solid waste management (SWM) services. The informal sector often fills gaps in plastic waste collection and recovery that are not met by formal SWM. To reduce plastic waste mismanagement and its associated health risks, SWM systems are needed that separate plastic waste for reuse or recycling. However, little is known about the contributions and challenges facing the mixed formal-informal enterprises in such cities' SWM chains. This multi-country qualitative study therefore aimed to contrast the specific challenges of plastic waste collectors with those facing general waste collectors. Eleven focus group discussions, 6-12 participants per FGD (n=87 in total), were held with waste collectors in Greater Accra, Ghana, and Kisumu, Kenya, differentiating business types and plastic from mixed waste enterprises. Barriers, enablers, and solutions were identified through thematic coding and analysis of transcripts. We find differing SWM trajectories in Accra versus Kisumu. Domestic waste separation remains low in Kisumu, whilst Accra's SWM system evolved from flexible to rigid plastic separation. Mixed and plastic waste collectors reported shared challenges, such as lack of crushing or transport equipment, harassment, greater hazardous faecal matter in waste streams, and lack of societal recognition. Additionally, specialist plastic waste workers in Greater Accra reported business-specific challenges, notably price volatility from seasonally variable plastic waste generation and from bulk international waste plastic imports, alongside theft of separated plastics. Accra's informal plastic waste sub-collectors were mostly elderly women who reported occupational health challenges from bending and lifting waste. Our findings highlight the need for context-sensitive plastic SWM programmes, including fair bargaining and price regulation measures, occupational health programmes and safer diaper SWM.
**Order of Authors:**	Ruby Hornuvo
	Eunice Emefa Boafor
	Lorna Grace Okotto, PhD
	Joseph Okotto-Okotto
	Mawuli Dzodzomenyo, PhD
	Moses Asamoah, MSc
	Jim Wright
**Response to Reviewers:**	Thank you for the constructive feedback provided on our manuscript. Below are comments made by reviewers and the editorial team, as well as point-by-point responses indicating how and where these have been addressed throughout the manuscript.<br><br>Editorial Comments

Powered by Editorial Manager® and ProduXion Manager® from Aries Systems Corporation

============

COMMENT: Please ensure that your manuscript meets PLOS ONE's style requirements, including those for file naming. The PLOS ONE style templates can be found at…
RESPONSE: The manuscript has been formatted in accordance with PLOS ONE’s manuscript style requirements. Please note that on reviewing PLOS ONE guidance on multi-part figures, we have separated the multipart Figure 1 in our original manuscript into two separate figures, now numbered Figure 2 and 3 (with the introduction of a new additional Figure 1 in response to Reviewer 1's comments).

COMMENT: Please include a complete copy of PLOS’ questionnaire on inclusivity in global research in your revised manuscript. Our policy for research in this area aims to improve transparency in the reporting of research performed outside of researchers’ own country or community. The policy applies to researchers who have travelled to a different country to conduct research, research with Indigenous populations or their lands, and research on cultural artefacts. The questionnaire can also be requested at the journal’s discretion for any other submissions, even if these conditions are not met. Please find more information on the policy and a link to download a blank copy of the questionnaire here: https://journals.plos.org/plosone/s/best-practices-in-research-reporting. Please upload a completed version of your questionnaire as Supporting Information when you resubmit your manuscript.
RESPONSE: We have completed the questionnaire on inclusivity in global research and uploaded it as Supporting Information as requested.

COMMENT: Thank you for stating in your Funding Statement:
[The work was undertaken through the ‘Expanding safe water and waste management service access to off-grid populations in Africa’ project, funded through UKRI Collective award via the Global Challenges Research Fund (ref: ES/T008121/1). The support of the UK Economic and Social Research Council (ESRC) is gratefully acknowledged.].
Please provide an amended statement that declares *all* the funding or sources of support (whether external or internal to your organization) received during this study, as detailed online in our guide for authors at http://journals.plos.org/plosone/s/submit-now. Please also include the statement “There was no additional external funding received for this study.” in your updated Funding Statement.
Please include your amended Funding Statement within your cover letter. We will change the online submission form on your behalf.
RESPONSE: As requested, we provide an updated funding including the explicit statement about no further external funding via our latest covering letter.

COMMENT: Thank you for stating the following in the Competing Interests section:
[MD is academic representative on Ghana’s National Technical Working Group on Sanitation, which informs solid waste management policy. The other authors declare no competing interests.].
Please confirm that this does not alter your adherence to all PLOS ONE policies on sharing data and materials, by including the following statement: "This does not alter our adherence to PLOS ONE policies on sharing data and materials.” (as detailed online in our guide for authors http://journals.plos.org/plosone/s/competing-interests).
If there are restrictions on sharing of data and/or materials, please state these. Please note that we cannot proceed with consideration of your article until this information has been declared.
Please include your updated Competing Interests statement in your cover letter; we will change the online submission form on your behalf.
RESPONSE: The updated competing interest has been included in the cover letter.

COMMENT: We note that your paper includes detailed descriptions of individual participants. As per the PLOS ONE policy (http://journals.plos.org/plosone/s/submission-guidelines#loc-human-subjects-research) on papers that include identifying, or potentially identifying, information, the individual(s) or parent(s)/guardian(s) must be informed of the terms of the PLOS open-access (CC-BY) license and provide specific permission for publication of these details under the terms of this license. Please download the Consent Form for Publication in a PLOS Journal (http://journals.plos.org/plosone/s/file?id=8ce6/plos-consent-form-english.pdf). The signed consent form should not be submitted with the manuscript, but

should be securely filed in the individual's case notes. Please amend the methods section and ethics statement of the manuscript to explicitly state that the patient/participant has provided consent for publication: “The individual in this manuscript has given written informed consent (as outlined in PLOS consent form) to publish these case details”.
RESPONSE: On reviewing our manuscript, we believe that this comment refers particularly to the supplementary materials (S1 Table) of our original submission, as per the comment below. This table actually described the demographic and educational characteristics of the Kenyan research team members, NOT OUR PARTICIPANTS. We included this table to provide detail on the backgrounds and nature of those conducting this qualitative research study, thereby meeting items on the COREQ checklist for reporting qualitative research (https://www.equator-network.org/reporting-guidelines/coreq/). Nonetheless, we accept that there is a data protection issue around reporting the personal characteristics of Kenyan project team members, so we have now deleted it without compromising COREQ compliance. We have also reviewed all quotations in the main manuscript, further redacting any personal details (e.g. of specific sites or occupations) that could potentially lead to inadvertent disclosure of participant identities. If we have misunderstood the editorial concerns, please let us know.

COMMENT: We note that there is identifying data in the Supporting Information file <S1 Table>. Due to the inclusion of these potentially identifying data, we have removed this file from your file inventory. Prior to sharing human research participant data, authors should consult with an ethics committee to ensure data are shared in accordance with participant consent and all applicable local laws.
Data sharing should never compromise participant privacy. It is therefore not appropriate to publicly share personally identifiable data on human research participants. The following are examples of data that should not be shared:
-Name, initials, physical address
-Ages more specific than whole numbers
-Internet protocol (IP) address
-Specific dates (birth dates, death dates, examination dates, etc.)
-Contact information such as phone number or email address
-Location data
-ID numbers that seem specific (long numbers, include initials, titled “Hospital ID”) rather than random (small numbers in numerical order
Data that are not directly identifying may also be inappropriate to share, as in combination they can become identifying. For example, data collected from a small group of participants, vulnerable populations, or private groups should not be shared if they involve indirect identifiers (such as sex, ethnicity, location, etc.) that may risk the identification of study participants.
Additional guidance on preparing raw data for publication can be found in our Data Policy (https://journals.plos.org/plosone/s/data-availability#loc-human-research-participant-data-and-other-sensitive-data) and in the following article: http://www.bmj.com/content/340/bmj.c181.long.
Please remove or anonymize all personal information (<specific identifying information in file to be removed>), ensure that the data shared are in accordance with participant consent, and re-upload a fully anonymized data set. Please note that spreadsheet columns with personal information must be removed and not hidden as all hidden columns will appear in the published file.
RESPONSE: As noted above, supplementary file S1 Table, which actually described the characteristics of Kenyan project research team members but NOT RESEARCH PARTICIPANTS, has now been deleted as supporting information.

Reviewer #1
========

1.Abstract:
•The role of formal-informal SWM collectors towards plastic waste collection is missing. This should be highlighted (in line 19) to establish a linkage and a stronger basis for the study. A more logical order will be: Who handles plastics? What challenges are they facing?
RESPONSE: This has been addressed in line 19 of the Abstract section (Page 2)
•How many participants participated in each focus group?

RESPONSE: This has been included in the methods section of the abstract and can be found in lines 26 and 27 (i.e. 6-12 participants per FGD (n=87))
2.Introduction
•The authors have provided an extensive background to the study and supported this with adequate literature. However, the sections look disjointed and verbose. The introduction could be shortened and more direct. It is recommended that the sections are re-ordered to ensure a chronology of the study's background. This chronology could be interspersed or adopted: What is the global SWM (as already established in Lines 39 to 49), who are the actors of SWM, what are their specific roles in SWM, what is their socio-economic motivation, what research gaps remain in their specific role vis-à-vis plastic waste recycling?
RESPONSE: The introduction has been re-ordered for a better flow, with some content abridged to create a more concise background to the study.
3.Materials and Methods
•study sites
How much plastic waste/recyclables is generated in Ghana?
RESPONSE: This information has now been included under the study sites (page 4).
•Line 123,…and processed waste. Introduce "and"
RESPONSE: This has been introduced (line 128, page 5)
•Authors may consider including a map of the study areas
RESPONSE: We have inserted a new Figure 1 in our Materials and Methods section, showing maps of the two study areas, drawing on the CC BY license of geoboundaries.
•The authors may consider including information on the number of registered members of the SWM associations, the number of waste management companies, the number of recycling companies, among others.
RESPONSE: In our revised manuscript, we now include some additional statistics on numbers of recycling companies and example membership figures for a SWM association.
5.Results and Discussion
•Line 290 to 29 Confirm if the requested amount is GHS30 or 300
RESPONSE: The requested amount is indeed 300 cedis.
•The dollar equivalent should be highlighted in brackets.
RESPONSE: The dollar equivalent has been added (page 14) and for two other quotations, we have similarly added dollar equivalent values for KSh amounts.
•Line 301 to 302 Modify the sentence…but before we you get… (we and you appear together)
RESPONSE: This has been modified under the results section (Pages 14-15)
•Under Table 5, under Kisumu SWM groups line 2, intermediaries should be formatted in terms of font and font sizeRESPONSE: This table has been formatted accordingly Maintain consistency in font size and type (sentence case, capitalisation) across all tables
RESPONSE: All tables have been formatted to have consistency in font size and type as well as capitalization.
•Line 458 to 462 How does this finding compare with other studies in the region or globally
RESPONSE: We have edited the text to address this comment.
RESPONSE: We now note that other studies have reported theft of personal positions (in insecure neighbourhoods) and of waste collection equipment, but not the specific theft of valuable separated plastic waste.
•Authors should examine the influence of security agencies on collectors, particularly regarding the collection of illegal monies, intimidation, and its effects on (de)motivation for waste collectors.
RESPONSE: We now return to the theme of harassment by authorities in paragraph 4 of our revised Discussion, noting that this is a recurrent theme in literature on informal waste collectors.
•Line 535 provide a space between references and "chains"
RESPONSE: This change has been effected (page 25)

Reviewer # 2
========

6.First, I recommend using consistent precision across all tables. Currently, some

	columns have precision to one digit while others do not.<br>RESPONSE: All tables have now been formatted accordingly, with all percentages given to 1 decimal place.<br><br>7.please double-check the initials from lines 182 to 187. I am not sure if these are the initials of the authors or not. If they are, please ensure consistency.<br>RESPONSE: We have amended the text here to make it clear that author initials are listed at this point, checking formatting of initials for consistency.<br>8.Lastly, Table 5 has a different font style compared to all the other tables. Please standardize the font style for uniformity.<br>RESPONSE: Table 5 has been formatted to have consistent font size and style with all the other tables.
**Additional Information:**	
**Question**	**Response**
**Financial Disclosure**<br><br>Enter a financial disclosure statement that describes the sources of funding for the work included in this submission. Review the submission guidelines for detailed requirements. View published research articles from *PLOS ONE* for specific examples.<br><br>This statement is required for submission and **will appear in the published article** if the submission is accepted. Please make sure it is accurate.<br><br>**Funded studies**<br>Enter a statement with the following details:<br>• Initials of the authors who received each award<br>• Grant numbers awarded to each author<br>• The full name of each funder<br>• URL of each funder website<br>• Did the sponsors or funders play any role in the study design, data collection and analysis, decision to publish, or preparation of the manuscript?<br><br>Did you receive funding for this work?	Yes
Please add funding details.<br>as follow-up to "**Financial Disclosure**<br><br>Enter a financial disclosure statement that describes the sources of funding for the work included in this submission. Review the submission guidelines for detailed requirements. View published research	The work was undertaken through the ‘Expanding safe water and waste management service access to off-grid populations in Africa’ project, funded through UKRI Collective award via the Global Challenges Research Fund (ref: ES/T008121/1). The support of the UK Economic and Social Research Council (ESRC) is gratefully acknowledged.

Powered by Editorial Manager® and ProduXion Manager® from Aries Systems Corporation

articles from *PLOS ONE* for specific examples.<br><br>This statement is required for submission and **will appear in the published article** if the submission is accepted. Please make sure it is accurate.<br><br>**Funded studies**<br>Enter a statement with the following details:<br>• Initials of the authors who received each award<br>• Grant numbers awarded to each author<br>• The full name of each funder<br>• URL of each funder website<br>• Did the sponsors or funders play any role in the study design, data collection and analysis, decision to publish, or preparation of the manuscript?<br><br>Did you receive funding for this work?"	
Please select the country of your main research funder (please select carefully as in some cases this is used in fee calculation).<br>as follow-up to "**Financial Disclosure**<br><br>Enter a financial disclosure statement that describes the sources of funding for the work included in this submission. Review the submission guidelines for detailed requirements. View published research articles from *PLOS ONE* for specific examples.<br><br>This statement is required for submission and **will appear in the published article** if the submission is accepted. Please make sure it is accurate.	UNITED KINGDOM - GB

**Funded studies**<br>Enter a statement with the following details:<br>• Initials of the authors who received each award<br>• Grant numbers awarded to each author<br>• The full name of each funder<br>• URL of each funder website<br>• Did the sponsors or funders play any role in the study design, data collection and analysis, decision to publish, or preparation of the manuscript?<br><br>Did you receive funding for this work?"	
**Competing Interests**<br><br>Use the instructions below to enter a competing interest statement for this submission. On behalf of all authors, disclose any competing interests that could be perceived to bias this work—acknowledging all financial support and any other relevant financial or non-financial competing interests.<br><br>This statement is required for submission and **will appear in the published article** if the submission is accepted. Please make sure it is accurate and that any funding sources listed in your Funding Information later in the submission form are also declared in your Financial Disclosure statement.<br><br>View published research articles from *PLOS ONE* for specific examples.	MD is academic representative on Ghana's National Technical Working Group on Sanitation, which informs solid waste management policy. The other authors declare no competing interests.

Powered by Editorial Manager® and ProduXion Manager® from Aries Systems Corporation

**NO authors have competing interests**<br><br>Enter: *The authors have declared that no competing interests exist.*<br><br>**Authors with competing interests**<br><br>Enter competing interest details beginning with this statement:<br><br>*I have read the journal's policy and the authors of this manuscript have the following competing interests: [insert competing interests here]*<br><br>* typeset	
**Ethics Statement**<br><br>Enter an ethics statement for this submission. This statement is required if the study involved:<br><br>• Human participants<br>• Human specimens or tissue<br>• Vertebrate animals or cephalopods<br>• Vertebrate embryos or tissues<br>• Field research<br><br>Write "N/A" if the submission does not require an ethics statement.<br><br>General guidance is provided below. Consult the submission guidelines for detailed instructions. **Make sure that all information entered here is included in the Methods section of the manuscript.**	The study was approved by the Faculty of Environmental and Life Sciences Ethical Review Committee, University of Southampton, UK (reference: 55755; approval date 26th August 2020), the Jaramogi Odinga Oginga University of Science and Technology Ethical Review Committee (reference: ERC/23/6/20-4, approval date: 17th August 2020) and the Institutional Review Board of the Noguchi Memorial Institute for Medical Research, University of Ghana (Ref: 003/20-21; approval date: 2nd September 2020). Written informed consent was obtained from participants.

Powered by Editorial Manager® and ProduXion Manager® from Aries Systems Corporation

**Format for specific study types**<br><br>**Human Subject Research (involving human participants and/or tissue)**<br>• Give the name of the institutional review board or ethics committee that approved the study<br>• Include the approval number and/or a statement indicating approval of this research<br>• Indicate the form of consent obtained (written/oral) or the reason that consent was not obtained (e.g. the data were analyzed anonymously)<br><br>**Animal Research (involving vertebrate animals, embryos or tissues)**<br>• Provide the name of the Institutional Animal Care and Use Committee (IACUC) or other relevant ethics board that reviewed the study protocol, and indicate whether they approved this research or granted a formal waiver of ethical approval<br>• Include an approval number if one was obtained<br>• If the study involved *non-human primates*, add *additional details* about animal welfare and steps taken to ameliorate suffering<br>• If anesthesia, euthanasia, or any kind of animal sacrifice is part of the study, include briefly which substances and/or methods were applied<br><br>**Field Research**<br><br>Include the following details if this study involves the collection of plant, animal, or other materials from a natural setting:<br>• Field permit number<br>• Name of the institution or relevant body that granted permission	
**Data Availability**<br><br>Authors are required to make all data underlying the findings described fully available, without restriction, and from the time of publication. PLOS allows rare exceptions to address legal and ethical concerns. See the PLOS Data Policy and FAQ for detailed information.	Yes - all data are fully available without restriction

<table>
<tr>
<td>A Data Availability Statement describing where the data can be found is required at submission. Your answers to this question constitute the Data Availability Statement and will be published in the article, if accepted.

Important: Stating ‘data available on request from the author’ is not sufficient. If your data are only available upon request, select ‘No’ for the first question and explain your exceptional situation in the text box.

Do the authors confirm that all data underlying the findings described in their manuscript are fully available without restriction?</td>
<td></td>
</tr>
<tr>
<td>Describe where the data may be found in full sentences. If you are copying our sample text, replace any instances of XXX with the appropriate details.

• If the data are held or will be held in a public repository, include URLs, accession numbers or DOIs. If this information will only be available after acceptance, indicate this by ticking the box below. For example: All XXX files are available from the XXX database (accession number(s) XXX, XXX.).
• If the data are all contained within the manuscript and/or Supporting Information files, enter the following: All relevant data are within the manuscript and its Supporting Information files.
• If neither of these applies but you are able to provide details of access elsewhere, with or without limitations, please do so. For example:

Data cannot be shared publicly because of [XXX]. Data are available from the XXX Institutional Data Access / Ethics Committee (contact via XXX) for researchers who meet the criteria for access to confidential data.

The data underlying the results presented in the study are available from (include the name of the third party</td>
<td>Anonymised transcripts of focus group discussions from this study are available for research purposes through the UK Data Service:

Omom, C. Okotto-Okotto, J. Wright, J. Okotto, L. (2024). Experiences and Challenges of Plastic Waste Collectors in Kenya; A Qualitative Study Among Informal Waste Collectors in Kisumu City, Kenya, 2023. [Data Collection]. Colchester, Essex: UK Data Service. 10.5255/UKDA-SN-856990

Hornuvo, R., Boafor E., Asamoah, M., Amoah, J., Myers-Hansen, G., Amponsah, M., Wright, J. Dzodzomenyo, M., Umar, F. (2023). Increasing Recycling of Water Sachet Plastic Among Ghana’s Off-Grid Urban Populations: A Qualitative Study Among Informal Waste Collectors in Greater Accra, Ghana, 2022. [Data Collection]. Colchester, Essex: UK Data Service. 10.5255/UKDA-SN-856766</td>
</tr>
</table>

and contact information or URL).<br>• This text is appropriate if the data are owned by a third party and authors do not have permission to share the data.<br><br>* typeset	
Additional data availability information:	

Powered by Editorial Manager® and ProduXion Manager® from Aries Systems Corporation

Manuscript

Click here to access/download;Manuscript;Hornuvo_etal_v12_August

Challenges and opportunities for plastic versus mixed waste enterprises in Greater Accra and Kisumu: A qualitative study

Ruby Hornuvo[1,#a], Eunice Emefa Boafor[1], Lorna-Grace Okotto[2], Joseph Okotto-Okotto[3], Mawuli Dzodzomenyo[1], Moses Asamoah[1, 4], Jim Wright[4*]

[1] School of Public Health, University of Ghana, Legon, Accra, Ghana.

[2] School of Spatial Planning and Natural Resource Management, Jaramogi Oginga Odinga University of Science and Technology, Bondo, Kenya.

[3]Victoria Institute for Research on Environment and Development International, Rabuor, Kenya.

[4]Social Statistics and Demography, University of Southampton, Southampton, UK.

[5] School of Geography and Environmental Science, University of Southampton, Southampton, UK.

[#a]Current address: School of Public Health and Health Sciences, University of Massachusetts Amherst, United States.

[*] Corresponding author.

Email: j.a.wright@soton.ac.uk

Abstract

Consumption of products packaged in plastics is growing in Sub-Saharan African cities, whose populations often lack solid waste management (SWM) services. The informal sector often fills gaps in plastic waste collection and recovery that are not met by formal SWM.. To reduce plastic waste mismanagement and its associated health risks, SWM systems are needed that separate plastic waste for reuse or recycling. However, little is known about the contributions and challenges facing the mixed formal-informal enterprises in such cities' SWM chains. This multi-country qualitative study therefore aimed to contrast the specific challenges of plastic waste collectors with those facing general waste collectors. Eleven focus group discussions, 6-12 participants per FGD (n=87 in total), were held with waste collectors in Greater Accra, Ghana, and Kisumu, Kenya, differentiating business types and plastic from mixed waste enterprises. Barriers, enablers, and solutions were identified through thematic coding and analysis of transcripts. We find differing SWM trajectories in Accra versus Kisumu. Domestic waste separation remains low in Kisumu, whilst Accra's SWM system evolved from flexible to rigid plastic separation. Mixed and plastic waste collectors reported shared challenges, such as lack of crushing or transport equipment, harassment, greater hazardous faecal matter in waste streams, and lack of societal recognition. Additionally, specialist plastic waste workers in Greater Accra reported business-specific challenges, notably price volatility from seasonally variable plastic waste generation and from bulk international waste plastic imports, alongside theft of separated plastics. Accra's informal plastic waste sub-collectors were mostly elderly women who reported occupational health challenges from bending and lifting waste. Our findings highlight the need for context-sensitive plastic SWM programmes, including fair bargaining and price regulation measures, occupational health programmes and safer diaper SWM.

Introduction

By 2050, global waste generation will increase by a projected 70% if prompt action is not taken [1]. Of total municipal solid waste (MSW) generated globally in 2022, 82% was collected and 55% managed in controlled facilities [2]. Waste generation is driven by rapid urbanization, industrialization, and population growth [3]. As urbanization increases, the world's cities are struggling to cope with mounting MSW. Urban Africa produced 124 million tonnes of waste in 2015, anticipated to reach 368 million tonnes by 2040 [4]. However, in Sub-Saharan Africa (SSA), average municipal collection rates were 44% in 2016, with 69% of waste openly dumped [1]. Plastics constitute a significant and persistent component of MSW

that poses substantial environmental challenges worldwide. An estimated 4.8 to 12.7 million tonnes of plastic enter the oceans annually, equivalent to a truckload per minute [5].

When single-use plastic products are discarded without appropriate management, micro-plastics are ultimately produced [6], increasing human exposure both directly and through their bioaccumulation in living organisms [7]. Open waste dumping also increases local flood risk by clogging drains, whilst burning degrades air quality [8]. Sustainable Development Goal (SDG) target 11.6 seeks to “reduce the adverse per capita environmental impact of cities”, particularly by increasing the proportion of MSW collected and managed in controlled facilities (indicator 11.6.1).

In low and middle-income countries (LMICs), solid waste management (SWM) is characterized by unorganized waste separation, low collection efficiency, inefficient transportation, low recycling and inappropriate landfill [9], with ineffective, fragmented SWM frameworks. Mismanaged solid waste, including plastics, therefore accumulates in the environment, especially in off-grid areas.. In cities lacking comprehensive municipal recycling systems, informal waste collectors reclaim recyclables (including plastics) and input raw materials into the formal recycling chain [10], thereby reducing air pollution and water contamination in low-income areas [11, 12]. Such solid waste picking and collection also provides livelihood opportunities [13]. Reasons for becoming informal waste pickers include poverty, lack of formal job opportunities, social inequality, and socio-economic constraints such as lack of parental care for youths [14, 15]. Waste pickers often include women, children and the elderly [1, 11]. Although informal waste pickers are integral to SWM systems, their disadvantaged status excludes them from formal labour markets [16]. Despite the significant contribution of informal solid waste collectors, their role in SWM is still not acknowledged in

formal sector management frameworks. Authorities often either ban or ignore informal waste pickers when designing SWM policies and programs [17].

Most existing LMIC-based studies focus on municipal SWM systems [13]. Fewer studies focus on the entrepreneurial synergies underpinning informal waste collector involvement in the LMIC SWM chain due to lack of documentation, record-keeping or sampling frames for informal waste enterprises [13]. Furthermore, there is little evidence concerning informal waste sector adaptation to the growing plastic component within MSW in LMIC cities. Some studies suggest that informal collector engagement can accelerate recovery of recyclables [18, 19], whilst others highlight how waste separation initiatives marginalise informal collectors [20].

This study therefore aims to assess the differing motivations for entering the waste sector, perceived challenges, and proposed solutions, comparing specialised plastic waste workers versus those working in the general (mixed) waste sector. In doing so, it compares waste worker experiences in two African cities (Accra, Ghana and Kisumu, Kenya) with similar formal-informal SWM chains, but contrasting policy regimes and differing plastic business specialisation levels.

Materials and methods

Study design overview

The study followed a convergent parallel mixed methods design [21], comprising a cross-sectional waste collector questionnaire survey in Greater Accra only, and focus group discussions (FGDs), conducted in both Greater Accra and Kisumu. This paper presents the qualitative study component.

Study sites

Fieldwork took place in urban Greater Accra region (Fig 1a), Ghana's administrative capital with a population of approximately 5.0 million in 2021 [22]. From 1994 to 2014, the region's daily MSW generation increased three-fold to 0.70kg/household [23], equivalent to 7,000 tonnes/day in total. Waste collection services covered 68% of Greater Accra's urban households in 2021 [24]. Formal waste collection service coverage in Accra has declined, with informal collection coverage growing [25]. Urban Ghana has an extensive sachet water industry [26], selling water in 500mL plastic bags and generating associated waste. According to World Economic Forum, Ghana produces 840,000 tonnes of plastic waste annually, however, only 9.5% is collected for recycling [27]. There are no established formal systems for plastic waste collection or separation, but sachet manufactures have been active in a voluntary extended producer responsibility initiative, the Plastic Waste Management Project [28] . Ghana raises excise duty on semi-finished and raw plastics but has not enforced a single-use plastics ban, despite introducing a partial carrier bag ban in 2015 [29]. Slum mapping identified 78 slum communities within the city in 2000, though their distribution has subsequently changed [30].

Fig 1. Maps showing (a) Districts in Greater Accra Region, Ghana and (b) Sub-counties in Kisumu County, Kenya (data source: Geoboundaries, https://www.geoboundaries.org/).

Fieldwork also took place in Kisumu city (Fig 1b), Kenya's third largest city with a population over 500,000 in 2019 [31]. Over 60% of its population live in densely populated informal settlements, lacking adequate electricity, water, sanitation and waste collection [32]. Kisumu generates approximately 400 tonnes/day of MSW. Estimates vary, but only 20%-45% of its MSW is recycled, reused or processed to yield economic or ecological benefit [33], well below the National Environment Management Authority's 80% target (NEMA) [34]. The remaining

55%-80% enters the environment or accumulates in municipal or illegal dump sites [35]. Of total collected MSW, 65% is organic and 27% recyclable, so MSW collection and handling poses a significant challenge for Kisumu [33]. With limited municipal service coverage, informal waste-pickers are the backbone of Kisumu's MSW system, but represent a marginalized and impoverished segment of society [36]. In contrast to Ghana, Kenya banned some single-use plastics in 2017, particularly carrier bags [37].

Initial mapping of SWM chains to inform sampling

Both cities' SWM systems were mapped (Fig 2) in consultation with representatives of the Kisumu Waste Actors Network (KIWAN), a formal and informal enterprise network, and the Plastic Waste Management Project and Plastic Waste Collectors' Association (PWCA) in Greater Accra. This mapping then informed context-sensitive FGD sampling strategies.

Fig 2. Waste generation sites, waste chain enterprises, processing and flows in Kisumu (A-C: enterprise types sampled for FGDs).

Fig 3. Waste generation sites, waste chain enterprises, processing and flows in Greater Accra (A-C: enterprise types sampled for FGDs).

Both cities' SWM systems comprise both formal and informal enterprises (Fig 2). Small-scale informal enterprises are usually involved in initial waste collection, whilst large-scale formal enterprises typically handle aggregated and processed waste. Plastic reuse is more widespread in Kisumu, whilst recycling is more common in Greater Accra. This reflects widespread consumption of sachet water (water sold in 500mL plastic bags) in Accra [26], generating flexible plastic waste unsuitable for reuse. In contrast to Accra, Kisumu does not generate sufficient plastic waste to support its own recyclers or manufacturers, so its plastic waste is shipped to Nairobi. In Accra, household and business plastic waste separation is common at source because of plastic's resale value, but not in Kisumu. Consequently, plastic

waste specialisation in Accra extends to the point-of-generation, unlike in Kisumu. Accra's mixed SWM enterprises are more formalised than those handling separated plastics. A corporate provider, ZoomLion, dominates Accra's mixed waste, providing large-scale waste solutions and managing transfer stations. It is a member of a formal sector association, the Environmental Service Providers' Association (ESPA). Informal waste workers in both cities also have representative organisations such as the Kisumu Waste Pickers Welfare Association (KIWAPWA). Its membership was approximately 250 in 2024 [38], but inherent difficulties quantifying the scale of informal waste collection make estimating percentage association membership challenging. There are at least seven recycling companies in Greater Accra, compared to one in Kisumu, with much recovered waste material from Kisumu shipped elsewhere.

This contextual understanding (Fig 2) then informed FGD recruitment. In Accra, recruitment targeted:

A. ***Plastics sub-collectors:*** informal entrepreneurs collecting plastic waste from public spaces, shops, households, and dumpsites, selling plastics to main collectors.
B. ***Plastics main collectors:*** often formal enterprises buying plastic waste from sub-collectors, sometimes separating plastics themselves, and aggregating plastics for sale to recyclers.
C. ***General waste collectors:*** informal small-scale entrepreneurs (termed "aboboya" after their tricycles) charging households or businesses for collecting mixed waste, incurring disposal costs at municipal or ZoomLion dumpsites thereafter. These enterprises occasionally separate valuable waste.

In Kisumu, FGD recruitment contrasted apex plastic traders with less specialised businesses closer to the point-of-generation:

A. **Waste sub-collectors**: Informal entrepreneurs picking valorised waste (e.g. PET, metals, glass bottles) from public places, shops, and households, aggregating and selling waste to main collectors.

B. **Waste intermediaries**: More formalised enterprises that buy various waste types from sub-collectors, aggregate and/or crush the waste, then transport and sell processed waste to apex traders

C. **Apex plastic traders**: Formal businesses who buy aggregated, processed plastic waste from intermediaries, selling it to recycling plants in Nairobi.

In both cities, the study excluded dumpsite scavengers and managers, plastics reusers, and recyclers.

Sample size and participant selection

Planned sample size for Greater Accra was 60 participants. This comprised 24 main collectors, 24 sub-collectors and 12 general waste collectors. Eligible waste collectors operated in Enumeration Areas (EAs) meeting one or more UN-Habitat slum criteria [39]. Most households in eligible EAs lived in over-crowded or non-durable housing, lacked improved sanitation, water sources, or secure tenure. Participants operated in nine of 14 metropolitan authorities (Fig 1a) encompassing 18 such randomly selected EAs. General collectors were randomly sampled from registration records for each selected metropolitan authority, provided by authorities' environmental health teams. Plastic main collectors were randomly sampled from PWCA registration records, created by PWCA district groups. Referral sampling was used to select sub-collectors: each participating main collector was asked to identify at least one sub-collector from whom they purchased plastics. Only waste collectors aged 18 years or older were eligible to participate.

In Kisumu, planned sample size was 30-60 participants, comprising 6-12 participants in two FGDs each for sub-collectors and intermediaries, and one apex trader FGD. Participants were initially randomly sampled from a KIWAN membership list, with subsequent top-up sampling via referral from selected intermediaries and through field reconnaissance at waste disposal/transfer sites. Very few apex traders operate in Kisumu, so a small group meeting was held with the only two apex traders recruited. Otherwise, 13 waste-pickers and 12 intermediaries were recruited, giving 27 participants in total. Eligible participants were adults serving slums in Kisumu.

Data collection

In Greater Accra, six FGDs were organised with two groups each of 6-12 plastic main collectors, plastic waste sub-collectors and general waste collectors. The FGD topic guide explored business establishment and history, SWM operations, and enablers/barriers. The guide was pre-tested among informal waste collectors at a waste transfer site.

PWCA leaders facilitated introductions to their membership. Participants were recruited and invited via phone to a church hall near a recycling plant and PWCA headquarters for informed written consenting. Eight trained qualitative researchers, comprising four females and four males with 2 to 15 years' field experience, conducted the FGDs. Authors E.E.B., R.H., J.O., J.A., A.A and G.A.M hold master's degrees in public health or health-related fields; M.A., R.T.A., E.N.A., and G.S. hold bachelor's degrees in information technology or health-related fields. Two FGDs were conducted concurrently in English, but sometimes in Ga, Twi, and Ewe where necessary. E.E.B., R.H., E.N.A., G.A.M., and A.A. facilitated discussions, R.H., E.E.B. and G.S. took notes alongside audio-recording, whilst J.O., R.T.A., E.N.A., and J.A. supported logistics. M.A. supervised all FGDs, which last approximately two hours and occurred from 26th September to 5th October 2022.

In Kisumu, the Accra topic guide was pre-tested with waste pickers not selected for the main study. Four FGDs were organised with two groups each of waste pickers and intermediaries, and a small group discussion with two of Kisumu's apex traders. Participants were recruited via phone and invited to meetings at a conveniently located community hall and vocational training centre for informed written consenting. Fieldwork took place from 2nd-3rd October 2023, with discussions lasting 58 to 104 minutes. C.O first invited participants to set ground rules, then facilitated discussions. Discussions were predominantly in English, but sometimes in Kiswahili or Luo, depending on participant preference. A.L.O, H.A.A, and B.B.C took notes to supplement audio-recordings. The Kisumu qualitative research team have between 3-15years of qualitative field experience. D.A facilitated logistics, with J.O.O and L.O observing each session.

Qualitative data management, processing and analysis

All audio-recorded FGDs were transcribed verbatim, translated into English if required, and complemented with field notes. Thematic analysis employed both deductive and inductive processes [40]. R.H and E.E.B thematically encoded Accra transcripts using NVivo version 12 [41], with deductive codes representing specific business operations or life cycle stages. Each coder identified themes independently, then met to agree final thematic encoding. Findings were presented to Accra participants via telephone for member-checking, but there were no participant requests for changes. In Kisumu, C.O transcribed interviews, with RH thematically coding transcripts, but without member checking. L.O and J.O reviewed transcripts against audio recordings. Major themes differentiated opportunities, solutions and challenges, with emerging sub-themes identified inductively. Themes were compared between FGDs, with the most engaging extracts selected to illustrate key themes.

Ethical approval

The study was approved by the Faculty of Environmental and Life Sciences Ethical Review Committee, University of Southampton, UK (reference: 55755; approval date 19th August 2020), the Jaramogi Odinga Oginga University of Science and Technology Ethical Review Committee (reference: ERC/23/6/20-4, approval date: 17th August 2020) and the Institutional Review Board of the Noguchi Memorial Institute for Medical Research, University of Ghana (Ref: 003/20-21; approval date: 2nd September 2020).

All study participants provided written informed consent prior to the data collection. Participants were provided with the participant information documents, which was also explained to them in their preferred local languages (Twi, Ewe and Ga for Accra participants, Kiswahili or Luo for Kisumu participants). All study participants were informed about the aim and benefits of the study, as well as their rights as participants. All consenting participants signed (or placed a thumbprint) on an informed consent form before participating in the study. They were assured of confidentiality and that all the information they have provided will be reported with complete anonymity.

Inclusivity in global research

Additional information regarding the ethical, cultural, and scientific considerations specific to inclusivity in global research is included in the Supporting Information (Inclusivity-in-global-research-questionnaire_final.docx).

Results

Participant characteristics

In Greater Accra, participating plastic main collectors were the most educated group, predominantly men, and younger than general collectors,(Table 1). Plastic sub-collectors

were older, largely women, and typically the least educated participants. General waste collectors were all men and generally older.

Table 1. Socio-demographic characteristics of FGD participants (Greater Accra, Ghana)

Participant characteristics	General waste collectors		Plastics sub-collectors		Plastics main collectors		Total	
**Sex**	**N**	**%**	**N**	**%**	**N**	**%**	**N**	**%**
Male	12	100.0	3	12.5	19	79.2	34	56.7
Female	0	0.0	21	87.5	5	20.8	26	43.3
**Age (years)**								
<20	0	0.0	0	0.0	0	0.0	0	0.0
20-29	1	8.4	2	8.3	4	16.7	7	11.7
30-39	3	25.0	3	12.5	7	29.2	13	21.7
40-49	4	33.3	8	33.3	7	29.2	19	31.7
50+	4	33.3	11	45.8	6	25.0	21	35.0
**Educational Level**								
No formal Education	3	25.0	3	12.5	2	8.3	8	13.3
Primary/Junior High School	4	33.3	15	62.5	5	20.8	24	40.0
Middle/Secondary/Senior High School	5	41.6	6	25.0	16	66.7	27	45.0
Tertiary	0	0.0	0	0.0	1	4.2	1	1.7
**Total**	**12**		**24**		**24**		**60**	

Relative to Accra, in Kisumu, whilst the gender balance was similar, participants were younger (mostly <40 years old) with more formal education. Most had completed high school, college or university education (Table 2). The two apex traders had university or college education.

Table 2. Socio-demographic characteristics of FGD participants (Kisumu, Kenya)

Participant characteristics	Waste sub-collectors		Intermediate waste traders		Plastic waste apex traders		Total	
**Sex**	**N**	**%**	**N**	**%**	**N**	**%**	N	%
Male	5	38.5	8	66.7	1	50.0	14	51.9
Female	8	61.5	4	33.3	1	50.0	13	48.2
**Age (years)**								
20-29	4	30.8	7	63.6	2	100.0	13	50
30-39	7	53.9	2	18.2	0	0.0	9	34.6
40-49	1	7.7	1	9.1	0	0.0	2	7.7
50+	1	7.7	1	9.1	0	0.0	2	7.7
**Educational Level**								
N/A	0	0.0	1	8.3	0	0.0	1	3.7

Primary School	0	0.0	1	8.3	0	0.0	1	3.7
High School	5	38.5	5	41.7	0	0.0	10	37.0
College	7	53.9	4	33.3	1	50.0	12	44.4
University	1	7.7	1	8.3	1	50.0	3	11.1
**Total**	**13**		**12**		**2**		**27**	

Business entry motivations for plastic versus general waste collectors

Table 3. Themes reflecting opportunities and motivations for waste sector entry, by city and FGD group

Opportunity or motivation	Kisumu SWM groups	Greater Accra SWM groups
Income-earning potential relative to other options	Sub-collectors; Intermediaries	General collectors; Plastic sub-collectors; Plastic main collectors
Contribution to environmental quality	Sub-collectors; Intermediaries; Apex plastic traders	Plastic sub-collectors; Plastic main collectors; General collectors
External support for business start-up, e.g. vehicles, equipment through CSR	Sub-collectors; Intermediaries; Apex plastic traders	General collectors; Plastic sub-collectors; Plastic main collectors
Opportunities from changing waste composition, segregation, and valorisation	Sub-collectors; Intermediaries; Apex plastic traders	General collectors; Plastic main collectors
Flexible working	Sub-collectors	Plastic main collectors
Low capital cost of business entry	Sub-collectors	Plastic sub-collectors

For almost all FGDs (Table 3), greater economic opportunity, relative to formal employment or other informal livelihoods, was the main driver for entering the waste sector. For example, an Accra general collector noted:

> *"I have worked for 10 years. I trained as an (name of occupation), but I could not get a job. Someone gave me a tricycle to help me collect waste. Initially, I wanted to load foodstuff at [name of market] with the tricycle but then he convinced me to do waste collection. I realized that the business is very profitable because I could buy food and still get some money after I had bought fuel.* ***(R5B, Male, General collector, Accra***)

Across all groups, many participants were motivated by their operations' environmental and sanitation benefits:

> *"I collect the sachet 'rubbers' [i.e. plastic packaging] because when the wind blows it and it rains, it makes the environment dirty. So, I collect them to make the environment clean."* ***(R5A, Female, Sub-collector, Accra)***

All groups reported receiving some external support from government, NGOs, or corporate social responsibility programmes. For sub-collectors, support comprised occupational health education, but donors sometimes provided other groups with vehicles.

Almost all groups highlighted emerging markets for a wider range of plastics, alongside Accra's established markets for water sachet and PET waste:

> ***"****When I started there was a rubber known as "Panyin di Panyin" that we were collecting before sachet water was introduced into the system…then the PET bottle. Even though there was collection of broken plastic chairs and bowls, they were not common… but now they are rampant"* ***(R10B, Male, Main collector, Accra)***

Plastic main collectors attributed the growing range of valorised plastics to greater crusher/pelletiser availability at waste transfer/recycling stations.

General collectors also noted opportunities from more plastics in the domestic mixed waste stream, reflecting changing urban lifestyles:

> *"Initially, you hardly noticed sachet rubbers and PET bottles but now they are a lot. Even the organic waste has reduced now. Maybe it's because there are lots of companies producing plastics"* ***(R6A, Male, General collector, Accra)***

Group-specific themes:

Plastic sub-collectors noted lower start-up costs and security risks, relative to other informal livelihood options:

> *"The reason why I collect the sachet rubbers is that I used to sell [name of commodity] …and I was robbed of all my money. But I have no other means to get money to start over, so someone told me that, if I pick the sachet rubbers, he will buy them, so I got involved. I have been picking the rubbers since 2016 and it has helped me to get money and support my family."* ***(R7A, Male, Sub-collector, Accra)***

Some plastic main collectors also cited flexible working hours as a business entry motivator:

> *"I am not working for anybody. I go to work when I choose to. Secondly, I can work for five hours and rest at any time, I can sell the plastic at any time and get my profit. Because it is my own business, I can make time to go for lectures too because am a student"* ***(R6A, Male, Main collector, Accra)***

Challenges for plastic versus general waste collectors by business operation

Table 4 summarises themes concerning challenges for waste collection business for Accra and Kisumu respectively. Common themes recurring across different business types are presented initially, followed by business-specific challenges.

Table 4. Themes representing challenges for plastic versus general waste collectors by business phase or operation type, identified via FGDs in Greater Accra, Ghana and Kisumu, Kenya

**Major theme – business operation / Sub-themes**	**Kisumu SWM groups affected**	**Greater Accra SWM groups affected**
**Generic challenges**		
Lack of public recognition/respect	Sub-collectors; Intermediaries	General collectors; Plastic main collectors
Harassment, intimidation and gendered stigma	Sub-collectors; Waste intermediaries; Plastic apex traders	General collectors
**Business entry/establishment**		
Lack of trade association		Plastic sub-collectors
Business registration cost and time investment	Sub-collectors; Intermediaries	Plastic main collectors
Lack of capital	Intermediaries	
**Waste collection**		

Competition from informal/illegal collectors	Sub-collectors; Intermediaries	General collectors
Competition from similar businesses	Sub-collectors; Plastic apex traders	Plastic sub-collectors; Plastic main collectors
Occupational health risks from sharp objects, faecal matter in waste	Sub-collectors; Intermediaries	General collectors; Plastic main collectors
Occupational health risks from bending to pick / carrying waste		Plastic sub-collectors
Lack of personal protective equipment/tools	Sub-collectors; Intermediaries	General collectors; Plastic sub-collectors
Payment delays/defaulting by clients	sub-collectors; intermediaries	
Lack of household waste separation	Sub-collectors	
**Processing, storage, & transport**		
Lack of crushing equipment	Intermediaries	General collectors; plastic main collectors
Transportation costs (fuel/vehicle maintenance)	Sub-collectors; Intermediaries	General collectors; Plastic sub-collectors; Plastic main collectors
Remoteness of dump sites	Sub-collectors; Intermediaries	General collectors
road restrictions for waste vehicles	Sub-collectors	General collectors
Lack of secure storage with consequent waste theft risk	Intermediaries	Plastic sub-collectors
**Onward disposal**		
Monopolistic pricing, unfair bargaining; delayed payments	Sub-collectors; Intermediaries; Apex plastic traders	General collectors; Plastic main collectors; Plastic sub-collectors
Price elasticity from large plastic wate loads from neighbouring countries		Plastic main collectors
Heavier waste in wet season incurring higher charges		General waste collectors
Increased dry season sachet consumption, lowering plastic prices		Plastic sub-collectors; Main collectors
Wet season flooding of recycling plants		Plastic main collectors
Queues when offloading waste at dump sites		General waste collectors

Generic, shared challenges

Most groups faced lack of respect:

"…people despise us because of the work we do, they feel this is dirty work and think we are mad people" ***(R2B, Male, Waste Intermediary, Kisumu)***

Many groups described harassment from authorities during waste transportation and drop-off:

> *"… the police if they stop you and you have your license and every other document, they will still charge you 300 Ghana cedis (approx. US$20.5). If you refuse to pay, they will threaten to send you to court even though they will not proceed to court. So, as we are helping the nation to collect the waste, the police are stopping us to extort monies from us …"* ***(R3A, Female, Main collector, Accra)***

Several general waste collectors believed that municipal regulations prohibiting collectors' tricycles on Accra's highways exacerbated harassment. However, both Kisumu's sub-collectors and Accra's general collectors recognised their associations' roles in addressing harassment:

> *"As long as you are a member of the KIWAN they will always come through if you have a problem. For example, if the NEMA guys arrest you they will always call them and work things out. But the KIWAN association also require that we all have our own licenses but before you get one, they will cover you until when you finally get yours"* ***(R2B, Female, Intermediary, Kisumu)***

Kisumu's female collectors were particularly subject to gender-based intimidation and stigmatisation:

> *"When I first ventured into this, as a woman I faced a lot of challenges. Dealing with male clients is a challenge because of frequent intimidation from men and their spouses. Also, handling our male small pickers who work under us is a challenge*

because sometimes they come to work drunk and even insult you. There are times they steal our clients' stuff and you are forced to pay... Some men insist that you go inside their houses to collect the money, and some women even think you are having an affair with their husbands" ***(R4B, Female, sub-collector, Kisumu)***

Business entry challenges

Reported business start-up challenges varied by waste enterprise type. Kisumu collectors and Accra's plastic main collectors highlighted complex, costly business registration processes:

"The process is not easy because it requires registration with different bodies which is really hectic like NEMA, and I think NEMA is the biggest challenge. They take long before they approve your business and there are so many evaluation processes. Then there is the county environment department which you must also register with. They must assess if the waste collection point or yard is a health hazard to the population in the surrounding area. So, I think the process is not easy, complicated and capital intensive" ***(R3A, Female, Waste sub-collector, Kisumu)***

Accra's plastic sub-collectors described how their informal status and lack of collective representation impaired their ability to collectively bargain or mobilise support, relative to plastic main collectors. Kisumu's intermediaries reportedly also lacked start-up capital.

Waste collection challenges

All groups highlighted increased competition as their main waste collection challenge. For Accra's plastic sub-collectors, competitors comprised new market entrants attracted by greater plastic valorisation:

"Nowadays even at dawn, you will find people calling their children to go out and start collecting the waste. ….There are many people in the business now and it has made it seem like the business is going down. So now I don't get as much as I used to. People now even collect with children when their children are even supposed to be in school" ***(R11A, Female, Sub-collector, Accra)***

In contrast, both cities' general waste collectors were concerned that unregistered collectors illegally dumping mixed waste, undercut their prices:

"…We also have others collecting waste at very low prices therefore hindering business; some collectors agree to collect at as low as 20KSh (US$0.15) while I collect maybe at 50KSh (US$0.39), yet they end up dumping the collected at illegal sites; yet I take waste I have collected all the way to Kasese dump site. They feel that we are expensive, therefore look for the cheaper version. That is why you find that there is a lot of illegal dumping and they get more business with it" ***(R4A, Male, Waste sub-collector, Kisumu)***

Both cities' groups reported occupational health risks during waste collection from hazardous sharp objects but increasingly also faecal contamination risks from absorbent hygiene products in waste streams:

"...When you pick up plastics from them, you can sometimes find diapers, used sanitary pads, faeces wrapped in black polythene, sputum etc. They also place the rubber close to their toilets for you to pick up." ***(R12A, Male, Main collector, Accra)***

Accra's plastic sub-collectors, mostly elderly women (Table 1), were concerned about treatment costs for musculo-skeletal injuries from picking and carrying waste:

"For me, the bending and picking all the time can be injurious to the body...Because no matter how much you can sell it for, if you develop a waist problem, the hospital and

treatment will erode all your profits and it wouldn't even be enough for the full treatment" ***(R2A, Female, Sub-collector, Accra)***

Kisumu's sub-collectors reported spending excessive time separating household waste, given how few households practiced waste separation. They additionally attributed problems with payment delays and defaulting to lack of societal recognition:

> *"… customers don't want to pay and do not value the waste collections business. People still believe it is the city council work, therefore don't see the need for paying"* ***(R6A, Male, Waste Intermediary, Kisumu)***

Waste processing, storage and transportation challenges

For Accra's plastic main collectors and general collectors, the main transport-related challenge was fuel price volatility. Many plastic main collectors and general collectors lacked funds for crushing equipment, exacerbating injury risks when handling waste. A particular challenge for Accra's plastic sub-collectors and Kisumu's waste intermediaries was insecure plastic waste storage and unpredictable waste collection schedules, heightening risk of collected plastics theft.

Onward waste disposal challenges

All participants were concerned about price negotiation. Accra's main collectors reported an inability to collectively negotiate plastic prices given monopolistic pricing by recycling companies:

> *"…there isn't any government-owned plastic recycling company. Most of the recycling companies that exist are foreigners… And so they have monopoly in the system and arbitrarily determine the prices they will pay for the plastics we collect. If they refuse*

to pay more for your plastics, there are not many alternatives, so we are forced to accept those low prices" ***(R11B, Male, Main collector, Accra)***

Low prices at recycling plants reportedly cascaded through waste chains to sub-collectors. Kisumu's apex plastic traders faced competition from Ugandan and Nakuru-based waste brokers from outside the city, who undercut the local plastic waste purchase price. Accra's general waste collectors similarly reported how recent increases in mixed waste fees at dump sites had affected their profit margins.

Seasonal volatility additionally affected Accra's plastic SWM chain. In the dry season, higher water sachet consumption increased sachet plastic waste generation, reducing plastic waste prices. This caused delivery vehicle queues at recycling facilities, delaying processing and payments by recyclers:

> *"… In this time of the season (rainy season), you receive payments instantly. Some other times you get the money in the evening after going through a lengthy process. On days where there are lots of rubbers to be processed during the dry season, you may go two to three days or even a week due to load there. In this time of the season (rainy season), there's a shortage in rubbers, hence there is high demand and quick payment."* ***(R5B, Male, Main collector, Accra)***

Conversely, in the cooler wet season, sachet water production dropped in response to lower consumer demand, thereby reducing sachet plastic waste generation and availability for some main collectors. Some main collectors reported how flooding affected recycling plants in the wet season, with plant closures and subsequent clean-up disrupting plastic waste sales. Meanwhile, general collectors also described greater disposal costs for heavier, water-logged mixed waste during the rainy season, given weight-based pricing.

Plastic main collectors reported how large plastic waste shipments from neighbouring countries depressed prices at recycling plants:

> *"…one of the key challenges is the infiltration of foreigners from Burkina Faso and Niger. When these foreigners bring in their plastics, it causes the sellout price to drop for us. Secondly, because they have large loads of plastics, the respect and recognition that is due to us by the recycling company is not given to us. They come with large articulator trucks (40-footer trucks). Once they start coming in our business goes down."* ***(R10A, Male, Main collector, Accra)***

General waste collectors also encountered year-round delays in offloading waste at the few privately operated disposal sites, sometimes even sleeping overnight in dumpsite queues.

Support Requested by Waste Collectors

Many respondents from different FGDs (Table 5) identified waste worker respect promotion as generating multiple, holistic benefits:

> *"We need to fight the attitude of the community by sensitization of community so that they value waste and waste collectors, change attitudes towards waste and let waste be everybody's responsibility. It is not a one-person business, it is all of us"* ***(R6A, Male, Waste Intermediary, Kisumu)***

Mixed waste collectors requested measures to tackle unregistered collectors illegally dumping waste, particularly government initiatives to:

> *"…deal with illegal dumping, and government and relevant bodies concerned with matters of waste to recognize registered waste actors who do the right thing. Also motivate small waste pickers who most of the time practice illegal dumping so that*

they can also do things the right way. That way they will also rise to the corporate world and get licenses" ***(R4A, Male, Waste sub-collector, Kisumu)***

Plastic waste sub-collectors often requested personal protective equipment (PPE) such as gloves, boots and picking tools to mitigate the occupational health risks of carrying and bending. Many plastic main collectors particularly valued transport provision or support (e.g. relaxation of highway restrictions; fuel subsidies), as it enabled them to increase their coverage areas and customer base.

Plastic main collectors often also requested state-supported plastic recycling companies and general waste collectors state-supported dump sites as measures to address monopolistic pricing. Accra's main collectors highlighted the need for protection from volatility in recycled plastic prices and greater regulation of recyclers to counter unscrupulous price bargaining:

"Government must step into the affairs of this business and help us so that these recycling companies…do not cheat us. The government should get involved and regulate our activities because we don't have an advocate who seeks our welfare. Just imagine sending your plastics that you have bought with money from sub-collectors to go and sell and it is being weighed, and you're prevented from observing figures on the scale…If you want to protest and try to see what is happening, they somehow intentionally reduce your price" ***(R9B, Male, Main collector, Accra)***

Table 5. Solutions to waste enterprise challenges identified by SWM workers in Greater Accra and Kisumu.

Challenge(s)	Solution Theme	Kisumu SWM groups	Greater Accra SWM groups
price negotiation, lack of public recognition, harassment, securing loans, supporting business registration	Formation or strengthening of associations	Intermediaries	
lack of public recognition/respect	Public awareness-raising over benefits of waste enterprises	Intermediaries	General collectors

Limited domestic waste separation	Promotion and community sensitisation on domestic waste separation	Sub-collectors; Intermediaries; Apex plastic traders	
	Inter-sectoral promotion of organic waste fertiliser	Sub-collectors; Intermediaries	
	Government policies to boost plastic recycling	Sub-collectors; Apex plastic traders	Plastic main collectors
Competition from unregulated mixed waste collectors	Policies to address illegal waste dumping	Sub-collectors; Intermediaries	
occupational health risks	Provision of tools/PPE (e.g. pickers, gloves)	Intermediaries	Plastic sub-collectors
	Medical support/screening	Sub-collectors	Plastic sub-collectors
	Extended producer responsibility for diaper SWM	Sub-collectors	
	state-funded accident insurance		General collectors
transport costs & harassment	scrap vehicle permits; lift highway restrictions on waste vehicles; reduced fuel tax for waste collectors	Intermediaries	General collectors; Plastic main collectors
	transport provision (e.g. tricycles, vans)	Waste intermediaries	Plastic sub-collectors; Plastic main collectors; General collectors
	rationalise waste transfer /dump site locations.	Sub-collectors	
Price volatility & fair bargaining	Nationalise/regulate recycling companies or dump sites	Sub-collectors; Intermediaries	Plastic main collectors; General collectors
	regulate/ban waste imports from neighbouring countries		Plastic main collectors
	Price regulation	Sub-collectors; Intermediaries; Apex plastic traders	Plastic main collectors; General collectors

Discussion

Few studies have examined how formal-informal SWM chains in LMICs are evolving to handle separated plastic waste. Our study highlights how specialist plastic waste enterprises face distinct challenges not faced by mixed waste enterprises, in part reflecting workforce socio-demographics. Firstly, relative to mixed waste collectors, Greater Accra's specialist plastic

enterprises are more affected by volatility in plastic waste generation and prices. Plastic SWM chains are susceptible to international price fluctuations for recycled plastics. For example, in the Covid-19 pandemic, lower oil prices reduced virgin plastic prices, thereby depressing international recycled plastic prices [42]. Ecuadorian plastic waste-pickers similarly reported price volatility as their greatest challenge [43]. However, our study highlights how local factors within Accra further exacerbate recovered plastic price volatility. One local driver of price volatility is seasonal variation in water sachet consumption and thus sachet plastic waste generation by Accra's extensive sachet water industry. Accra's recyclers are known to increasingly use imported plastics [44]. We also find periodic bulk importation of waste plastics into Accra from neighbouring countries increases throughput at recycling plants, but sporadically depresses prices for local waste collectors. Accra's status as a regional plastic recycling hub thus creates price volatility for local plastic waste businesses.

A further challenge specific to plastic waste collectors is secure storage of segregated plastic waste. Informal waste collectors face well-documented difficulties in storing and processing collected waste given over-crowding, lack of space and formal planning in slums [10]. However, insecurity in slums, coupled with the value of aggregated plastic waste, further exacerbates this working space issue for informal plastics collectors, making them vulnerable to waste theft. Whilst other studies have reported African waste collectors being susceptible to theft of personal positions when working in crime-prone neighbourhoods [45] and noted equipment theft (particularly of waste bins) [46], theft of valuable separated waste has seldom previously been reported. Conversely, mixed waste collectors are subject to price undercutting by competitors who illegally dump mixed waste. In contrast, given its value, there is no financial incentive to dump plastic waste.

Furthermore, our study shows that Greater Accra's SWM workforce is socio-demographically differentiated. Informal plastic waste sub-collectors are mostly older women, sometimes accompanied by children. This conforms with the well-established U-shaped demographic curve of informal sector participation [47], where the elderly and youth predominate. Consequently, these elderly plastic sub-collectors fear occupational health risks, particularly musculo-skeletal injuries from picking and carrying waste. Kisumu's FGD participants were well educated and generally younger than those in Greater Accra. Many of Kisumu's current waste enterprises emerged from youth groups [36], so the city's youthful waste collector profile likely reflects these origins. The comparatively high educational attainment among Kisumu's waste collectors may reflect rapid expansion of Kenya's higher education system [48] in the early 2000s and subsequent rise in graduate unemployment [49]. It suggests that Kisumu's informal waste sector is less susceptible to some commonly cited barriers to formalisation, namely informal workers' lack of education and skills [50].

All FGDs in both cities reported shared challenges widely reported elsewhere. Notably, participants highlighted lack of societal recognition for their work (Table 4) despite its environmental contribution, echoing many other waste-picker studies [11]. Similarly, harassment by authorities, reported by most FGDs, has been widely reported by informal waste sector workers across SSA and Latin America [51]. Confirming trends in plastic imports to Africa [52], both cities' FGD participants highlighted increasing plastics in MSW composition. Participants also reported increasingly encountering hazardous products within the waste stream, reflecting informal waste-picker concerns over occupational health in many other cities [11]. FGDs reported growing faecal contamination risks from absorbent hygiene products (particularly disposable diapers), confirming recent studies reporting increased diaper waste in urban SSA [53, 54].

Participant motivations for entering the waste sector were broadly consistent between both cities (Table 3), aligning with studies elsewhere. For example, Ecuadorian informal plastic waste-pickers cited flexible working and income generation potential within the sector as key drivers for their livelihood choices [43]. Kisumu participants highlighted need for public awareness-raising concerning waste separation, whereas Greater Accra participants had no such concerns given the practice was already widespread (Table 5). Instead, Accra participants described how plastics recycled expanded from sachet packaging initially to subsequently include more varied plastics. Contrary to most countries where rigid plastics are recycled more than flexible plastics [55], plastic SWM in Accra thus initially focussed on flexible sachet packaging, triggered by the need to handle the waste stream from Ghana's extensive sachet water industry [26]. Thus, SWM transitions in LMIC cities towards incorporating plastics may follow different trajectories to high income countries.

In Accra, plastic waste sub-collectors articulated their need for a representative association, similar to associations representing general waste collectors, the ESPA and the PWCA representing plastics main collectors. FGD participants foresaw benefits from such an association's ability to mediate disputes with authorities (Table 5). Kisumu participants similarly highlighted KIWAN's value as an association. Literature supports these participant-led priorities: many initiatives to support informal waste collectors entail partnerships with waste collector associations and such associations are considered critical to informal collectors gaining recognition [10].

Our findings support proposals to integrate waste pickers as partners in SWM systems to build just, inclusive, and liveable cities [56]. Despite policy and programmatic efforts at informal sector SWM integration in several SSA cities including in Kenya [13], other SSA nations, including Ghana, are yet to develop such policies. Our findings suggest that formalisation

initiatives should support fairer price negotiation and reduce price volatility for informal plastic waste-pickers. For example, a Nairobi-based initiative established a local network of recycling company agents, who bought plastics directly from waste-pickers, by-passing intermediate traders [18]. Alongside savings from purchasing directly from pickers, establishing trust through shared meals with waste-pickers increased waste quality, enabling agents to buy waste for a fixed, year-round flat fee. However, context-sensitive programmatic formulation is critical: although policy-based formalisation and integration of waste-pickers can potentially improve waste-picker livelihoods, such policies vary by LMIC and sometimes fail to provide these benefits [11].

Our findings also identify group-specific forms of support for informal collectors that could be targeted through extended producer responsibility programmes. These include secure storage facilities for segregated waste, crushing equipment, and transportation for plastic main collectors. Although Accra's plastic sub-collectors proposed free PPE provision to address musculo-skeletal injury and contamination risks from hazardous waste, some studies have found that impoverished waste-pickers sell these items to supplement their income[18], undermining PPE's intended benefits. Thus, careful PPE programmatic design is needed to deliver intended benefits, with consideration of other occupational health measures such as medical check-ups, daycare for workers' children, or awareness-raising initiatives.

Our study's limitations include partial sampling of SWM chain actors, creating an incomplete perspective on related challenges. We did not sample dumpsite scavengers or representatives of large end-of-chain formal businesses, such as waste transfer station or recycling plant managers. Child waste pickers were excluded from our study because of informed consenting difficulties. Whilst we avoided the purposive or convenience non-representative sampling strategies commonly used when studying informal SWM chains [43,

57], our sampling frame was reliant on membership registers of waste collector associations and Accra's municipal authorities. These registers are likely to omit small-scale, more informal and part-time waste sector workers. Ambiguity in our enterprise type definitions for planning FGD recruitment may also have affected group composition and thereby dynamics. Our findings indicate a need for further evaluation studies assessing the effectiveness of programmes to protect waste sub-collector occupational health and assessing measures to protect their livelihoods from price fluctuations or unfair bargaining practices. Furthermore, reported volatility in plastic waste flows and prices suggests that the many cross-sectional studies quantifying these properties of informal SWM chains [43, 57, 58] risk mis-representing material flows and waste values. We therefore recommend either longitudinal studies of SWM systems, mixed methods studies to capture temporal variability, or, at minimum, explicit consideration of seasonal and fluctuating economic conditions' impact on cross-sectional study findings concerning waste prices and material flows. Our participants described recent expansion in the range of material types they recovered. This highlights a need to evaluate strategies that incentivise and accelerate informal sector recovery of even more material types. These strategies include waste valorisation, recycle material quality (by encouraging door-to-door collection over dumpsite scavenging and separating hazardous waste components), and minimising waste pickers' search/collection effort (e.g. using ICT) [19].

Conclusion

As African formal-informal SWM chains evolve to handle plastic waste, our study identifies several challenges specific to plastic waste enterprises. Firstly, particularly in Greater Accra, plastic waste enterprises struggle with greater price volatility, with plastic waste prices affected by bulk plastic waste shipments arriving from neighbouring countries, seasonally

variable plastic waste generation (particularly from sachet water), and international oil price variations. Secondly, mixed waste collectors in both cities are subject to price under-cutting from unregistered collectors who dump waste illegally, thereby avoiding disposal fees. In contrast, plastic collectors are inherently less vulnerable to such competition, given the value of plastics. Finally, plastics collectors have greater need for secure waste storage than mixed waste collectors, given separated plastics' value. Furthermore, waste worker demographics interact with urban socio-economic conditions to create context-specific challenges. Greater Accra's plastic sub-collectors are mostly older women, who are thus at risk of musculo-skeletal occupational health conditions when picking and collecting waste. Meanwhile, many of Kisumu's female waste collectors reported gender-aggravated stigmatisation and discrimination.

Our study also confirms well-established shared priorities for informal waste collectors, including the need for greater societal recognition, alongside forming and strengthening waste collector associations. Our participants also highlight increased hazardous faecal matter (e.g. disposable diapers, defecation in bags) within the waste stream as a growing occupational health risk. Collectively, these findings and FGD participants themselves highlight priorities to support informal waste collectors, notably fair bargaining mechanisms, collector association support, safer diaper SWM systems, and occupational health programmes.

Acknowledgements

We are grateful to all stakeholders, including leadership and collectors within the waste management sectors in Kisumu, Kenya and Greater Accra, Ghana who devoted their time to share their experiences with us for the study. We also wish to thank all field staff for their

tremendous work during data collection, and our project steering committees in Greater Accra and Kisumu for their feedback on our findings. We are particularly grateful to Caro Omom for facilitation and transcribing of FGDs in Kisumu.

References

1. Kaza S, Yao L, Bhada-Tata P, Van Woerden F. What a waste 2.0: a global snapshot of solid waste management to 2050. Washington D.C.: 2018.
2. United Nations. 11 Sustainable Cities and Communities: Sustainable Development Goals Extended Report 2022. New York: 2023.
3. Tong YD, Huynh TDX, Khong TD. Understanding the role of informal sector for sustainable development of municipal solid waste management system: A case study in Vietnam. Waste Management. 2021;124:118-27. doi: https://doi.org/10.1016/j.wasman.2021.01.033.
4. UNEP. Africa Waste Management Outlook. Nairobi, Kenya: 2018.
5. Jambeck JR, Geyer R, Wilcox C, Siegler TR, Perryman M, Andrady A, et al. Plastic waste inputs from land into the ocean. Science. 2015;347(6223):768-71. doi: 10.1126/science.1260352. PubMed PMID: WOS:000349221300047.
6. Steinmetz Z, Wollmann C, Schaefer M, Buchmann C, David J, Tröger J, et al. Plastic mulching in agriculture. Trading short-term agronomic benefits for long-term soil degradation? Science of The Total Environment. 2016;550:690-705. doi: https://doi.org/10.1016/j.scitotenv.2016.01.153.
7. Conkle JL, Báez Del Valle CD, Turner JW. Are We Underestimating Microplastic Contamination in Aquatic Environments? Environmental Management. 2018;61(1):1-8. doi: 10.1007/s00267-017-0947-8.
8. Lebreton L, Andrady A. Future scenarios of global plastic waste generation and disposal. Palgrave Communications. 2019;5(1):6. doi: 10.1057/s41599-018-0212-7.
9. Rafey A, Siddiqui FZ. A review of plastic waste management in India – challenges and opportunities. International Journal of Environmental Analytical Chemistry. 2023;103(16):3971-87. doi: 10.1080/03067319.2021.1917560.
10. Dias SM. Waste pickers and cities. Environment and Urbanization. 2016;28(2):375-90. doi: 10.1177/0956247816657302.
11. Morais J, Corder G, Golev A, Lawson L, Ali S. Global review of human waste-picking and its contribution to poverty alleviation and a circular economy. Environmental Research Letters. 2022;17(6):063002. doi: 10.1088/1748-9326/ac6b49.
12. Paul JG, Arce-Jaque J, Ravena N, Villamor SP. Integration of the informal sector into municipal solid waste management in the Philippines – What does it need? Waste Management. 2012;32(11):2018-28. doi: https://doi.org/10.1016/j.wasman.2012.05.026.
13. Oduro-Appiah K, Scheinberg A, Afful A, de Vries N. The contribution of participatory engagement strategies to reliable data gathering and inclusive policies in developing countries: Municipal solid waste management data in the Greater Accra Metropolitan Area of Ghana. African Journal of Science, Technology, Innovation and Development. 2021;13(6):735-46. doi: 10.1080/20421338.2020.1797267.
14. Afon A. A survey of operational characteristics, socioeconomic and health effects of scavenging activity in Lagos, Nigeria. Waste Management & Research. 2012;30(7):664-71. doi: 10.1177/0734242X12444894.
15. Escudero V, Asenjo A, Liepmann H, Pignatti C, Tabasso D. What works: promoting pathways to decent work. Geneva: 2019.

16. Coletto D, Bisschop L. Waste pickers in the informal economy of the Global South: included or excluded? International Journal of Sociology and Social Policy. 2017;37(5/6):280-94. doi: 10.1108/IJSSP-01-2016-0006.
17. Del Pilar Moreno-SÁNchez R, Maldonado JH. Surviving from garbage: the role of informal waste-pickers in a dynamic model of solid-waste management in developing countries. Environment and Development Economics. 2006;11(3):371-91.
18. Gall M, Wiener M, Chagas de Oliveira C, Lang RW, Hansen EG. Building a circular plastics economy with informal waste pickers: Recyclate quality, business model, and societal impacts. Resources, Conservation and Recycling. 2020;156:104685. doi: https://doi.org/10.1016/j.resconrec.2020.104685.
19. Velis CA, Hardesty BD, Cottom JW, Wilcox C. Enabling the informal recycling sector to prevent plastic pollution and deliver an inclusive circular economy. Environmental Science & Policy. 2022;138:20-5. doi: https://doi.org/10.1016/j.envsci.2022.09.008.
20. van Leeuwen J, Surya IRF. Network power and exclusion of informal waste pickers when plastic flows change: A case study of community waste banks in Klaten Municipality in Indonesia. Marine Policy. 2024;167:106285. doi: https://doi.org/10.1016/j.marpol.2024.106285.
21. Cresswell JW, Clark VLP. Designing and conducting mixed methods research. London: Sage Publications; 2017.
22. Ghana Statistical Services. Ghana 2021 Population and Housing Census: Population of Regions and Districts. Accra: 2021.
23. Miezah K, Obiri-Danso K, Kádár Z, Fei-Baffoe B, Mensah MY. Municipal solid waste characterization and quantification as a measure towards effective waste management in Ghana. Waste Management. 2015;46:15-27. doi: https://doi.org/10.1016/j.wasman.2015.09.009.
24. Ghana Statistical Service. Ghana 2021 Population and Housing Census General Report Vol. 3M: Water and Sanitation. Accra: 2022.
25. Oduro-Appiah K, Scheinberg A, Mensah A, Afful A, Boadu HK, de Vries N. Assessment of the municipal solid waste management system in Accra, Ghana: A 'Wasteaware' benchmark indicator approach. Waste Management & Research. 2017;35(11):1149-58. doi: 10.1177/0734242X17727066.
26. Stoler J. From curiosity to commodity: a review of the evolution of sachet drinking water in West Africa. WIREs Water. 2017;4(3):e1206. doi: https://doi.org/10.1002/wat2.1206.
27. World Economic Forum. Data benefits Ghana's fight against plastic pollution Cologny, Switzerland: World Economic Forum; 2023 [19/08/2025]. Available from: https://www.weforum.org/impact/data-benefits-ghana-fight-against-plastic-pollution/.
28. Plastic Revolution Foundation. Developing extended producer responsibility for packaging in Ghana: a brief introduction and status report. Oslo: 2021.
29. Adam I, Walker TR, Bezerra JC, Clayton A. Policies to reduce single-use plastic marine pollution in West Africa. Marine Policy. 2020;116:103928. doi: https://doi.org/10.1016/j.marpol.2020.103928.
30. Engstrom R, Sandborn A, Qin Y, Burgdorfer J, Stow D, Weeks J, et al., editors. Mapping slums using spatial features in Accra, Ghana. 2015 Joint Urban Remote Sensing Event (JURSE); 2015 30 March-1 April 2015.
31. Kenya National Bureau of Statistics. 2019 Population and Housing Census Vol. IV: distribution of population by socio-economic characteristics. Nairobi: 2019.
32. Sibanda LK, Obange N, Awuor FO. Challenges of Solid Waste Management in Kisumu, Kenya. Urban Forum. 2017;28(4):387-402. doi: 10.1007/s12132-017-9316-1.
33. Oloko M, Capuano Mascarenhas L, Ness B, Otiende F. Solid waste management: recommendations for Kisumu, Kenya. Lararegatan, Sweden: 2019 Contract No.: 1.
34. NEMA. The National Solid Waste Management Strategy. Nairobi, Kenya: 2015.
35. Munala G, Moirongo BO. The need for integrated solid waste management in Kisumu, Kenya. Journal of Agriculture, Science and Technology. 2011;13(1):65-78.

36. Gutberlet J, Kain J-H, Nyakinya B, Oloko M, Zapata P, Zapata Campos MJ. Bridging Weak Links of Solid Waste Management in Informal Settlements. The Journal of Environment & Development. 2016;26(1):106-31. doi: 10.1177/1070496516672263.

37. Behuria P. Ban the (plastic) bag? Explaining variation in the implementation of plastic bag bans in Rwanda, Kenya and Uganda. Environment and Planning C: Politics and Space. 2021;39(8):1791-808. doi: 10.1177/2399654421994836.

38. Oduor D. Kisumu Waste Pickers Welfare Association (KIWAPWA): Advocating for Change Nairobi: Muungano Alliance; 2024 [19/08/2025]. Available from: https://www.muungano.net/browseblogs/2024/8/6/7378f4wh26m72ar7sz9r6i36zhcjgm.

39. UN-Habitat. Slum almanac 2015-16 tracking improvement in the lives of slum dwellers. Nairobi: UN-Habitat, 2016.

40. Braun V, Clarke V. Using thematic analysis in psychology. Qualitative Research in Psychology. 2006;3(2):77-101. doi: 10.1191/1478088706qp063oa.

41. Lumivero. NVivo. 12 ed. Denver: Lumivero.; 2017.

42. Selmi R, Hammoudeh S, Kasmaoui K, Sousa RM, Errami Y. The dual shocks of the COVID-19 and the oil price collapse: A spark or a setback for the circular economy? Energy Economics. 2022;109:105913. doi: https://doi.org/10.1016/j.eneco.2022.105913.

43. Hidalgo-Crespo J, Amaya-Rivas JL, Ribeiro I, Soto M, Riel A, Zwolinski P. Informal waste pickers in guayaquil: Recycling rates, environmental benefits, main barriers, and troubles. Heliyon. 2023;9(9). doi: 10.1016/j.heliyon.2023.e19775.

44. Babson S, Mantey P. Plastic waste recycling in Ghana: understanding the sources and value chain. In: Biney I, Boateng J, Nixon P, editors. Sustainable Community Development in Ghana. London: Routledge; 2024. p. 182-203.

45. Mbiba B. Urban solid waste characteristics and household appetite for separation at source in Eastern and Southern Africa. Habitat International. 2014;43:152-62. doi: 10.1016/j.habitatint.2014.02.001. PubMed PMID: WOS:000337775300017.

46. Schenck CJ, Blaauw PF, Viljoen JM, Swart EC. Exploring the Potential Health Risks Faced by Waste Pickers on Landfills in South Africa: A Socio-Ecological Perspective. International Journal of Environmental Research and Public Health [Internet]. 2019; 16(11).

47. Ulyssea G. Informality: Causes and Consequences for Development. Annual Review of Economics. 2020;12(1):525-46.

48. Sifuna DN. Some reflections on the expansion and quality of higher education in public universities in Kenya. Research in Post-Compulsory Education. 2010;15(4):415-25. doi: 10.1080/13596748.2010.526803.

49. Nason B. Youth Unemployment among Graduates of Tertiary Institutions in Kenya. Africa Journal of Technical and Vocational Education and Training. 2019;4(1):84-93. doi: https://doi.org/10.69641/afritvet.2019.4184.

50. La Porta R, Shleifer A. Informality and development. Journal of economic perspectives. 2014;28(3):109-26.

51. Ogando AC, Roever S, Rogan M. Gender and informal livelihoods: Coping strategies and perceptions of waste pickers in Sub-Saharan Africa and Latin America. International Journal of Sociology and Social Policy. 2017;37(7-8):435-51. doi: 10.1108/IJSSP-06-2016-0077.

52. Babayemi JO, Nnorom IC, Osibanjo O, Weber R. Ensuring sustainability in plastics use in Africa: consumption, waste generation, and projections. Environmental Sciences Europe. 2019;31(1):60. doi: 10.1186/s12302-019-0254-5.

53. Thomas-Possee MLH, Shaw P, Bain RES, Hill A, Okotto-Okotto J, Okotto LG, et al. Disposable diaper consumption in Sub-Saharan Africa: estimating the risks of associated unsafe waste. PLOS Sustainability and Transformation. 2024;3(4). doi: https://doi.org/10.1371/journal.pstr.0000106.

54. White HL, Mwapasa T, Mphasa M, Kalonde PK, Feasey N, Oliver DM, et al. Open defaecation by proxy: Tackling the increase of disposable diapers in waste piles in informal settlements.

International Journal of Hygiene and Environmental Health. 2023;250:114171. doi: https://doi.org/10.1016/j.ijheh.2023.114171.
55. Hopewell J, Dvorak R, Kosior E. Plastics recycling: challenges and opportunities. Philosophical Transactions of the Royal Society B: Biological Sciences. 2009;364(1526):2115-26. doi: 10.1098/rstb.2008.0311.
56. Dias B, Santana I, Alves B. Public policies and initiatives for waste minimization and recycling in Brazil. Waste management policies and practices in BRICS nations. Boca Raton: CRC Press; 2021. p. 270-8.
57. Mwanza BG, Mbohwa C, Telukdarie A, Medoh C. Value Addition to Plastic Solid Wastes: Informal Waste Collectors' Perspective. Procedia Manufacturing. 2019;33:391-7. doi: https://doi.org/10.1016/j.promfg.2019.04.048.
58. Kumar A, Samadder SR, Kumar N, Singh C. Estimation of the generation rate of different types of plastic wastes and possible revenue recovery from informal recycling. Waste Management. 2018;79:781-90. doi: https://doi.org/10.1016/j.wasman.2018.08.045.

Supplementary Information Captions

S1 Table. Research team characteristics in Kisumu, Kenya.

Fig 2

Click here to access/download;Figure;Fig2.tif

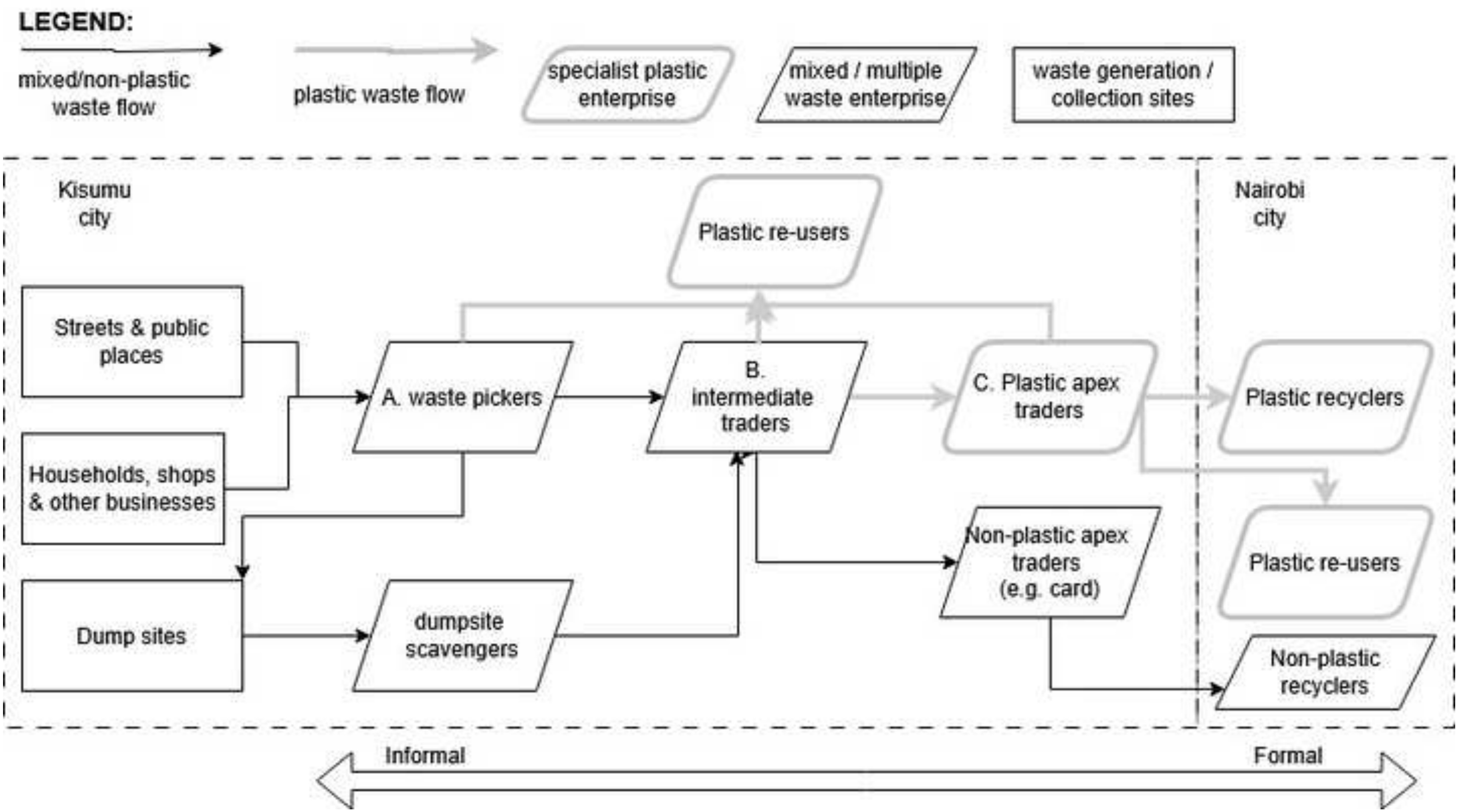

Click here to access/download;Figure;Fig3.tif

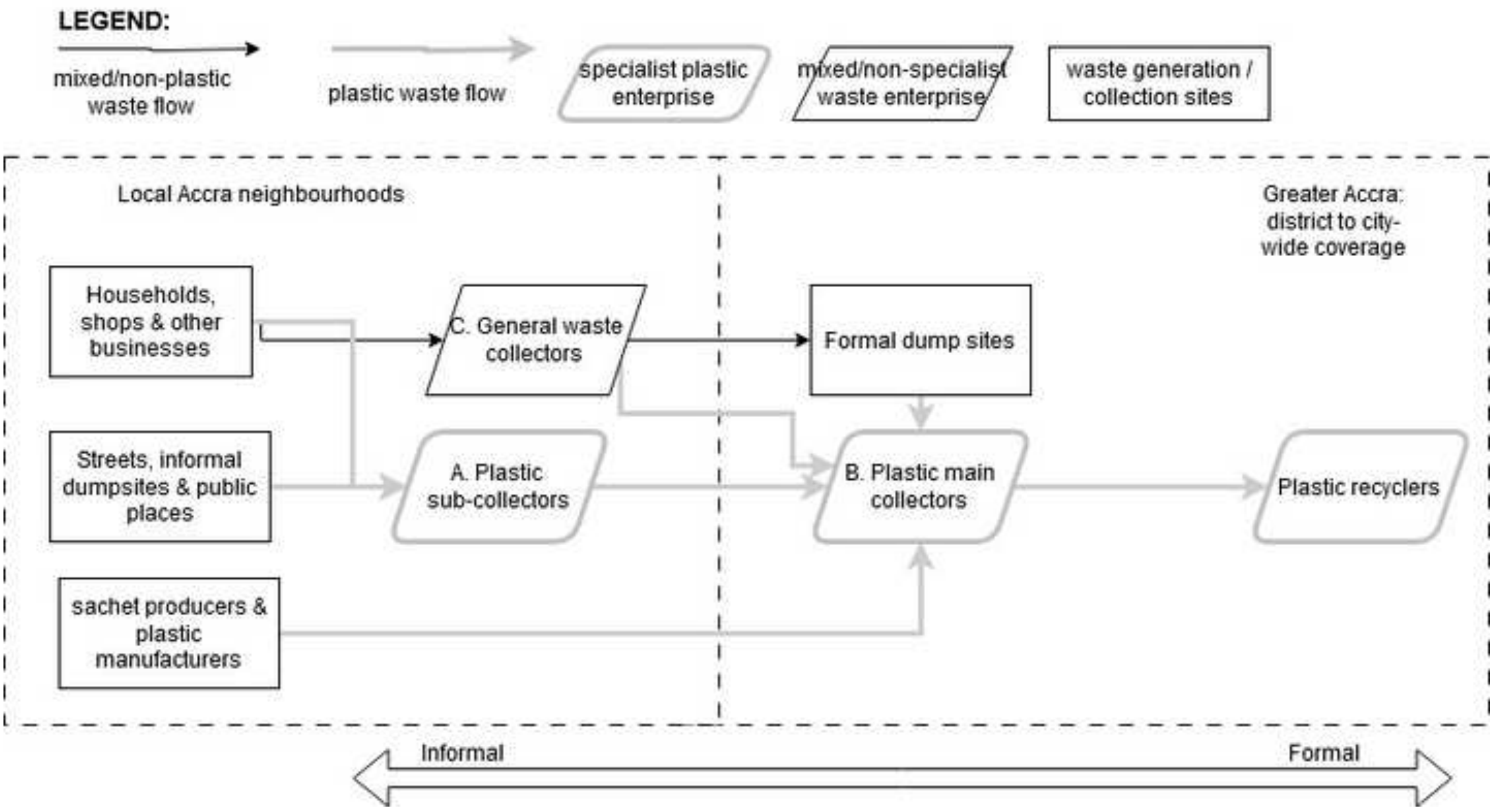

Fig 1

Click here to access/download;Figure;Fig1.tif

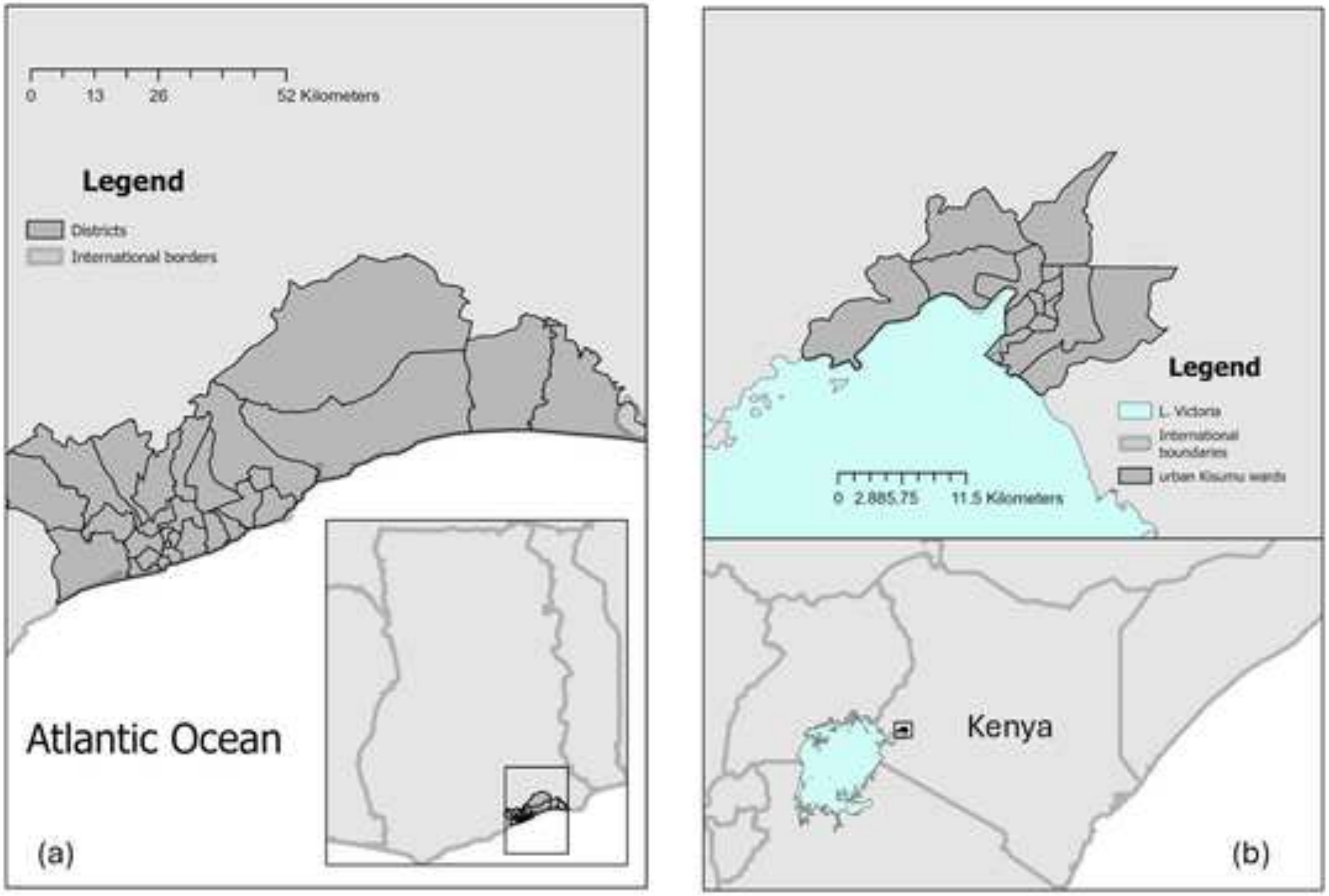

Click here to access/download
Supporting Information
Inclusivity-in-global-research-questionnaire_final.docx

Revised Manuscript with Track Changes

Challenges and opportunities for plastic versus mixed waste enterprises in Greater Accra and Kisumu: A~~a~~ qualitative study

Ruby Hornuvo[1,#a], Eunice Emefa Boafor[1], Lorna-Grace Okotto[2], Joseph Okotto-Okotto[3], Mawuli Dzodzomenyo[1], Moses Asamoah[1, 4], Jim Wright[4*]

[1] School of Public Health, University of Ghana, Legon, Accra, Ghana.

[2] School of Spatial Planning and Natural Resource Management, Jaramogi Oginga Odinga University of Science and Technology, Bondo, Kenya.

[3]Victoria Institute for Research on Environment and Development International, Rabuor, Kenya.

[4]Social Statistics and Demography, University of Southampton, Southampton, UK.

[5] School of Geography and Environmental Science, University of Southampton, Southampton, UK.

[#a]Current address: School of Public Health and Health Sciences, University of Massachusetts Amherst, ~~Arnold House, 715 North Pleasant Street,~~ United States.

[*] Corresponding author.

Email: j.a.wright@soton.ac.uk

Formatted: Font: 18 pt, Bold

Formatted: Font: 18 pt, Bold

Formatted: Font: 12 pt

Formatted: Font: 12 pt

Formatted: Font: 12 pt

Formatted: Font: 12 pt

Abstract

Consumption of products packaged in plastics is growing in Sub-Saharan African cities, whose populations often lack solid waste management (SWM) services. The informal sector often fills ~~the~~gaps in plastic waste collection ~~service~~and recovery ~~gaps in the~~that are not met by formal SWM.~~-~~. To reduce plastic waste mismanagement and its associated health risks, SWM systems are needed that separate plastic waste for reuse or recycling. However, little is known about the contributions and challenges facing the mixed formal-informal enterprises in such cities' SWM chains. This multi-country qualitative study therefore aimed to contrast the specific challenges of plastic waste collectors with those facing general waste collectors. Eleven focus group discussions, 6-12 participants per FGD (n=87 in total), ~~-~~were held with waste collectors in Greater Accra, Ghana, and Kisumu, Kenya, differentiating business types and plastic from mixed waste enterprises. Barriers, enablers, and solutions were identified through thematic coding and analysis of transcripts. We find differing SWM trajectories in Accra versus Kisumu. Domestic waste separation remains low in Kisumu, whilst Accra's SWM system evolved from flexible to rigid plastic separation. Mixed and plastic waste collectors reported shared challenges, such as lack of crushing or transport equipment, harassment, greater hazardous faecal matter in waste streams, and lack of societal recognition. Additionally, specialist plastic waste workers in Greater Accra reported business-specific challenges, notably price volatility from seasonally variable plastic waste generation and from bulk international waste plastic imports, alongside theft of separated plastics. Accra's informal plastic waste sub-collectors were mostly elderly women who reported occupational health challenges from bending and lifting waste. Our findings highlight the need for context-sensitive plastic SWM programmes, including fair bargaining and price regulation measures, occupational health programmes and safer diaper SWM.

Introduction

By 2050, global waste generation will increase by a projected 70% if prompt action is not taken [1]. Of total municipal solid waste (MSW) generated globally in 2022, 82% was collected and 55% managed in controlled facilities [2]. Waste generation is driven by rapid urbanization, industrialization, and population growth [3]. As urbanization increases, the world's cities are struggling to cope with mounting MSW. Urban Africa produced 124 million tonnes of waste in 2015, anticipated to reach 368 million tonnes by 2040 [4]. However, in Sub-Saharan Africa (SSA), average municipal collection rates were 44% in 2016, with 69% of waste openly dumped [1]. Plastics constitute a significant and persistent component of MSW

Formatted: Font: 18 pt, Bold
Formatted: Heading 1
Formatted: Font: 12 pt
Formatted: Font: 12 pt, Font color: Auto
Formatted: Font: 12 pt, Font color: Auto
Formatted: Font: 12 pt, Font color: Auto
Formatted: Font: 12 pt, Font color: Auto
Formatted: Font: 12 pt
Formatted: Font: 12 pt, Font color: Auto
Formatted: Font: 12 pt, Font color: Auto
Formatted: Font: 12 pt
Formatted: Font: 12 pt
Formatted: Font: 12 pt
Formatted: Font: 12 pt
Formatted: Font: 12 pt
Formatted: Font: 12 pt, Font color: Custom Color(RGB(238,0,0))
Formatted: Font: 18 pt, Bold
Formatted: Heading 1, Left, Line spacing: single
Formatted: Font: 12 pt
Formatted: Indent: First line: 0.5"
Formatted: Font: 12 pt
Formatted: Font: 12 pt
Formatted: Font: 12 pt
Formatted: Font: 12 pt
Formatted: Font: 12 pt
Formatted: Font: 12 pt
Formatted: Font: 12 pt
Formatted: Font: 12 pt
Formatted: Font: 12 pt
Formatted: Font: 12 pt

that poses substantial environmental challenges worldwide. An estimated 4.8 to 12.7 million tonnes of plastic enter the oceans annually, equivalent to a truckload per minute [5].

When single-use plastic products are discarded without appropriate management, micro-plastics are ultimately produced [6], increasing human exposure both directly and through their bioaccumulation in living organisms [7]. Open waste dumping also increases local flood risk by clogging drains, whilst burning degrades air quality [8]. Sustainable Development Goal (SDG) target 11.6 seeks to “reduce the adverse per capita environmental impact of cities”, particularly by increasing the proportion of MSW collected and managed in controlled facilities (indicator 11.6.1).

-In low and middle-income countries (LMICs), solid waste management (SWM) is characterized by unorganized waste separation, low collection efficiency, inefficient transportation, low recycling and inappropriate landfill [9], with ineffective, fragmented SWM frameworks. Mismanaged solid waste, including plastics, therefore accumulates in the environment, especially in off-grid areas. ~~The informal sector fills the collection service gaps in formal SWM systems, usually in low-income urban settlements~~. In cities lacking ~~proper~~ comprehensive municipal recycling systems, informal waste ~~pickers or~~ collectors reclaim recyclables (including plastics) and input raw materials into the formal recycling chain [10], thereby reducing air pollution and water contamination in low-income areas [11, 12]. Such solid waste picking and collection also provides livelihood opportunities [13]. ~~Informal sector involvement in many LMICs’ SWM systems has increased due to greater demand for urban SWM services [14-16].~~ Reasons for becoming informal waste pickers include poverty, lack of formal job opportunities, social inequality, and socio-economic constraints such as lack of parental care for youths [17, 18]. -Waste pickers often include women, children and the elderly [1, 11].

Formatted: Font: 12 pt
Formatted: Font: 12 pt
Formatted: Font: 12 pt
Formatted: Font: 12 pt
Formatted: Font: 12 pt
Formatted: Font: 12 pt
Formatted: Font: 12 pt
Formatted: Font: 12 pt
Formatted: Font: 12 pt
Formatted: Font: 12 pt
Formatted: Font: 12 pt
Formatted: Font: 12 pt
Formatted: Font: 12 pt
Formatted: Font: 12 pt
Formatted: Font: 12 pt
Formatted: Font: 12 pt
Formatted: Font: 12 pt
Formatted: Font: 12 pt
Formatted: Font: 12 pt
Formatted: Font: 12 pt
Formatted: Font: 12 pt
Formatted: Font: 12 pt
Formatted: Font color: Auto
Formatted: Font color: Auto

Although informal waste pickers are integral to SWM systems, their disadvantaged status excludes them from formal labour markets [19]. ~~Waste pickers often include women, children and the elderly [1, 11]. Reasons for becoming informal waste pickers include poverty, lack of formal job opportunities, social inequality, and socio-economic constraints such as lack of parental care for youths~~ [17, 18]~~.~~

Despite the significant contribution of informal solid waste collectors, their role in SWM is still not acknowledged in formal sector management frameworks. Authorities often either ban or ignore informal waste pickers when designing SWM policies and programs [20].

Most existing LMIC-based studies focus on municipal SWM systems [13]. Fewer studies focus on the entrepreneurial synergies underpinning informal waste collector involvement in the LMIC SWM chain~~. Similarly, few studies of informal sector dynamics assess the challenges in researching informal waste collectors~~ due to lack of documentation, record-keeping or sampling frames for informal waste enterprises [13]. Furthermore, there is little evidence concerning ~~how the~~ informal waste sector ~~is adapting~~adaptation to the growing plastic component within MSW in LMIC cities. Some studies suggest that informal collector engagement~~s~~ can ~~be engaged to~~ accelerate recovery of recyclables [21, 22], whilst others highlight how waste separation initiatives marginalise informal collectors [23].

This study therefore aims to assess the differing motivations for entering the waste sector, perceived challenges, and proposed solutions, comparing specialised plastic waste workers versus those working in the general (mixed) waste sector. In doing so, it compares waste worker experiences in two African cities (Accra, Ghana and Kisumu, Kenya) with similar formal-informal SWM chains, but contrasting policy regimes and differing plastic business specialisation levels.

Formatted: Font: 12 pt

Formatted: Font: 12 pt

Formatted: Font: 12 pt

Formatted: Font: 12 pt, Font color: Custom Color(RGB(238,0,0))

Formatted: Font: 12 pt, Font color: Custom Color(RGB(238,0,0))

Formatted: Font: 12 pt

Formatted: Font: 12 pt

Formatted: Font: 12 pt

Formatted: Font: 12 pt

Formatted: Font: 12 pt

Formatted: Font: 12 pt

Formatted: Font: 12 pt

Formatted: Font: 12 pt

Formatted: Font: 12 pt

Formatted: Font: 12 pt

Formatted: Font: 12 pt

Formatted: Font: 12 pt

Formatted: Font: 12 pt

Formatted: Font: 12 pt

Formatted: Font: 12 pt

Formatted: Font: 12 pt

Materials and m~~M~~ethods

Study d~~D~~esign o~~O~~verview

The study followed a convergent parallel mixed methods design [24], comprising a cross-sectional waste collector questionnaire survey in Greater Accra only, and focus group discussions (FGDs), conducted in both Greater Accra and Kisumu. This paper presents the qualitative study component.

Study sites

Fieldwork took place in urban Greater Accra region (Fig 1a), Ghana's administrative capital with a population of approximately 5.0 million in 2021 [25]. From 1994 to 2014, the region's daily MSW generation increased three-fold to 0.70kg/household [26], equivalent to 7,000 tonnes/day in total. Waste collection services covered 68% of Greater Accra's urban households in 2021 [27]. Formal waste collection service coverage in Accra has declined, with informal collection coverage growing [28]. Urban Ghana has an extensive sachet water industry [29], selling water in 500mL plastic bags and generating associated waste~~.~~. According to World Economic Forum, Ghana produces 840,000 tonnes of plastic waste annually, however, only 9.5% is collected for recycling [30]. There are no established formal systems for plastic waste collection or separation, but sachet manufactures have been active in a voluntary extended producer responsibility initiative, the Plastic Waste Management Project [31] . Ghana raises excise duty on semi-finished and raw plastics but has not enforced a single-use plastics ban, despite introducing a partial carrier bag ban in 2015 [32]. Slum mapping identified 78 slum communities within the city in 2000, though their distribution has subsequently changed [33].

Fig 1. Maps showing (a) Districts in Greater Accra Region, Ghana and (b) Sub-counties in Kisumu County, Kenya (data source: Geoboundaries, https://www.geoboundaries.org/).

Formatted: Font: 18 pt, Bold
Formatted: Heading 1, Left
Formatted: Font: 18 pt, Bold
Formatted: Font: 16 pt, Bold
Formatted: Heading 2, Left, Line spacing: single
Formatted: Font: 16 pt, Bold
Formatted: Font: 16 pt, Bold
Formatted: Font: 12 pt
Formatted: Font: 12 pt
Formatted: Font: 12 pt
Formatted: Font: 16 pt, Bold
Formatted: Heading 2, Left, Line spacing: single
Formatted: Font: 12 pt
Formatted: Font: 12 pt
Formatted: Font: 12 pt
Formatted: Font: 12 pt
Formatted: Font: 12 pt
Formatted: Font: 12 pt
Formatted: Font: 12 pt
Formatted: Font: 12 pt
Formatted: Font: 12 pt
Formatted: Font: 12 pt
Formatted: Font: 12 pt
Formatted: Font: 12 pt
Formatted: Font: 12 pt
Formatted: Font: 12 pt
Formatted: Font: 12 pt
Formatted: Font: 12 pt
Formatted: Font: 12 pt
Formatted: Font: 12 pt
Formatted: Font: 12 pt
Field Code Changed

Fieldwork also took place in Kisumu city (Fig 1b), Kenya's third largest city with a population over 500,000 in 2019 [34]. Over 60% of its population live in densely populated informal settlements, lacking adequate electricity, water, sanitation and waste collection [35]. Kisumu generates approximately 400 tonnes/day of MSW. Estimates vary, but only 20%-45% of its MSW is recycled, reused or processed to yield economic or ecological benefit [36], well below the National Environment Management Authority's 80% target (NEMA) [37]. The remaining 55%-80% enters the environment or accumulates in municipal or illegal dump sites [38]. Of total collected MSW, 65% is organic and 27% recyclable, so MSW collection and handling poses a significant challenge for Kisumu [36]. With limited municipal service coverage, informal waste-pickers are the backbone of Kisumu's MSW system, but represent a marginalized and impoverished segment of society [39]. In contrast to Ghana, Kenya banned some single-use plastics in 2017, particularly carrier bags [40].

Initial mapping of SWM chains to inform sampling

Both cities' SWM systems were mapped (Fig 2~~1~~) in consultation with representatives of the Kisumu Waste Actors Network (KIWAN), a formal and informal enterprise network, and the Plastic Waste Management Project and Plastic Waste Collectors' Association (PWCA) in Greater Accra. This mapping then informed context-sensitive FGD sampling strategies.

Fig ~~1~~2. Waste generation sites, waste chain enterprises, processing and flows in ~~(a)~~ Kisumu ~~(b) Greater Accra~~ (A-C: enterprise types sampled for FGDs).

Fig 3. Waste generation sites, waste chain enterprises, processing and flows in Greater Accra (A-C: enterprise types sampled for FGDs).

Both cities' SWM systems comprise both formal and informal enterprises (Fig ~~1~~2). Small-scale informal enterprises are usually involved in initial waste collection, whilst large-scale formal enterprises typically handle aggregated and ~~,~~ processed waste. Plastic reuse is more

Formatted: Font: 12 pt
Formatted: Font: 12 pt
Formatted: Font: 12 pt
Formatted: Font: 12 pt
Formatted: Font: 12 pt
Formatted: Font: 12 pt
Formatted: Font: 12 pt
Formatted: Font: 12 pt
Formatted: Font: 12 pt
Formatted: Font: 12 pt
Formatted: Font: 12 pt
Formatted: Font: 12 pt
Formatted: Font: 12 pt
Formatted: Font: 12 pt
Formatted: Font: 12 pt
Formatted: Font: 12 pt
Formatted: Font: 12 pt
Formatted: Font: 12 pt
Formatted: Font: 16 pt, Bold
Formatted: Heading 2, Left, Line spacing: single
Formatted: Font: 12 pt
Formatted: Font: 12 pt
Formatted: Font: 12 pt
Formatted: Font: 12 pt
Formatted: Font: 12 pt

widespread in Kisumu, whilst recycling is more common in Greater Accra. This reflects widespread consumption of sachet water (water sold in 500mL plastic bags) in Accra [29], generating flexible plastic waste unsuitable for reuse. In contrast to Accra, Kisumu does not generate sufficient plastic waste to support its own recyclers or manufacturers, so its plastic waste is shipped to Nairobi. In Accra, household and business plastic waste separation is common at source because of plastic's resale value, but not in Kisumu. Consequently, plastic waste specialisation in Accra extends to the point-of-generation, unlike in Kisumu. Accra's mixed SWM enterprises are more formalised than those handling separated plastics. A corporate provider, ZoomLion, dominates Accra's mixed waste, providing large-scale waste solutions and managing transfer stations. It is a member of a formal sector association, the Environmental Service Providers' Association (ESPA). Informal waste workers in both cities also have representative organisations such as the Kisumu Waste Pickers Welfare Association (KIWAPWA). Its membership was approximately 250 in 2024 [41], but inherent difficulties quantifying the scale of informal waste collection make estimating percentage association membership challenging. There are at least seven recycling companies in Greater Accra, compared to one in Kisumu, with much recovered waste material from Kisumu shipped elsewhere.

This contextual understanding (Fig 2~~1~~) then informed FGD recruitment. In Accra, recruitment targeted:

A. ***Plastics sub-collectors:*** informal entrepreneurs collecting plastic waste from public spaces, shops, households, and dumpsites, selling plastics to main collectors.

B. ***Plastics main collectors:*** often formal enterprises buying plastic waste from sub-collectors, sometimes separating plastics themselves, and aggregating plastics for sale to recyclers.

Formatted: Font: 12 pt

Formatted: Font: 12 pt

Formatted: Font: 12 pt

Formatted: Font: 12 pt

C. ***General waste collectors:*** informal small-scale entrepreneurs (termed “aboboya” after their tricycles) charging households or businesses for collecting mixed waste, incurring disposal costs at municipal or ZoomLion dumpsites thereafter. These enterprises occasionally separate valuable waste.

In Kisumu, FGD recruitment contrasted apex plastic traders with less specialised businesses closer to the point-of-generation:

A. **Waste sub-collectors**: Informal entrepreneurs picking valorised waste (e.g. PET, metals, glass bottles) from public places, shops, and households, aggregating and selling waste to main collectors.

B. **Waste intermediaries**: More formalised enterprises that buy various waste types from sub-collectors, aggregate and/or crush the waste, then transport and sell processed waste to apex traders

C. **Apex plastic traders**: Formal businesses who buy aggregated, processed plastic waste from intermediaries, selling it to recycling plants in Nairobi.

In both cities, the study excluded dumpsite scavengers and managers, plastics reusers, and recyclers.

Sample size and participant selection

Planned sample size for Greater Accra was 60 participants. This comprised 24 main collectors, 24 sub-collectors and 12 general waste collectors. Eligible waste collectors operated in Enumeration Areas (EAs) meeting one or more UN-Habitat slum criteria [42]. Most households in eligible EAs lived in over-crowded or non-durable housing, lacked improved sanitation, water sources, or secure tenure. Participants operated in nine of 14 metropolitan authorities (Fig 1a) encompassing 18 such randomly selected EAs. General collectors were

Formatted: Font: 16 pt, Bold
Formatted: Heading 2, Left, Line spacing: single
Formatted: Font: 12 pt
Formatted: Font: 12 pt
Formatted: Font: 12 pt
Formatted: Font: 12 pt

randomly sampled from registration records for each selected metropolitan authority, provided by authorities' environmental health teams. Plastic main collectors were randomly sampled from PWCA registration records, created by PWCA district groups. Referral sampling was used to select sub-collectors: each participating main collector was asked to identify at least one sub-collector from whom they purchased plastics. Only waste collectors aged 18 years or older were eligible to participate.

In Kisumu, planned sample size was 30-60 participants, comprising 6-12 participants in two FGDs each for sub-collectors and intermediaries, and one apex trader FGD. Participants were initially randomly sampled from a KIWAN membership list, with subsequent top-up sampling via referral from selected intermediaries and through field reconnaissance at waste disposal/transfer sites. Very few apex traders operate in Kisumu, so a small group meeting was held with the only two apex traders recruited. Otherwise, 13 waste-pickers and 12 intermediaries were recruited, giving 27 participants in total. Eligible participants were adults serving slums in Kisumu.

Data collection

In Greater Accra, six FGDs were organised with two groups each of 6-12 plastic main collectors, plastic waste sub-collectors and general waste collectors. The FGD topic guide explored business establishment and history, SWM operations, and enablers/barriers. The guide was pre-tested among informal waste collectors at a waste transfer site.

PWCA leaders facilitated introductions to their membership. Participants were recruited and invited via phone to a church hall near a recycling plant and PWCA headquarters for informed written consenting. Eight trained qualitative researchers, comprising four females and four males with 2 to 15 years' field experience, conducted the FGDs. Authors E.E.B., R.H., J.O., J.A., A.A and G.A.M hold master's degrees in public health or health-related fields; M.A., R.T.A.,

Formatted: Font: 16 pt, Bold

Formatted: Heading 2, Left, Line spacing: single

Formatted: Font: 12 pt

Formatted: Font: 12 pt

Formatted: Font: 12 pt

E.N.A., and G.S. hold bachelor's degrees in information technology or health-related fields. Two FGDs were conducted concurrently in English, but sometimes in Ga, Twi, and Ewe where necessary. E.E.B., R.H., E.N.A., G.A.M., and A.A. facilitated discussions, R.H., E.-E.B. and G.S. took notes alongside audio-recording, whilst J.O., R.T.A., E.N.A., and J.A. supported logistics. M.A. supervised all FGDs, which last approximately two hours and occurred from 26th September to 5th October 2022.

In Kisumu, the Accra topic guide was pre-tested with waste pickers not selected for the main study. Four FGDs were organised with two groups each of waste pickers and intermediaries, and a small group discussion with two of Kisumu's apex traders. Participants were recruited via phone and invited to meetings at a conveniently located community hall and vocational training centre for informed written consenting. Fieldwork took place from 2nd-3rd October 2023, with discussions lasting 58 to 104 minutes. C.O first invited participants to set ground rules, then facilitated discussions. Discussions were predominantly in English, but sometimes in Kiswahili or Luo, depending on participant preference. A.L.O, H.A.A, and B.B.C took notes to supplement audio-recordings. The Kisumu qualitative research team have between 3-15years of qualitative field experience. D.A facilitated logistics, with J.O.O and L.O observing each session. ~~(see S1 Table for research team details).~~

Qualitative dData mManagement, pProcessing and aAnalysis

All audio-recorded FGDs were transcribed verbatim, translated into English if required, and complemented with field notes. Thematic analysis employed both deductive and inductive processes [43]. R.H and E.E.B thematically encoded Accra transcripts using NVivo version 12 [44], with deductive codes representing specific business operations or life cycle stages. Each coder identified themes independently, then met to agree final thematic encoding. Findings

Formatted: Font: 12 pt
Formatted: Font: 12 pt
Formatted: Font: 12 pt
Formatted: Font: 12 pt
Formatted: Font: 12 pt
Formatted: Font: 12 pt
Formatted: Font: 12 pt
Formatted: Font: 12 pt
Formatted: Font: 12 pt
Formatted: Font: Not Bold
Formatted: Font: 12 pt
Formatted: Font: 12 pt
Formatted: Font: 12 pt
Formatted: Font: 12 pt
Formatted: Font: 12 pt
Formatted: Font: 12 pt
Formatted: Font: 12 pt, Strikethrough
Formatted: Font: 12 pt
Formatted: Font: 16 pt, Bold
Formatted: Heading 2, Left, Line spacing: single
Formatted: Font: 12 pt
Formatted: Font: 12 pt
Formatted: Font: 12 pt
Formatted: Font: 12 pt
Formatted: Font: 12 pt
Formatted: Font: 12 pt
Formatted: Font: 12 pt
Formatted: Font: 12 pt

were presented to Accra participants via telephone for member-checking, but there were no participant requests for changes. In Kisumu, C.O transcribed interviews, with RH thematically coding transcripts, but without member checking. L.O and J.O reviewed transcripts against audio recordings. Major themes differentiated opportunities, solutions and challenges, with emerging sub-themes identified inductively. Themes were compared between FGDs, with the most engaging extracts selected to illustrate key themes.

Ethical aApproval

The study was approved by the Faculty of Environmental and Life Sciences Ethical Review Committee, University of Southampton, UK (reference: 55755; approval date 19th August 2020), the Jaramogi Odinga Oginga University of Science and Technology Ethical Review Committee (reference: ERC/23/6/20-4, approval date: 17th August 2020) and the Institutional Review Board of the Noguchi Memorial Institute for Medical Research, University of Ghana (Ref: 003/20-21; approval date: 2nd September 2020).

All study participants provided written informed consent prior to the data collection. Participants were provided with the participant information documents, which was also explained to them in their preferred local languages (Twi, Ewe and Ga for Accra participants, Kiswahili or Luo for Kisumu participants). All study participants were informed about the aim and benefits of the study, as well as their rights as participants. All consenting participants signed (or placed a thumbprint) on an informed consent form before participating in the study. They were assured of confidentiality and that all the information they have provided will be reported with complete anonymity.

Formatted: Font: 12 pt

Formatted: Font: 12 pt

Formatted: Font: 12 pt

Formatted: Font: 16 pt, Bold

Formatted: Heading 2, Left, Line spacing: single

Formatted: Font: 16 pt, Bold

Formatted: Font: 12 pt

Formatted: Font: (Default) +Body (Calibri)

Formatted: Font: (Default) +Body (Calibri)

Inclusivity in global research

Additional information regarding the ethical, cultural, and scientific considerations specific to inclusivity in global research is included in the Supporting Information (Inclusivity-in-global-research-questionnaire_final.docx).

Results

Participant characteristics

In Greater Accra, participating plastic main collectors were the most educated group, predominantly men, and younger- than general collectors,-(Table 1). Plastic sub-collectors were older, largely women, and typically the least educated participants. General waste collectors were all men and generally older.

Table 1. Socio-demographic cCharacteristics of FGD pParticipants (Greater Accra, Ghana)

**Participant characteristics**	**General waste collectors**		**Plastics sub-collectors**		**Plastics main collectors**		**Ttotal**	
**Sex**	**N**	**%**	**N**	**%**	**N**	**%**	**N**	**%**
Male	12	100.0	3	12.5	19	79.2	34	56.7
Female	0	0.0	21	87.5	5	20.81	26	43.3
**Age (years)**								
<20	0	0.0	0	0.0	0	0.0	0	0.0
20-29	1	8.4	2	8.3	4	16.7	7	11.7
30-39	3	25.0	3	12.53	7	29.2	13	21.72
40-49	4	33.3	8	33.3	7	29.2	19	31.72
50+	4	33.3	11	45.86	6	25.0	21	35.0
**Educational Level**								
No formal Education	3	25.0	3	12.5	2	8.3	8	13.3
Primary/Junior High School	4	33.3	15	62.5	5	20.81	24	40.0
Middle/Secondary/ Senior High_-School	5	41.62	6	25.0	16	66.7	27	45.0
Tertiary	0	0.0	0	0.0	1	4.2	1	1.72
**Total**	**12**		**24**		**24**		**60**	

Formatted: Font: 16 pt, Bold
Formatted: Heading 2, Left, Line spacing: single
Formatted: Line spacing: Double
Formatted: Font: (Default) +Body (Calibri)
Formatted: Font: Not Bold, Not Italic
Formatted: Heading 1, Left, Line spacing: single
Formatted: Font: 16 pt, Bold
Formatted: Font: 12 pt
Formatted: Font: 12 pt
Formatted: Font: 12 pt
Formatted Table
Formatted Table
Formatted: Font:
Formatted: Centered
Formatted Table

Relative to Accra, in Kisumu, whilst the gender balance was similar, participants were younger (mostly <40 years old) with more formal education. Most had completed high school, college or university education (Table 2). The two apex traders had university or college education.

Table 2. Socio-demographic characteristics of FGD participants (Kisumu, Kenya)

Participant characteristics	Waste sub-collectors		Intermediate waste traders		Plastic waste apex traders		Total	
**Sex**	**N**	**%**	**N**	**%**	**N**	**%**	N	%
Male	5	38.5	8	66.7	1	50.0	14	51.9
Female	8	61.5	4	33.3	1	50.0	13	48.2
**Age (years)**								
20-29	4	30.8	7	63.6	2	100.0	13	50
30-39	7	53.9	2	18.2	0	0.0	9	34.6
40-49	1	7.7	1	9.1	0	0.0	2	7.7
50+	1	7.7	1	9.1	0	0.0	2	7.7
**Educational Level**								
N/A	0	0.0	1	8.3	0	0.0	1	3.7
Primary School	0	0.0	1	8.3	0	0.0	1	3.7
High School	5	38.5	5	41.7	0	0.0	10	37.0
College	7	53.9	4	33.3	1	50.0	12	44.4
University	1	7.7	1	8.3	1	50.0	3	11.1
**Total**	**13**		**12**		**2**		**27**	

Business entry motivations for plastic versus general waste collectors

Table 3. Themes reflecting opportunities and motivations for waste sector entry, by city and FGD group

Opportunity or motivation	Kisumu SWM groups	Greater Accra SWM groups
Income-earning potential relative to other options	Sub-collectors;<br>Intermediaries	General collectors;<br>Plastic sub-collectors;<br>Plastic main collectors
Contribution to environmental quality	Sub-collectors;<br>Intermediaries;<br>Apex plastic traders	Plastic sub-collectors;<br>Plastic main collectors;<br>General collectors
External support for business start-up, e.g. vehicles, equipment through CSR	Sub-collectors;<br>Intermediaries;<br>Apex plastic traders	General collectors;<br>Plastic sub-collectors;<br>Plastic main collectors
Opportunities from changing waste composition, segregation, and valorisation	Sub-collectors;<br>Intermediaries;<br>Apex plastic traders	General collectors;<br>Plastic main collectors

Formatted: Font: 12 pt

Formatted Table

Formatted Table

Formatted Table

Formatted: Font: 16 pt, Bold

Formatted: Heading 2, Left, Line spacing: single

Formatted Table

Flexible working	S~~s~~ub-collectors	Plastic main collectors
Low capital cost of business entry	S~~s~~ub-collectors	Plastic sub-collectors

For almost all FGDs (Table 3), greater economic opportunity, relative to formal employment or other informal livelihoods, was the main driver for entering the waste sector. For example, an Accra general collector noted:

> *"I have worked for 10 years. I trained as an (name of ~~the work~~occupation)~~auto mechanic~~, but I could not get a job. Someone gave me a tricycle to help me collect waste. Initially, I wanted to load foodstuff at ~~Agbogbloshie~~ [name of market] with the tricycle but then he convinced me to do waste collection. I realized that the business is very profitable because I could buy food and still get some money after I had bought fuel.* ***(R5B, Male, General collector, Accra****)*

Across all groups, many participants were motivated by their operations' environmental and sanitation benefits:

> *"I collect the sachet 'rubbers' [i.e. plastic packaging] because when the wind blows it and it rains, it makes the environment dirty. So, I collect them to make the environment clean."* ***(R5A, Female, Sub-collector, Accra)***

All groups reported receiving some external support from government, NGOs, or corporate social responsibility programmes. For sub-collectors, support comprised occupational health education, but donors sometimes provided other groups with vehicles.

Almost all groups highlighted emerging markets for a wider range of plastics, alongside Accra's established markets for water sachet and PET waste:

> *"When I started there was a rubber known as "Panyin di Panyin" that we were collecting before sachet water was introduced into the system…then the PET bottle.*

Formatted: Font: 12 pt

Formatted: Font: 12 pt

Formatted: Font: 12 pt

Even though there was collection of broken plastic chairs and bowls, they were not common… but now they are rampant” ***(R10B, Male, Main collector, Accra)***

Plastic main collectors attributed the growing range of valorised plastics to greater crusher/pelletiser availability at waste transfer/recycling stations.

General collectors also noted opportunities from more plastics in the domestic mixed waste stream, reflecting changing urban lifestyles:

“Initially, you hardly noticed sachet rubbers and PET bottles but now they are a lot. Even the organic waste has reduced now. Maybe it’s because there are lots of companies producing plastics” ***(R6A, Male, General collector, Accra)***

Group-s~~S~~pecific t~~T~~hemes:

Plastic sub-collectors noted lower start-up costs and security risks, relative to other informal livelihood options:

“The reason why I collect the sachet rubbers is that I used to sell [name of commodity] …, ~~credit (airtime)~~ and I was robbed of all my money. But I have no other means to get money to start over, so someone told me that, if I pick the sachet rubbers, he will buy them, so I got involved. I have been picking the rubbers since 2016 and it has helped me to get money and support my family.” ***(R7A, Male, Sub-collector, Accra)***

Some plastic main collectors also cited flexible working hours as a business entry motivator:

“*I am not working for anybody. I go to work when I choose to. Secondly, I can work for five hours and rest at any time, I can sell the plastic at any time and get my profit. Because it is my own business, I can make time to go for lectures too because am a student”* ***(R6A, Male, Main collector, Accra)***

Formatted: Font: 12 pt

Formatted: Heading 3, Left, Line spacing: single

Formatted: Font: 14 pt, Bold

Formatted: Font: 12 pt

Formatted: Font: 12 pt

Challenges for plastic versus general waste collectors by business operation

Table 4 summarises themes concerning challenges for waste collection business for Accra and Kisumu respectively. Common themes recurring across different business types are presented initially, followed by business-specific challenges.

Table 4. Themes representing challenges for plastic versus general waste collectors by business phase or operation type, identified via FGDs in Greater Accra, Ghana and Kisumu, Kenya

Major theme – business operation / Sub-themes	Kisumu SWM groups affected	Greater Accra SWM groups affected
**Generic challenges**		
Lack of public recognition/respect	-S~~s~~ub-collectors; I~~i~~ntermediaries	General collectors; P~~p~~lastic main collectors
Harassment, intimidation and gendered stigma	-S~~s~~ub-collectors; -W~~w~~aste intermediaries; P~~p~~lastic apex traders	General collectors
**Business entry/establishment**		
Lack of trade association		Plastic sub-collectors
Business registration cost and time investment	-S~~s~~ub-collectors; I~~i~~ntermediaries	Plastic main collectors
Lack of capital	-I~~i~~ntermediaries	
**Waste collection**		
Competition from informal/illegal collectors	-S~~s~~ub-collectors; I~~i~~ntermediaries	General collectors
Competition from similar businesses	-S~~s~~ub-collectors; P~~p~~lastic apex traders	Plastic sub-collectors; P~~p~~lastic main collectors
Occupational health risks from sharp objects, faecal matter in waste	S ~~s~~ub-collectors; I~~i~~ntermediaries	General collectors; P~~p~~lastic main collectors
Occupational health risks from bending to pick / carrying waste		Plastic sub-collectors
Lack of personal protective equipment/tools	S ~~s~~ub-collectors; I~~i~~ntermediaries	General collectors; P~~p~~lastic sub-collectors
Payment delays/defaulting by clients	-sub-collectors; intermediaries	
Lack of household waste separation	Sub-collectors	
**P~~p~~rocessing, storage, & transport**		
Lack of crushing equipment	Intermediaries	General collectors; plastic main collectors
Transportation costs (fuel/vehicle maintenance)	Sub-collectors; I~~i~~ntermediaries	General collectors; -P~~p~~lastic sub-collectors; P~~p~~lastic main collectors
Remoteness of dump sites	Sub-collectors; I~~i~~ntermediaries	General collectors
road restrictions for waste vehicles	Sub-collectors	General collectors
Lack of secure storage with consequent waste theft risk	Intermediaries	Plastic sub-collectors

Formatted: Font: 16 pt, Bold

Formatted: Heading 2, Left, Line spacing: single

Formatted: Font: 12 pt

Formatted Table

**Onward disposal**		
Monopolistic pricing, unfair bargaining; delayed payments	Sub-collectors; I~~i~~ntermediaries; A~~a~~pex plastic traders	General collectors; ~~-~~Plastic main collectors; P~~p~~lastic sub-collectors
Price elasticity from large plastic wate loads from neighbouring countries		Plastic main collectors
Heavier waste in wet season incurring higher charges		General waste collectors
Increased dry season sachet consumption, lowering plastic prices		Plastic sub-collectors; M~~m~~ain collectors
Wet season flooding of recycling plants		Plastic main collectors
Queues when offloading waste at dump sites		General waste collectors

Generic, shared challenges

Most groups faced lack of respect:

"…people despise us because of the work we do, they feel this is dirty work and think we are mad people" **(R2B, Male, Waste Intermediary, Kisumu)**

Many groups described harassment from authorities during waste transportation and drop-off:

> ***"****… the police if they stop you and you have your license and every other document, they will still charge you 300 Ghana cedis (approx. US$20.5 ~~dollars~~). If you refuse to pay, they will threaten to send you to court even though they will not proceed to court. So, as we are helping the nation to collect the waste, the police are stopping us to extort monies from us …"* ***(R3A, Female, Main collector, Accra)***

Several general waste collectors believed that municipal regulations prohibiting collectors' tricycles on Accra's highways exacerbated harassment. However, both Kisumu's sub-collectors and Accra's general collectors recognised their associations' roles in addressing harassment:

Formatted: Font: 14 pt, Bold

Formatted: Heading 3, Left

Formatted: Font: 12 pt

Formatted: Font: 12 pt

"As long as you are a member of the KIWAN they will always come through if you have a problem. For example, if the NEMA guys arrest you they will always call them and work things out. But the KIWAN association also require that we all have our own licenses but before ~~we~~ you get one, they will cover you until when you finally get yours" ***(R2B, Female, Intermediary, Kisumu)***

Kisumu's female collectors were particularly subject to gender-based intimidation and stigmatisation:

"When I first ventured into this, as a woman I faced a lot of challenges. Dealing with male clients is a challenge because of frequent intimidation from men and their spouses. Also, handling our male small pickers who work under us is a challenge because sometimes they come to work drunk and even insult you. There are times they steal our clients' stuff and you are forced to pay... Some men insist that you go inside their houses to collect the money, and some women even think you are having an affair with their husbands" ***(R4B, Female, sub-collector, Kisumu)***

Business ~~e~~Entry ~~c~~Challenges

Formatted: Font: 14 pt, Bold

Formatted: Font: 14 pt, Bold

Formatted: Heading 3, Left

Formatted: Font: 14 pt, Bold

Formatted: Font: Bold

Formatted: Font: 12 pt

Reported business start-up challenges varied by waste enterprise type. Kisumu collectors and Accra's plastic main collectors highlighted complex, costly business registration processes:

"The process is not easy because it requires registration with different bodies which is really hectic like NEMA, and I think NEMA is the biggest challenge. They take long before they approve your business and there are so many evaluation processes. Then there is the county environment department which you must also register with. They must assess if the waste collection point or yard is a health hazard to the population

in the surrounding area. So, I think the process is not easy, complicated and capital intensive" ***(R3A, Female, Waste sub-collector, Kisumu)***

Accra's plastic sub-collectors described how their informal status and lack of collective representation impaired their ability to collectively bargain or mobilise support, relative to plastic main collectors. Kisumu's intermediaries reportedly also lacked start-up capital.

Waste collection challenges

Formatted: Font: 14 pt, Bold

Formatted: Heading 3, Left

Formatted: Font: 12 pt

All groups highlighted increased competition as their main waste collection challenge. For Accra's plastic sub-collectors, competitors comprised new market entrants attracted by greater plastic valorisation:

"Nowadays even at dawn, you will find people calling their children to go out and start collecting the waste. ….There are many people in the business now and it has made it seem like the business is going down. So now I don't get as much as I used to. People now even collect with children when their children are even supposed to be in school" ***(R11A, Female, Sub-collector, Accra)***

In contrast, both cities' general waste collectors were concerned that unregistered collectors illegally dumping mixed waste, undercut their prices:

"…We also have others collecting waste at very low prices therefore hindering business; some collectors agree to collect at as low as 20KSh (US$0.15) while I collect maybe at 50KSh (US$0.39), yet they end up dumping the collected at illegal sites; yet I take waste I have collected all the way to Kasese dump site. They feel that we are expensive, therefore look for the cheaper version. That is why you find that there is a lot of illegal dumping and they get more business with it" ***(R4A, Male, Waste sub-collector, Kisumu)***

Formatted: Font: 12 pt

Formatted: Font: 12 pt

Both cities' groups reported occupational health risks during waste collection from hazardous sharp objects but increasingly also faecal contamination risks from absorbent hygiene products in waste streams:

> *"...When you pick up plastics from them, you can sometimes find diapers, used sanitary pads, faeces wrapped in black polythene, sputum etc. They also place the rubber close to their toilets for you to pick up."* ***(R12A, Male, Main collector, Accra)***

Accra's plastic sub-collectors, mostly elderly women (Table 1), were concerned about treatment costs for musculo-skeletal injuries from picking and carrying waste:

> *"For me, the bending and picking all the time can be injurious to the body...Because no matter how much you can sell it for, if you develop a waist problem, the hospital and treatment will erode all your profits and it wouldn't even be enough for the full treatment"* ***(R2A, Female, Sub-collector, Accra)***

Kisumu's sub-collectors reported spending excessive time separating household waste, given how few households practiced waste separation. They additionally attributed problems with payment delays and defaulting to lack of societal recognition:

> *"… customers don't want to pay and do not value the waste collections business. People still believe it is the city council work, therefore don't see the need for paying"* ***(R6A, Male, Waste Intermediary, Kisumu)***

Waste processing, storage and transportation challenges

For Accra's plastic main collectors and general collectors, the main transport-related challenge was fuel price volatility. Many plastic main collectors and general collectors lacked funds for crushing equipment, exacerbating injury risks when handling waste. A particular challenge for Accra's plastic sub-collectors and Kisumu's waste intermediaries was insecure

Formatted: Font: 12 pt

Formatted: Font: 14 pt, Bold

Formatted: Heading 3, Left

Formatted: Font: 12 pt

plastic waste storage and unpredictable waste collection schedules, heightening risk of collected plastics theft.

Onward waste disposal challenges

All participants were concerned about price negotiation. Accra's main collectors reported an inability to collectively negotiate plastic prices given monopolistic pricing by recycling companies:

> *"…there isn't any government-owned plastic recycling company. Most of the recycling companies that exist are foreigners… And so they have monopoly in the system and arbitrarily determine the prices they will pay for the plastics we collect. If they refuse to pay more for your plastics, there are not many alternatives, so we are forced to accept those low prices"* ***(R11B, Male, Main collector, Accra)***

Low prices at recycling plants reportedly cascaded through waste chains to sub-collectors. Kisumu's apex plastic traders faced competition from Ugandan and Nakuru-based waste brokers from outside the city, who undercut the local plastic waste purchase price. Accra's general waste collectors similarly reported how recent increases in mixed waste fees at dump sites had affected their profit margins.

Seasonal volatility additionally affected Accra's plastic SWM chain. In the dry season, higher water sachet consumption increased sachet plastic waste generation, reducing plastic waste prices. This caused delivery vehicle queues at recycling facilities, delaying processing and payments by recyclers:

> ***"****… In this time of the season (rainy season), you receive payments instantly. Some other times you get the money in the evening after going through a lengthy process. On days where there are lots of rubbers to be processed during the dry season, you may go two to*

Formatted: Font: 14 pt, Bold

Formatted: Heading 3, Left

Formatted: Font: 12 pt

three days or even a week due to load there. In this time of the season (rainy season), there's a shortage in rubbers, hence there is high demand and quick payment.” ***(R5B, Male, Main collector, Accra)***

Conversely, in the cooler wet season, sachet water production dropped in response to lower consumer demand, thereby reducing sachet plastic waste generation and availability for some main collectors. Some main collectors reported how flooding affected recycling plants in the wet season, with plant closures and subsequent clean-up disrupting plastic waste sales. Meanwhile, general collectors also described greater disposal costs for heavier, water-logged mixed waste during the rainy season, given weight-based pricing.

Plastic main collectors reported how large plastic waste shipments from neighbouring countries depressed prices at recycling plants:

> *“…one of the key challenges is the infiltration of foreigners from Burkina Faso and Niger. When these foreigners bring in their plastics, it causes the sellout price to drop for us. Secondly, because they have large loads of plastics, the respect and recognition that is due to us by the recycling company is not given to us. They come with large articulator trucks (40-footer trucks). Once they start coming in our business goes down.”* ***(R10A, Male, Main collector, Accra)***

General waste collectors also encountered year-round delays in offloading waste at the few privately operated disposal sites, sometimes even sleeping overnight in dumpsite queues.

Support Requested by Waste Collectors

Many respondents from different FGDs (Table 5) identified waste worker respect promotion as generating multiple, holistic benefits:

Formatted: Font: 16 pt, Bold

Formatted: Heading 2, Left, Line spacing: single

Formatted: Font: 12 pt

"We need to fight the attitude of the community by sensitization of community so that they value waste and waste collectors, change attitudes towards waste and let waste be everybody's responsibility. It is not a one-person business, it is all of us" ***(R6A, Male, Waste Intermediary, Kisumu)***

Mixed waste collectors requested measures to tackle unregistered collectors illegally dumping waste, particularly government initiatives to:

"…deal with illegal dumping, and government and relevant bodies concerned with matters of waste to recognize registered waste actors who do the right thing. Also motivate small waste pickers who most of the time practice illegal dumping so that they can also do things the right way. That way they will also rise to the corporate world and get licenses" ***(R4A, Male, Waste sub-collector, Kisumu)***

Formatted: Font: 12 pt

Formatted: Font: 12 pt

Plastic waste sub-collectors often requested personal protective equipment (PPE) such as gloves, boots and picking tools to mitigate the occupational health risks of carrying and bending. Many plastic main collectors particularly valued transport provision or support (e.g. relaxation of highway restrictions; fuel subsidies), as it enabled them to increase their coverage areas and customer base.

Plastic main collectors often also requested state-supported plastic recycling companies and general waste collectors state-supported dump sites as measures to address monopolistic pricing. Accra's main collectors highlighted the need for protection from volatility in recycled plastic prices and greater regulation of recyclers to counter unscrupulous price bargaining:

"Government must step into the affairs of this business and help us so that these recycling companies…do not cheat us. The government should get involved and regulate our activities because we don't have an advocate who seeks our welfare. Just imagine sending your plastics that you have bought with money from sub-collectors to

go and sell and it is being ~~weighed~~weighed, and you're prevented from observing figures on the scale…If you want to protest and try to see what is happening, they somehow intentionally reduce your price" ***(R9B, Male, Main collector, Accra)***

Table 5. Solutions to waste enterprise challenges identified by SWM workers in Greater Accra and Kisumu.

Challenge(s)	Solution Theme	Kisumu SWM groups	Greater Accra SWM groups
price negotiation, lack of public recognition, harassment, securing loans, supporting business registration	Formation or strengthening of associations	Intermediaries	
lack of public recognition/respect	Public awareness-raising over benefits of waste enterprises	Intermediaries	General collec
Limited domestic waste separation	Promotion and community sensitisation on domestic waste separation	Sub-collectors; Iintermediaries; Aapex plastic traders	
	Inter-sectoral promotion of organic waste fertiliser	Sub-collectors; Iintermediaries	
	Government policies to boost plastic recycling	Sub-collectors; Aapex plastic traders	Plastic main collectors
Competition from unregulated mixed waste collectors	Policies to address illegal waste dumping	Sub-collectors; Iintermediaries	
occupational health risks	Provision of tools/PPE (e.g. pickers, gloves)	Intermediaries	Plastic sub-collectors
	Medical support/screening	Sub-collectors	Plastic sub-collectors
	Extended producer responsibility for diaper SWM	Sub-collectors	
	state-funded accident insurance		General collec
transport costs & harassment	scrap vehicle permits; lift highway restrictions on waste vehicles; reduced fuel tax for waste collectors	Intermediaries	General collec Plastic main collectors
	transport provision (e.g. tricycles, vans)	Waste intermediaries	Plastic sub-collectors; Pplastic main collectors; Ggeneral collectors
	rationalise waste transfer /dump site locations.	Ssub-collectors	
Price volatility & fair bargaining	Nationalise/regulate recycling companies or dump sites	Ssub-collectors; Iintermediaries	Plastic main collectors;

Formatted: Font: 12 pt
Formatted: Font: (Default) +Body (Calibri)
Formatted Table
Formatted: Font: (Default) +Body (Calibri)
Formatted: Font: (Default) +Body (Calibri)
Formatted: Font: (Default) +Body (Calibri)
Formatted: Font: (Default) +Body (Calibri)
Formatted: Font: (Default) +Body (Calibri)
Formatted: Font: (Default) +Body (Calibri), 11 pt
Formatted: Font: (Default) +Body (Calibri)
Formatted: Font: (Default) +Body (Calibri)
Formatted: Font: (Default) +Body (Calibri)
Formatted: Font: (Default) +Body (Calibri)
Formatted: Font: (Default) +Body (Calibri)
Formatted: Font: (Default) +Body (Calibri)
Formatted: Font: (Default) +Body (Calibri)
Formatted: Font: (Default) +Body (Calibri)
Formatted: Font: (Default) +Body (Calibri), 11 pt
Formatted: Font: (Default) +Body (Calibri)
Formatted: Font: (Default) +Body (Calibri), 11 pt
Formatted: Font: (Default) +Body (Calibri)
Formatted: Font: (Default) +Body (Calibri)
Formatted: Font: (Default) +Body (Calibri)
Formatted: Font: (Default) +Body (Calibri), 11 pt
Formatted: Font: (Default) +Body (Calibri)
Formatted: Font: (Default) +Body (Calibri)
Formatted: Font: (Default) +Body (Calibri)
Formatted: Font: (Default) +Body (Calibri), 11 pt
Formatted: Font: (Default) +Body (Calibri)
Formatted: Font: (Default) +Body (Calibri)
Formatted: Font: (Default) +Body (Calibri)
Formatted: Font: (Default) +Body (Calibri)
Formatted: Font: (Default) +Body (Calibri)

			-General collec
	regulate/ban waste imports from neighbouring countries		Plastic main collectors
	Price regulation	-~~S~~sub-collectors; ~~I~~intermediaries; ~~A~~apex plastic traders	-Plastic main collectors; -~~G~~general collectors

Discussion

Few studies have examined how formal-informal SWM chains in LMICs are evolving to handle separated plastic waste. Our study highlights how specialist plastic waste enterprises face distinct challenges not faced by mixed waste enterprises, in part reflecting workforce socio-demographics. Firstly, relative to mixed waste collectors, Greater Accra's specialist plastic enterprises are more affected by volatility in plastic waste generation and prices. Plastic SWM chains are susceptible to international price fluctuations for recycled plastics. For example, in the Covid-19 pandemic, lower oil prices reduced virgin plastic prices, thereby depressing international recycled plastic prices [45]. Ecuadorian plastic waste-pickers similarly reported price volatility as their greatest challenge [46]. However, our study highlights how local factors within Accra further exacerbate recovered plastic price volatility. One local driver of price volatility is seasonal variation in water sachet consumption and thus sachet plastic waste generation by Accra's extensive sachet water industry. Accra's recyclers are known to increasingly use imported plastics [47]. We also find periodic bulk importation of waste plastics into Accra from neighbouring countries increases throughput at recycling plants, but sporadically depresses prices for local waste collectors. Accra's status as a regional plastic recycling hub thus creates price volatility for local plastic waste businesses.

Formatted: Font: (Default) +Body (Calibri)
Formatted: Font: (Default) +Body (Calibri), 11 pt
Formatted: Font: (Default) +Body (Calibri)
Formatted: Font: (Default) +Body (Calibri)
Formatted: Font: (Default) +Body (Calibri)
Formatted: Font: (Default) +Body (Calibri)
Formatted: Font: (Default) +Body (Calibri)
Formatted: Font: (Default) +Body (Calibri)
Formatted: Font: (Default) +Body (Calibri)
Formatted: Font: 18 pt, Bold
Formatted: Heading 1
Formatted: Heading 1, Left
Formatted: Font: 12 pt
Formatted: Font: 12 pt
Formatted: Font: 12 pt
Formatted: Font: 12 pt
Formatted: Font: 12 pt
Formatted: Font: 12 pt
Formatted: Font: 12 pt

A further challenge specific to plastic waste collectors is secure storage of segregated plastic waste. Informal waste collectors face well-documented difficulties in storing and processing collected waste given over-crowding, lack of space and formal planning in slums [10]. However, insecurity in slums, coupled with the value of aggregated plastic waste, further exacerbates this working space issue for informal plastics collectors, making them vulnerable to waste theft. Whilst other studies have reported African waste collectors being susceptible to theft of personal positions when working in crime-prone neighbourhoods [48] and noted equipment theft (particularly of waste bins) [49], theft of valuable separated waste has seldom previously been reported. Conversely, mixed waste collectors are subject to price undercutting by competitors who illegally dump mixed waste. In contrast, given its value, there is no financial incentive to dump plastic waste.

Furthermore, our study shows that Greater Accra's SWM workforce is socio-demographically differentiated. Informal plastic waste sub-collectors are mostly older women, sometimes accompanied by children. This conforms with the well-established U-shaped demographic curve of informal sector participation [50], where the elderly and youth predominate. Consequently, these elderly plastic sub-collectors fear occupational health risks, particularly musculo-skeletal injuries from picking and carrying waste. Kisumu's FGD participants were well educated and generally younger than those in Greater Accra. Many of Kisumu's current waste enterprises emerged from youth groups [39], so the city's youthful waste collector profile likely reflects these origins. The comparatively high educational attainment among Kisumu's waste collectors may reflect rapid expansion of Kenya's higher education system [51] in the early 2000s and subsequent rise in graduate unemployment [52]. It suggests that Kisumu's informal waste sector is less susceptible to some commonly cited barriers to formalisation, namely informal workers' lack of education and skills [53].

All FGDs in both cities reported shared challenges widely reported elsewhere. Notably, participants highlighted lack of societal recognition for their work (Table 4) despite its environmental contribution, echoing many other waste-picker studies [11]. Similarly, harassment by authorities, reported by most FGDs, has been widely reported by informal waste sector workers across SSA and Latin America [54]. Confirming trends in plastic imports to Africa [55], both cities' FGD participants highlighted increasing plastics in MSW composition. Participants also reported increasingly encountering hazardous products within the waste stream, reflecting informal waste-picker concerns over occupational health in many other cities [11]. FGDs reported growing faecal contamination risks from absorbent hygiene products (particularly disposable diapers), confirming recent studies reporting increased diaper waste in urban SSA . Participant motivations for entering the waste sector were broadly consistent between both cities (Table 3), aligning with studies elsewhere. For example, Ecuadorian informal plastic waste-pickers cited flexible working and income generation potential within the sector as key drivers for their livelihood choices [46]. Kisumu participants highlighted need for public awareness-raising concerning waste separation, whereas Greater Accra participants had no such concerns given the practice was already widespread (Table 5). Instead, Accra participants described how plastics recycled expanded from sachet packaging initially to subsequently include more varied plastics. Contrary to most countries where rigid plastics are recycled more than flexible plastics [58], plastic SWM in Accra thus initially focussed on flexible sachet packaging, triggered by the need to handle the waste stream from Ghana's extensive sachet water industry [29]. Thus, SWM transitions in LMIC cities towards incorporating plastics may follow different trajectories to high income countries.

In Accra, plastic waste sub-collectors articulated their need for a representative association, similar to associations representing general waste collectors, the ESPA and the PWCA representing plastics main collectors. FGD participants foresaw benefits from such an association's ability to mediate disputes with authorities (Table 5). Kisumu participants similarly highlighted KIWAN's value as an association. Literature supports these participant-led priorities: many initiatives to support informal waste collectors entail partnerships with waste collector associations and such associations are considered critical to informal collectors gaining recognition [10].

Our findings support proposals to integrate waste pickers as partners in SWM systems to build just, inclusive, and liveable cities [59]. Despite policy and programmatic efforts at informal sector SWM integration in several SSA cities including in Kenya [13], other SSA nations, including Ghana, are yet to develop such policies. Our findings suggest that formalisation initiatives should support fairer price negotiation and reduce price volatility for informal plastic waste-pickers. For example, a Nairobi-based initiative established a local network of recycling company agents, who bought plastics directly from waste-pickers, by-passing intermediate traders [21]. Alongside savings from purchasing directly from pickers, establishing trust through shared meals with waste-pickers increased waste quality, enabling agents to buy waste for a fixed, year-round flat fee. However, context-sensitive programmatic formulation is critical: although policy-based formalisation and integration of waste-pickers can potentially improve waste-picker livelihoods, such policies vary by LMIC and sometimes fail to provide these benefits [11].

Our findings also identify group-specific forms of support for informal collectors that could be targeted through extended producer responsibility programmes. These include secure storage facilities for segregated waste, crushing equipment, and transportation for plastic

main collectors. Although Accra's plastic sub-collectors proposed free PPE provision to address musculo-skeletal injury and contamination risks from hazardous waste, some studies have found that impoverished waste-pickers sell these items to supplement their income[21], undermining PPE's intended benefits. Thus, careful PPE programmatic design is needed to deliver intended benefits, with consideration of other occupational health measures such as medical check-ups, daycare for workers' children, or awareness-raising initiatives.

Our study's limitations include partial sampling of SWM chain actors, creating an incomplete perspective on related challenges. We did not sample dumpsite scavengers or representatives of large end-of-chain formal businesses, such as waste transfer station or recycling plant managers. Child waste pickers were excluded from our study because of informed consenting difficulties. Whilst we avoided the purposive or convenience non-representative sampling strategies commonly used when studying informal SWM chains, our sampling frame was reliant on membership registers of waste collector associations and Accra's municipal authorities. These registers are likely to omit small-scale, more informal and part-time waste sector workers. Ambiguity in our enterprise type definitions for planning FGD recruitment may also have affected group composition and thereby dynamics.

Our findings indicate a need for further evaluation studies assessing the effectiveness of programmes to protect waste sub-collector occupational health and assessing measures to protect their livelihoods from price fluctuations or unfair bargaining practices. Furthermore, reported volatility in plastic waste flows and prices suggests that the many cross-sectional studies quantifying these properties of informal SWM chains, risk mis-representing material flows and waste values. We therefore recommend either longitudinal studies of SWM systems, mixed methods studies to capture temporal variability, or, at minimum, explicit consideration of seasonal and fluctuating economic conditions' impact on cross-sectional

Formatted: Font: 12 pt

Formatted: Font: 12 pt

Formatted: Font: 12 pt

Formatted: Font: 12 pt

Formatted: Font: 12 pt

Formatted: Font: 12 pt

study findings concerning waste prices and material flows. Our participants described recent expansion in the range of material types they recovered. This highlights a need to evaluate strategies that incentivise and accelerate informal sector recovery of even more material types. These strategies include waste valorisation, recycle material quality (by encouraging door-to-door collection over dumpsite scavenging and separating hazardous waste components), and minimising waste pickers' search/collection effort (e.g. using ICT) [22].

Conclusion

As African formal-informal SWM chains evolve to handle plastic waste, our study identifies several challenges specific to plastic waste enterprises. Firstly, particularly in Greater Accra, plastic waste enterprises struggle with greater price volatility, with plastic waste prices affected by bulk plastic waste shipments arriving from neighbouring countries, seasonally variable plastic waste generation (particularly from sachet water), and international oil price variations. Secondly, mixed waste collectors in both cities are subject to price under-cutting from unregistered collectors who dump waste illegally, thereby avoiding disposal fees. In contrast, plastic collectors are inherently less vulnerable to such competition, given the value of plastics. Finally, plastics collectors have greater need for secure waste storage than mixed waste collectors, given separated plastics' value. Furthermore, waste worker demographics interact with urban socio-economic conditions to create context-specific challenges. Greater Accra's plastic sub-collectors are mostly older women, who are thus at risk of musculo-skeletal occupational health conditions when picking and collecting waste. Meanwhile, many of Kisumu's female waste collectors reported gender-aggravated stigmatisation and discrimination.

Our study also confirms well-established shared priorities for informal waste collectors, including the need for greater societal recognition, alongside forming and strengthening

Formatted: Font: 12 pt

Formatted: Font: 12 pt

Formatted: Font: 18 pt, Bold

Formatted: Heading 1

Formatted: Font: 12 pt

waste collector associations. Our participants also highlight increased hazardous faecal matter (e.g. disposable diapers, defecation in bags) within the waste stream as a growing occupational health risk. Collectively, these findings and FGD participants themselves highlight priorities to support informal waste collectors, notably fair bargaining mechanisms, collector association support, safer diaper SWM systems, and occupational health programmes.

Acknowledgements

Formatted: Font: 18 pt, Bold

Formatted: Heading 1

Formatted: Font: 12 pt

We are grateful to all stakeholders, including leadership and collectors within the waste management sectors in Kisumu, Kenya and Greater Accra, Ghana who devoted their time to share their experiences with us for the study. We also wish to thank all field staff for their tremendous work during data collection, and our project steering committees in Greater Accra and Kisumu for their feedback on our findings. We are particularly grateful to Caro Omom for facilitation and transcribing of FGDs in Kisumu.

~~Supplementary Information Captions~~

Formatted: Font: 18 pt, Bold

Formatted: Font: 18 pt, Bold

Formatted: Heading 1

~~**S1 Table. Research team characteristics in Kisumu, Kenya.**~~

References

Formatted: Font: 18 pt, Bold

Formatted: Heading 1

1. Kaza S, Yao L, Bhada-Tata P, Van Woerden F. What a waste 2.0: a global snapshot of solid waste management to 2050. Washington D.C.: 2018.
2. United Nations. 11 Sustainable Cities and Communities: Sustainable Development Goals Extended Report 2022. New York: 2023.
3. Tong YD, Huynh TDX, Khong TD. Understanding the role of informal sector for sustainable development of municipal solid waste management system: A case study in Vietnam. Waste Management. 2021;124:118-27. doi: https://doi.org/10.1016/j.wasman.2021.01.033.
4. UNEP. Africa Waste Management Outlook. Nairobi, Kenya: 2018.

5. Jambeck JR, Geyer R, Wilcox C, Siegler TR, Perryman M, Andrady A, et al. Plastic waste inputs from land into the ocean. Science. 2015;347(6223):768-71. doi: 10.1126/science.1260352. PubMed PMID: WOS:000349221300047.
6. Steinmetz Z, Wollmann C, Schaefer M, Buchmann C, David J, Tröger J, et al. Plastic mulching in agriculture. Trading short-term agronomic benefits for long-term soil degradation? Science of The Total Environment. 2016;550:690-705. doi: https://doi.org/10.1016/j.scitotenv.2016.01.153.
7. Conkle JL, Báez Del Valle CD, Turner JW. Are We Underestimating Microplastic Contamination in Aquatic Environments? Environmental Management. 2018;61(1):1-8. doi: 10.1007/s00267-017-0947-8.
8. Lebreton L, Andrady A. Future scenarios of global plastic waste generation and disposal. Palgrave Communications. 2019;5(1):6. doi: 10.1057/s41599-018-0212-7.
9. Rafey A, Siddiqui FZ. A review of plastic waste management in India – challenges and opportunities. International Journal of Environmental Analytical Chemistry. 2023;103(16):3971-87. doi: 10.1080/03067319.2021.1917560.
10. Dias SM. Waste pickers and cities. Environment and Urbanization. 2016;28(2):375-90. doi: 10.1177/0956247816657302.
11. Morais J, Corder G, Golev A, Lawson L, Ali S. Global review of human waste-picking and its contribution to poverty alleviation and a circular economy. Environmental Research Letters. 2022;17(6):063002. doi: 10.1088/1748-9326/ac6b49.
12. Paul JG, Arce-Jaque J, Ravena N, Villamor SP. Integration of the informal sector into municipal solid waste management in the Philippines – What does it need? Waste Management. 2012;32(11):2018-28. doi: https://doi.org/10.1016/j.wasman.2012.05.026.
13. Oduro-Appiah K, Scheinberg A, Afful A, de Vries N. The contribution of participatory engagement strategies to reliable data gathering and inclusive policies in developing countries: Municipal solid waste management data in the Greater Accra Metropolitan Area of Ghana. African Journal of Science, Technology, Innovation and Development. 2021;13(6):735-46. doi: 10.1080/20421338.2020.1797267.
14. Post J, Broekema J, Obirih-Opareh N. Trial and Error in Privatisation: Experiences in Urban Solid Waste Collection in Accra (Ghana) and Hyderabad (India). Urban Studies. 2003;40(4):835-52. doi: 10.1080/0042098032000065326.
15. Kassim SM, Ali M. Solid waste collection by the private sector: Households' perspective—Findings from a study in Dar es Salaam city, Tanzania. Habitat International. 2006;30(4):769-80. doi: https://doi.org/10.1016/j.habitatint.2005.09.003.
16. Oduro-Kwarteng S, van Dijk MP. The effect of increased private sector involvement in solid waste collection in five cities in Ghana. Waste Management & Research. 2013;31(10_suppl):81-92. doi: 10.1177/0734242X13496305.
17. .
20. Del Pilar Moreno-SÁNchez R, Maldonado JH. Surviving from garbage: the role of informal waste-pickers in a dynamic model of solid-waste management in developing countries. Environment and Development Economics. 2006;11(3):371-91.
21. Gall M, Wiener M, Chagas de Oliveira C, Lang RW, Hansen EG. Building a circular plastics economy with informal waste pickers: Recyclate quality, business model, and societal impacts. Resources, Conservation and Recycling. 2020;156:104685. doi: https://doi.org/10.1016/j.resconrec.2020.104685.
22. Velis CA, Hardesty BD, Cottom JW, Wilcox C. Enabling the informal recycling sector to prevent plastic pollution and deliver an inclusive circular economy. Environmental Science & Policy. 2022;138:20-5. doi: https://doi.org/10.1016/j.envsci.2022.09.008.
23. van Leeuwen J, Surya IRF. Network power and exclusion of informal waste pickers when plastic flows change: A case study of community waste banks in Klaten Municipality in Indonesia. Marine Policy. 2024;167:106285. doi: https://doi.org/10.1016/j.marpol.2024.106285.

24. Cresswell JW, Clark VLP. Designing and conducting mixed methods research. London: Sage Publications; 2017.
25. Ghana Statistical Services. Ghana 2021 Population and Housing Census: Population of Regions and Districts. Accra: 2021.
26. Miezah K, Obiri-Danso K, Kádár Z, Fei-Baffoe B, Mensah MY. Municipal solid waste characterization and quantification as a measure towards effective waste management in Ghana. Waste Management. 2015;46:15-27. doi: https://doi.org/10.1016/j.wasman.2015.09.009.
27. Ghana Statistical Service. Ghana 2021 Population and Housing Census General Report Vol. 3M: Water and Sanitation. Accra: 2022.
28. Oduro-Appiah K, Scheinberg A, Mensah A, Afful A, Boadu HK, de Vries N. Assessment of the municipal solid waste management system in Accra, Ghana: A 'Wasteaware' benchmark indicator approach. Waste Management & Research. 2017;35(11):1149-58. doi: 10.1177/0734242X17727066.
29. Stoler J. From curiosity to commodity: a review of the evolution of sachet drinking water in West Africa. WIREs Water. 2017;4(3):e1206. doi: https://doi.org/10.1002/wat2.1206.

~~Supplementary Information Captions~~

Formatted: Strikethrough

~~S1 Table. Research team characteristics in Kisumu, Kenya.~~

RESPONSE TO REVIEWERS' COMMENTS: Challenges and opportunities for plastic versus mixed waste enterprises in Greater Accra and Kisumu: a qualitative study (ref: PONE-D-25-23611)

Thank you for the constructive feedback provided on our manuscript. Below are comments made by reviewers and the editorial team, as well as point-by-point responses indicating how and where these have been addressed throughout the manuscript.

S/N	REVIEW COMMENT	RESPONSES
	**Editorial Comments**	
1.	Please ensure that your manuscript meets PLOS ONE's style requirements, including those for file naming. The PLOS ONE style templates can be found at…	The manuscript has been formatted in accordance with PLOS ONE's manuscript style requirements.
	Please include a complete copy of PLOS' questionnaire on inclusivity in global research in your revised manuscript. Our policy for research in this area aims to improve transparency in the reporting of research performed outside of researchers' own country or community. The policy applies to researchers who have travelled to a different country to conduct research, research with Indigenous populations or their lands, and research on cultural artefacts. The questionnaire can also be requested at the journal's discretion for any other submissions, even if these conditions are not met. Please find more information on the policy and a link to download a blank copy of the questionnaire here: https://journals.plos.org/plosone/s/best-practices-in-research-reporting. Please upload a completed version of your questionnaire as Supporting Information when you resubmit your manuscript.	We have completed the questionnaire on inclusivity in global research and uploaded it as Supporting Information as requested.
	Thank you for stating in your Funding Statement:<br>[The work was undertaken through the 'Expanding safe water and waste management service access to off-grid populations in Africa' project, funded through UKRI Collective award via the Global Challenges Research Fund (ref: ES/T008121/1). The support of the UK Economic and Social Research Council (ESRC) is gratefully acknowledged.].<br>Please provide an amended statement that declares *all* the funding or sources of support (whether external or internal to your organization) received during this study, as detailed online in our guide for authors at http://journals.plos.org/plosone/s/submit-now. Please also include the statement "There was no additional external funding received for this study." in your updated Funding Statement.	As requested, we provide an updated funding including the explicit statement about no further external funding via our latest covering letter.

	Please include your amended Funding Statement within your cover letter. We will change the online submission form on your behalf.	
	Thank you for stating the following in the Competing Interests section:<br>[MD is academic representative on Ghana's National Technical Working Group on Sanitation, which informs solid waste management policy. The other authors declare no competing interests.].<br>Please confirm that this does not alter your adherence to all PLOS ONE policies on sharing data and materials, by including the following statement: "This does not alter our adherence to PLOS ONE policies on sharing data and materials." (as detailed online in our guide for authors http://journals.plos.org/plosone/s/competing-interests). If there are restrictions on sharing of data and/or materials, please state these. Please note that we cannot proceed with consideration of your article until this information has been declared.<br>Please include your updated Competing Interests statement in your cover letter; we will change the online submission form on your behalf.	The updated competing interest has been included in the cover letter.
	We note that your paper includes detailed descriptions of individual participants. As per the PLOS ONE policy (http://journals.plos.org/plosone/s/submission-guidelines#loc-human-subjects-research) on papers that include identifying, or potentially identifying, information, the individual(s) or parent(s)/guardian(s) must be informed of the terms of the PLOS open-access (CC-BY) license and provide specific permission for publication of these details under the terms of this license. Please download the Consent Form for Publication in a PLOS Journal (http://journals.plos.org/plosone/s/file?id=8ce6/plos-consent-form-english.pdf). The signed consent form should not be submitted with the manuscript, but should be securely filed in the individual's case notes. Please amend the methods section and ethics statement of the manuscript to explicitly state that the patient/participant has provided consent for publication: "The individual in this manuscript has given written informed consent (as outlined in PLOS consent form) to publish these case details".	On reviewing our manuscript, we believe that this comment refers particularly to the supplementary materials (S1 Table) of our original submission, as per the comment below. This table actually described the demographic and educational characteristics of the Kenyan research team members, NOT OUR PARTICIPANTS. We included this table to provide detail on the backgrounds and nature of those conducting this qualitative research study, thereby meeting items on the COREQ checklist for reporting qualitative research (https://www.equator-network.org/reporting-guidelines/coreq/). Nonetheless, we accept that there is a data protection issue around reporting the personal characteristics of Kenyan project team members, so we have now deleted it without compromising COREQ compliance. We have also

		reviewed all quotations in the main manuscript, further redacting any personal details (e.g. of specific sites or occupations) that could potentially lead to inadvertent disclosure of participant identities. If we have misunderstood the editorial concerns, please let us know.
	We note that there is identifying data in the Supporting Information file <S1 Table>. Due to the inclusion of these potentially identifying data, we have removed this file from your file inventory. Prior to sharing human research participant data, authors should consult with an ethics committee to ensure data are shared in accordance with participant consent and all applicable local laws.<br>Data sharing should never compromise participant privacy. It is therefore not appropriate to publicly share personally identifiable data on human research participants. The following are examples of data that should not be shared:<br>-Name, initials, physical address<br>-Ages more specific than whole numbers<br>-Internet protocol (IP) address<br>-Specific dates (birth dates, death dates, examination dates, etc.)<br>-Contact information such as phone number or email address<br>-Location data<br>-ID numbers that seem specific (long numbers, include initials, titled "Hospital ID") rather than random (small numbers in numerical order<br>Data that are not directly identifying may also be inappropriate to share, as in combination they can become identifying. For example, data collected from a small group of participants, vulnerable populations, or private groups should not be shared if they involve indirect identifiers (such as sex, ethnicity, location, etc.) that may risk the identification of study participants.<br>Additional guidance on preparing raw data for publication can be found in our Data Policy (https://journals.plos.org/plosone/s/data-availability#loc-human-research-participant-data-and-other-sensitive-data) and in the following article: http://www.bmj.com/content/340/bmj.c181.long.<br>Please remove or anonymize all personal information (<specific identifying information in file to be removed>), ensure that the data shared are in accordance with participant consent, and re-upload a fully anonymized data set. Please note that spreadsheet columns with personal information must be removed and not hidden as all hidden columns will appear in the published file.	As noted above, supplementary file S1 Table, which actually described the characteristics of Kenyan project research team members but NOT RESEARCH PARTICIPANTS, has now been deleted as supporting information.
	**Reviewer #1**	

1.	**Abstract:**	
•	The role of formal-informal SWM collectors towards plastic waste collection is missing. This should be highlighted (in line 19) to establish a linkage and a stronger basis for the study. A more logical order will be: Who handles plastics? What challenges are they facing?	This has been addressed in line 19 of the Abstract section (Page 2)
•	How many participants participated in each focus group?	This has been included in the methods section of the abstract and can be found in lines 26 and 27 (i.e. 6-12 participants per FGD (n=87))
2.	**Introduction**	
•	The authors have provided an extensive background to the study and supported this with adequate literature. However, the sections look disjointed and verbose. The introduction could be shortened and more direct. It is recommended that the sections are re-ordered to ensure a chronology of the study's background. This chronology could be interspersed or adopted: What is the global SWM (as already established in Lines 39 to 49), who are the actors of SWM, what are their specific roles in SWM, what is their socio-economic motivation, what research gaps remain in their specific role vis-à-vis plastic waste recycling?	The introduction has been re-ordered for a better flow, with some content abridged to create a more concise background to the study.
3.	**Materials and Methods**	
•	**study sites**<br>How much plastic waste/recyclables is generated in Ghana?	This information has now been included under the study sites (page 4).
•	Line 123,…and processed waste. Introduce "and"	This has been introduced (line 128, page 5)
•	Authors may consider including a map of the study areas	We have inserted a new Figure 1 in our Materials and Methods section, showing maps of the two study areas, drawing on the CC BY license of geoboundaries.
•	The authors may consider including information on the number of registered members of the SWM associations, the number of waste management companies, the number of recycling companies, among others.	In our revised manuscript, we now include some additional statistics on numbers of recycling companies and example membership figures for a SWM association.
5.	Results and Discussion	
•	Line 290 to 29 Confirm if the requested amount is GHS30 or 300	The requested amount is indeed 300 cedis.
•	The dollar equivalent should be highlighted in brackets.	The dollar equivalent has been added (page 14) and for two other quotations, we have similarly added dollar equivalent values for KSh amounts.

•	Line 301 to 302 Modify the sentence…but before we you get…(we and you appear together)	This has been modified under the results section (Pages 14-15)
•	Under Table 5, under Kisumu SWM groups line 2, intermediaries should be formatted in terms of font and font size	This table has been formatted accordingly
	Maintain consistency in font size and type (sentence case, capitalisation) across all tables	All tables have been formatted to have consistency in font size and type as well as capitalization.
•	Line 458 to 462 How does this finding compare with other studies in the region or globally	We have edited the text to address this comment. We now note that other studies have reported theft of personal positions (in insecure neighbourhoods) and of waste collection equipment, but not the specific theft of valuable separated plastic waste.
•	Authors should examine the influence of security agencies on collectors, particularly regarding the collection of illegal monies, intimidation, and its effects on (de)motivation for waste collectors.	We now return to the theme of harassment by authorities in paragraph 4 of our revised Discussion, noting that this is a recurrent theme in literature on informal waste collectors.
•	Line 535 provide a space between references and "chains"	This change has been effected (page 25)
	**Reviewer # 2**	
6.	First, I recommend using consistent precision across all tables. Currently, some columns have precision to one digit while others do not.	All tables have now been formatted accordingly, with all percentages given to 1 decimal place.
7.	please double-check the initials from lines 182 to 187. I am not sure if these are the initials of the authors or not. If they are, please ensure consistency.	We have amended the text here to make it clear that author initials are listed at this point, checking formatting of initials for consistency.
8.	Lastly, Table 5 has a different font style compared to all the other tables. Please standardize the font style for uniformity.	Table 5 has been formatted to have consistent font size and style with all the other tables.
