# Supplementary material for: Challenges and opportunities for plastic versus mixed waste enterprises in Greater Accra and Kisumu: A qualitative study
Source: PLoS One. 2026 Jun 8;21(6):e0350670. doi: 10.1371/journal.pone.0350670 (PMC13245779; doi:10.1371/journal.pone.0350670)
Supplement: Attachment — Submitted filename: ResponseReviewers_August2025.docx [file pone.0350670.s003.docx]

**RESPONSE TO REVIEWERS’ COMMENTS: Challenges and opportunities for plastic versus mixed waste enterprises in Greater Accra and Kisumu: a qualitative study (ref: PONE-D-25-23611)**

Thank you for the constructive feedback provided on our manuscript. Below are comments made by reviewers and the editorial team, as well as point-by-point responses indicating how and where these have been addressed throughout the manuscript.

| **S/N** | **REVIEW COMMENT** | **RESPONSES** |
| --- | --- | --- |
|  | **Editorial Comments** |  |
| 1. | Please ensure that your manuscript meets PLOS ONE's style requirements, including those for file naming. The PLOS ONE style templates can be found at… | The manuscript has been formatted in accordance with PLOS ONE’s manuscript style requirements. |
|  | Please include a complete copy of PLOS’ questionnaire on inclusivity in global research in your revised manuscript. Our policy for research in this area aims to improve transparency in the reporting of research performed outside of researchers’ own country or community. The policy applies to researchers who have travelled to a different country to conduct research, research with Indigenous populations or their lands, and research on cultural artefacts. The questionnaire can also be requested at the journal’s discretion for any other submissions, even if these conditions are not met.  Please find more information on the policy and a link to download a blank copy of the questionnaire here: <https://journals.plos.org/plosone/s/best-practices-in-research-reporting>. Please upload a completed version of your questionnaire as Supporting Information when you resubmit your manuscript. | We have completed the questionnaire on inclusivity in global research and uploaded it as Supporting Information as requested. |
|  | Thank you for stating in your Funding Statement:  [The work was undertaken through the ‘Expanding safe water and waste management service access to off-grid populations in Africa’ project, funded through UKRI Collective award via the Global Challenges Research Fund (ref: ES/T008121/1). The support of the UK Economic and Social Research Council (ESRC) is gratefully acknowledged.].  Please provide an amended statement that declares *all* the funding or sources of support (whether external or internal to your organization) received during this study, as detailed online in our guide for authors at <http://journals.plos.org/plosone/s/submit-now>.  Please also include the statement “There was no additional external funding received for this study.” in your updated Funding Statement.  Please include your amended Funding Statement within your cover letter. We will change the online submission form on your behalf. | As requested, we provide an updated funding including the explicit statement about no further external funding via our latest covering letter. |
|  | Thank you for stating the following in the Competing Interests section:  [MD is academic representative on Ghana’s National Technical Working Group on Sanitation, which informs solid waste management policy. The other authors declare no competing interests.].  Please confirm that this does not alter your adherence to all PLOS ONE policies on sharing data and materials, by including the following statement: "This does not alter our adherence to PLOS ONE policies on sharing data and materials.” (as detailed online in our guide for authors <http://journals.plos.org/plosone/s/competing-interests>).  If there are restrictions on sharing of data and/or materials, please state these. Please note that we cannot proceed with consideration of your article until this information has been declared.  Please include your updated Competing Interests statement in your cover letter; we will change the online submission form on your behalf. | The updated competing interest has been included in the cover letter. |
|  | We note that your paper includes detailed descriptions of individual participants. As per the PLOS ONE policy (<http://journals.plos.org/plosone/s/submission-guidelines#loc-human-subjects-research>) on papers that include identifying, or potentially identifying, information, the individual(s) or parent(s)/guardian(s) must be informed of the terms of the PLOS open-access (CC-BY) license and provide specific permission for publication of these details under the terms of this license. Please download the Consent Form for Publication in a PLOS Journal (<http://journals.plos.org/plosone/s/file?id=8ce6/plos-consent-form-english.pdf>). The signed consent form should not be submitted with the manuscript, but should be securely filed in the individual's case notes. Please amend the methods section and ethics statement of the manuscript to explicitly state that the patient/participant has provided consent for publication: “The individual in this manuscript has given written informed consent (as outlined in PLOS consent form) to publish these case details”. | On reviewing our manuscript, we believe that this comment refers particularly to the supplementary materials (S1 Table) of our original submission, as per the comment below. This table actually described the demographic and educational characteristics of the Kenyan research team members, NOT OUR PARTICIPANTS. We included this table to provide detail on the backgrounds and nature of those conducting this qualitative research study, thereby meeting items on the COREQ checklist for reporting qualitative research (<https://www.equator-network.org/reporting-guidelines/coreq/>). Nonetheless, we accept that there is a data protection issue around reporting the personal characteristics of Kenyan project team members, so we have now deleted it without compromising COREQ compliance. We have also reviewed all quotations in the main manuscript, further redacting any personal details (e.g. of specific sites or occupations) that could potentially lead to inadvertent disclosure of participant identities. If we have misunderstood the editorial concerns, please let us know. |
|  | We note that there is identifying data in the Supporting Information file <S1 Table>. Due to the inclusion of these potentially identifying data, we have removed this file from your file inventory. Prior to sharing human research participant data, authors should consult with an ethics committee to ensure data are shared in accordance with participant consent and all applicable local laws.  Data sharing should never compromise participant privacy. It is therefore not appropriate to publicly share personally identifiable data on human research participants. The following are examples of data that should not be shared:  -Name, initials, physical address -Ages more specific than whole numbers -Internet protocol (IP) address -Specific dates (birth dates, death dates, examination dates, etc.) -Contact information such as phone number or email address -Location data -ID numbers that seem specific (long numbers, include initials, titled “Hospital ID”) rather than random (small numbers in numerical order  Data that are not directly identifying may also be inappropriate to share, as in combination they can become identifying. For example, data collected from a small group of participants, vulnerable populations, or private groups should not be shared if they involve indirect identifiers (such as sex, ethnicity, location, etc.) that may risk the identification of study participants.  Additional guidance on preparing raw data for publication can be found in our Data Policy (<https://journals.plos.org/plosone/s/data-availability#loc-human-research-participant-data-and-other-sensitive-data>) and in the following article: <http://www.bmj.com/content/340/bmj.c181.long>.  Please remove or anonymize all personal information (<specific identifying information in file to be removed>), ensure that the data shared are in accordance with participant consent, and re-upload a fully anonymized data set. Please note that spreadsheet columns with personal information must be removed and not hidden as all hidden columns will appear in the published file. | As noted above, supplementary file S1 Table, which actually described the characteristics of Kenyan project research team members but NOT RESEARCH PARTICIPANTS, has now been deleted as supporting information. |
|  | **Reviewer #1** |  |
| 1. | **Abstract:** |  |
|  | The role of formal-informal SWM collectors towards plastic waste collection is missing. This should be highlighted (in line 19) to establish a linkage and a stronger basis for the study. A more logical order will be: Who handles plastics? What challenges are they facing? | This has been addressed in line 19 of the Abstract section (Page 2) |
|  | How many participants participated in each focus group? | This has been included in the methods section of the abstract and can be found in lines 26 and 27 (i.e. 6-12 participants per FGD (n=87)) |
| 2. | **Introduction** |  |
|  | The authors have provided an extensive background to the study and supported this with adequate literature. However, the sections look disjointed and verbose. The introduction could be shortened and more direct. It is recommended that the sections are re-ordered to ensure a chronology of the study’s background. This chronology could be interspersed or adopted: What is the global SWM (as already established in Lines 39 to 49), who are the actors of SWM, what are their specific roles in SWM, what is their socio-economic motivation, what research gaps remain in their specific role vis-à-vis plastic waste recycling? | The introduction has been re-ordered for a better flow, with some content abridged to create a more concise background to the study. |
| 3. | **Materials and Methods** |  |
|  | **study sites**  How much plastic waste/recyclables is generated in Ghana? | This information has now been included under the study sites (page 4). |
|  | Line 123,…and processed waste. Introduce “and” | This has been introduced (line 128, page 5) |
|  | Authors may consider including a map of the study areas | We have inserted a new Figure 1 in our Materials and Methods section, showing maps of the two study areas, drawing on the CC BY license of geoboundaries. |
|  | The authors may consider including information on the number of registered members of the SWM associations, the number of waste management companies, the number of recycling companies, among others. | In our revised manuscript, we now include some additional statistics on numbers of recycling companies and example membership figures for a SWM association. |
| 5. | Results and Discussion |  |
|  | Line 290 to 29 Confirm if the requested amount is GHS30 or 300 | The requested amount is indeed 300 cedis. |
|  | The dollar equivalent should be highlighted in brackets. | The dollar equivalent has been added (page 14) and for two other quotations, we have similarly added dollar equivalent values for KSh amounts. |
|  | Line 301 to 302 Modify the sentence…but before we you get… (we and you appear together) | This has been modified under the results section (Pages 14-15) |
|  | Under Table 5, under Kisumu SWM groups line 2, intermediaries should be formatted in terms of font and font size | This table has been formatted accordingly |
|  | Maintain consistency in font size and type (sentence case, capitalisation) across all tables | All tables have been formatted to have consistency in font size and type as well as capitalization. |
|  | Line 458 to 462 How does this finding compare with other studies in the region or globally | We have edited the text to address this comment. We now note that other studies have reported theft of personal positions (in insecure neighbourhoods) and of waste collection equipment, but not the specific theft of valuable separated plastic waste. |
|  | Authors should examine the influence of security agencies on collectors, particularly regarding the collection of illegal monies, intimidation, and its effects on (de)motivation for waste collectors. | We now return to the theme of harassment by authorities in paragraph 4 of our revised Discussion, noting that this is a recurrent theme in literature on informal waste collectors. |
|  | Line 535 provide a space between references and “chains” | This change has been effected (page 25) |
|  | **Reviewer # 2** |  |
| 6. | First, I recommend using consistent precision across all tables. Currently, some columns have precision to one digit while others do not. | All tables have now been formatted accordingly, with all percentages given to 1 decimal place. |
| 7. | please double-check the initials from lines 182 to 187. I am not sure if these are the initials of the authors or not. If they are, please ensure consistency. | We have amended the text here to make it clear that author initials are listed at this point, checking formatting of initials for consistency. |
| 8. | Lastly, Table 5 has a different font style compared to all the other tables. Please standardize the font style for uniformity. | Table 5 has been formatted to have consistent font size and style with all the other tables. |
