# Supplementary material for: Challenges and opportunities for plastic versus mixed waste enterprises in Greater Accra and Kisumu: A qualitative study
Source: PLoS One. 2026 Jun 8;21(6):e0350670. doi: 10.1371/journal.pone.0350670 (PMC13245779; doi:10.1371/journal.pone.0350670)
Supplement: Attachment — Submitted filename: PlosOne_Response_Reviewers22Apr2026.docx [file pone.0350670.s005.docx]

22^nd^ April 2026

Alison Parker

Academic Editor

PLOS One

Dear Dr. Parker,

**RESPONSE TO REVIEWERS’ COMMENTS: Challenges and opportunities for plastic versus mixed waste enterprises in Greater Accra and Kisumu: a qualitative study (ref: PONE-D-25-23611)**

Thank you for the constructive feedback provided on our manuscript. Below are comments made by reviewers as well as point-by-point responses indicating how and where these have been addressed throughout the manuscript.

| **S/N** | **REVIEW COMMENT** | **RESPONSES** |
| --- | --- | --- |
|  | **Reviewers’ comments** |  |
|  | **Reviewer #1** |  |
| 1. | Line 21 and 22 “The informal sector often fills gaps in plastic waste collection and recovery that are not met by formal SWM..” There are two full stops at the end. | This has been edited to only one full stop. This edit can be found on page 2, line 22. |
| 2. | Line 65-66 Mismanaged solid waste, including plastics, therefore accumulates in the environment, especially in off-grid areas.. There are two full stops at the end | This has been corrected to only one full stop. This edit can be found on page 3, lines 67-68. |
| 3. | Table 5 Under all the headings, particularly in the rows, some of the writings have been left in sentence cases, while some are completely left in lowercase. There should be consistency in the writing; For example, “Competition from unregulated mixed waste collectors” versus “occupational health risks.” The first instance starts with a capital “C” while the second starts with a lower case “O”. This inconsistency runs throughout the table on pages 23 and 24. | This correction has been made under table 5. The first words begin with upper cases throughout the rows. These changes can be found on pages 24-25. |
|  | **Reviewer # 3 (transposed from annotated pdf)** |  |
| 4. | Edit! double periods!  (Page 2, line 22) | This has been edited to only one full stop. This edit can be found on page 2, line 22. |
| 5. | Since the aim of the study was looking at comparison of the plastic and general waste collectors - perhaps the FGDs should be explicit as well as how many were conducted with plastic and general and again it would be good to know how many were conducted in Ghana and Kenya! ((Page 2, lines 28-30)) | The number of FGDs conducted within the various waste collector sub-groups for each country has now been included in the methods section of the abstract (lines 28-31; Page 2) |
| 6. | Edit! (line 66) | This has been edited to only one full stop. This edit can be found on page 3, lines 67-68. |
| 7. | I think this should be amplified as in some cases because informal waste pickers are ignored in the designing of the programs - in some cases they are not even allowed to dump the waste collected in designated landfills established by governments, municipal councils - and this results in indiscriminate disposal of such waste! so while the intention of the waste pickers is good - they end up worsening the situation!  Lines 76-77. | This has been addressed and more emphasis has been included regarding the contributions and challenges that informal waste collectors encounter. We now highlight that lack of formal recognition often affects their work during waste collection, transportation and disposal. These revisions can be found on pages 3 and 4, lines 71-73 and lines 82-85. |
| 8. | This number should be highlighted in Abstract! at least for the reader to understand proportion of the FGDs in each city!  Line 207 | The specific number of FGDs conducted within the various waste collector sub-groups for each country has been included in the methods section of the abstract (lines 28-31; Page 2) |
| 9. | How was this done? after initial analysis? sent a summary to participants?  (line 240) | The detailed process has now been included, and can be found on page 11, lines 248-251. |

Please note that we have also reviewed our three figures, using the NAAS tool to meet journal requirements as appropriate.

Thank you again for your kind review.

Yours Sincerely,

Prof. Jim Wright and Ruby Hornuvo (on behalf of co-authors)
