# Supplementary material for: Challenges and opportunities for plastic versus mixed waste enterprises in Greater Accra and Kisumu: A qualitative study
Source: PLoS One. 2026 Jun 8;21(6):e0350670. doi: 10.1371/journal.pone.0350670 (PMC13245779; doi:10.1371/journal.pone.0350670)
Supplement: Peer review history

# Peer review: Decision Letter 0

**DOI:** 10.1371/journal.pone.0350670.r001

**Authors:** Dereje Donacho

24 Jul 2025

PONE-D-25-23611

Challenges and opportunities for plastic versus mixed waste enterprises in Greater Accra and Kisumu: a qualitative study

PLOS ONE

Dear Dr. Wright,

Thank you for submitting your manuscript to PLOS ONE. After careful consideration, we feel that it has merit but does not fully meet PLOS ONE’s publication criteria as it currently stands. Therefore, we invite you to submit a revised version of the manuscript that addresses the points raised during the review process.

Please submit your revised manuscript by Sep 07 2025 11:59PM. If you will need more time than this to complete your revisions, please reply to this message or contact the journal office at plosone@plos.org. When you're ready to submit your revision, log on to https://www.editorialmanager.com/pone/ and select the 'Submissions Needing Revision' folder to locate your manuscript file.

Please include the following items when submitting your revised manuscript:

- A rebuttal letter that responds to each point raised by the academic editor and reviewer(s). You should upload this letter as a separate file labeled 'Response to Reviewers'.
- A marked-up copy of your manuscript that highlights changes made to the original version. You should upload this as a separate file labeled 'Revised Manuscript with Track Changes'.
- An unmarked version of your revised paper without tracked changes. You should upload this as a separate file labeled 'Manuscript'.

If you would like to make changes to your financial disclosure, please include your updated statement in your cover letter. Guidelines for resubmitting your figure files are available below the reviewer comments at the end of this letter.

If applicable, we recommend that you deposit your laboratory protocols in protocols.io to enhance the reproducibility of your results. Protocols.io assigns your protocol its own identifier (DOI) so that it can be cited independently in the future. For instructions see: https://journals.plos.org/plosone/s/submission-guidelines#loc-laboratory-protocols. Additionally, PLOS ONE offers an option for publishing peer-reviewed Lab Protocol articles, which describe protocols hosted on protocols.io. Read more information on sharing protocols at https://plos.org/protocols?utm_medium=editorial-email&utm_source=authorletters&utm_campaign=protocols.

We look forward to receiving your revised manuscript.

Kind regards,

Dereje Oljira Donacho, PhD

Academic Editor

PLOS ONE

Journal Requirements:

When submitting your revision, we need you to address these additional requirements.

1. Please ensure that your manuscript meets PLOS ONE's style requirements, including those for file naming. The PLOS ONE style templates can be found at

https://journals.plos.org/plosone/s/file?id=wjVg/PLOSOne_formatting_sample_main_body.pdf and https://journals.plos.org/plosone/s/file?id=ba62/PLOSOne_formatting_sample_title_authors_affiliations.pdf

2. Please include a complete copy of PLOS’ questionnaire on inclusivity in global research in your revised manuscript. Our policy for research in this area aims to improve transparency in the reporting of research performed outside of researchers’ own country or community. The policy applies to researchers who have travelled to a different country to conduct research, research with Indigenous populations or their lands, and research on cultural artefacts. The questionnaire can also be requested at the journal’s discretion for any other submissions, even if these conditions are not met.  Please find more information on the policy and a link to download a blank copy of the questionnaire here: https://journals.plos.org/plosone/s/best-practices-in-research-reporting. Please upload a completed version of your questionnaire as Supporting Information when you resubmit your manuscript.

3. Thank you for stating in your Funding Statement:

[The work was undertaken through the ‘Expanding safe water and waste management service access to off-grid populations in Africa’ project, funded through UKRI Collective award via the Global Challenges Research Fund (ref: ES/T008121/1). The support of the UK Economic and Social Research Council (ESRC) is gratefully acknowledged.].

Please provide an amended statement that declares *all* the funding or sources of support (whether external or internal to your organization) received during this study, as detailed online in our guide for authors at http://journals.plos.org/plosone/s/submit-now. Please also include the statement “There was no additional external funding received for this study.” in your updated Funding Statement.

Please include your amended Funding Statement within your cover letter. We will change the online submission form on your behalf.

4. Thank you for stating the following in the Competing Interests section:

[MD is academic representative on Ghana’s National Technical Working Group on Sanitation, which informs solid waste management policy. The other authors declare no competing interests.].

Please confirm that this does not alter your adherence to all PLOS ONE policies on sharing data and materials, by including the following statement: "This does not alter our adherence to  PLOS ONE policies on sharing data and materials.” (as detailed online in our guide for authors http://journals.plos.org/plosone/s/competing-interests).  If there are restrictions on sharing of data and/or materials, please state these. Please note that we cannot proceed with consideration of your article until this information has been declared.

Please include your updated Competing Interests statement in your cover letter; we will change the online submission form on your behalf.

5. We note that your paper includes detailed descriptions of individual participants. As per the PLOS ONE policy (http://journals.plos.org/plosone/s/submission-guidelines#loc-human-subjects-research) on papers that include identifying, or potentially identifying, information, the individual(s) or parent(s)/guardian(s) must be informed of the terms of the PLOS open-access (CC-BY) license and provide specific permission for publication of these details under the terms of this license. Please download the Consent Form for Publication in a PLOS Journal (http://journals.plos.org/plosone/s/file?id=8ce6/plos-consent-form-english.pdf). The signed consent form should not be submitted with the manuscript, but should be securely filed in the individual's case notes. Please amend the methods section and ethics statement of the manuscript to explicitly state that the patient/participant has provided consent for publication: “The individual in this manuscript has given written informed consent (as outlined in PLOS consent form) to publish these case details

6. We note that there is identifying data in the Supporting Information file <S1 Table>. Due to the inclusion of these potentially identifying data, we have removed this file from your file inventory. Prior to sharing human research participant data, authors should consult with an ethics committee to ensure data are shared in accordance with participant consent and all applicable local laws.

Data sharing should never compromise participant privacy. It is therefore not appropriate to publicly share personally identifiable data on human research participants. The following are examples of data that should not be shared:

-Name, initials, physical address

-Ages more specific than whole numbers

-Internet protocol (IP) address

-Specific dates (birth dates, death dates, examination dates, etc.)

-Contact information such as phone number or email address

-Location data

-ID numbers that seem specific (long numbers, include initials, titled “Hospital ID”) rather than random (small numbers in numerical order

Data that are not directly identifying may also be inappropriate to share, as in combination they can become identifying. For example, data collected from a small group of participants, vulnerable populations, or private groups should not be shared if they involve indirect identifiers (such as sex, ethnicity, location, etc.) that may risk the identification of study participants.

Additional guidance on preparing raw data for publication can be found in our Data Policy (https://journals.plos.org/plosone/s/data-availability#loc-human-research-participant-data-and-other-sensitive-data) and in the following article: http://www.bmj.com/content/340/bmj.c181.long.

Please remove or anonymize all personal information (<specific identifying information in file to be removed>), ensure that the data shared are in accordance with participant consent, and re-upload a fully anonymized data set. Please note that spreadsheet columns with personal information must be removed and not hidden as all hidden columns will appear in the published file.

If the reviewer comments include a recommendation to cite specific previously published works, please review and evaluate these publications to determine whether they are relevant and should be cited. There is no requirement to cite these works unless the editor has indicated otherwise.

[Note: HTML markup is below. Please do not edit.]

Reviewers' comments:

Reviewer's Responses to Questions

**Comments to the Author**

1. Is the manuscript technically sound, and do the data support the conclusions?

The manuscript must describe a technically sound piece of scientific research with data that supports the conclusions. Experiments must have been conducted rigorously, with appropriate controls, replication, and sample sizes. The conclusions must be drawn appropriately based on the data presented.

Reviewer #1: Yes

Reviewer #2: Yes

**********

2. Has the statistical analysis been performed appropriately and rigorously?

Reviewer #1: N/A

Reviewer #2: N/A

**********

3. Have the authors made all data underlying the findings in their manuscript fully available?

The PLOS Data policy requires authors to make all data underlying the findings described in their manuscript fully available without restriction, with rare exception (please refer to the Data Availability Statement in the manuscript PDF file). The data should be provided as part of the manuscript or its supporting information, or deposited to a public repository. For example, in addition to summary statistics, the data points behind means, medians and variance measures should be available. If there are restrictions on publicly sharing data—e.g. participant privacy or use of data from a third party—those must be specified.

Reviewer #1: Yes

Reviewer #2: Yes

**********

4. Is the manuscript presented in an intelligible fashion and written in standard English?

PLOS ONE does not copyedit accepted manuscripts, so the language in submitted articles must be clear, correct, and unambiguous. Any typographical or grammatical errors should be corrected at revision, so please note any specific errors here.

Reviewer #1: Yes

Reviewer #2: Yes

**********

5. Review Comments to the Author

Please use the space provided to explain your answers to the questions above. You may also include additional comments for the author, including concerns about dual publication, research ethics, or publication ethics. (Please upload your review as an attachment if it exceeds 20,000 characters)

Reviewer #1: Reviewers comment

The article examines the "Challenges and opportunities for plastic versus mixed waste enterprises in Greater Accra and Kisumu: a qualitative study," focusing on challenges faced by key waste collection groups. The foundations of the study are enshrined in the Sustainable Development Goals and the AU Agenda 2063. Its context is relevant for Africa, establishing a stronger basis for improving plastic/waste recycling in the region.

The manuscript demonstrates a robust methodological framework, engages in nuanced discussions, and offers valuable insights pertinent to both academic circles and industry professionals, among other stakeholders.

Specific comments:

1.The section on abstract:

The abstract presents a concise write-up about the topic. The background, objective and results of the study are summarised well. Although this seems to be sufficient, the following comments are suggested.

•The role of formal-informal SWM collectors towards plastic waste collection is missing. This should be highlighted (in line 19) to establish a linkage and a stronger basis for the study. A more logical order will be: Who handles plastics? What challenges are they facing?

•How many participants participated in each focus group?

2.The section on introduction:

The authors have provided an extensive background to the study and supported this with adequate literature. However, the sections look disjointed and verbose. The introduction could be shortened and more direct. It is recommended that the sections are re-ordered to ensure a chronology of the study’s background. This chronology could be interspersed or adopted: What is the global SWM (as already established in Lines 39 to 49), who are the actors of SWM, what are their specific roles in SWM, what is their socio-economic motivation, what research gaps remain in their specific role vis-à-vis plastic waste recycling?

3.The section on study sites:

•How much plastic waste/recyclables is generated in Ghana

•Line 123,…and processed waste. Introduce “and”

•Authors may consider including a map of the study areas

•The authors may consider including information on the number of registered members of the SWM associations, the number of waste management companies, the number of recycling companies, among others.

4.The section on methodology: The methodology discussed is robust and reproducible. This gives credence to the data collected.

5.The section on Results and Discussion:

The results and discussions are well presented. The following modifications should be considered

•Line 290 to 29 Confirm if the requested amount is GHS30 or 300

•The dollar equivalent should be highlighted in brackets.

•Line 301 to 302 Modify the sentence…but before we you get… (we and you appear together)

•Under Table 5, under Kisumu SWM groups line 2, intermediaries should be formatted in terms of font and font size

•Maintain consistency in font size and type (sentence case, capitalisation) across all tables

•Line 458 to 462 How does this finding compare with other studies in the region or globally

•Authors should examine the influence of security agencies on collectors, particularly regarding the collection of illegal monies, intimidation, and its effects on (de)motivation for waste collectors.

•Line 535 provide a space between references and “chains”

6.The section on Conclusion: The conclusions and recommendations are sufficient.

Reviewer #2: I enjoyed reading the manuscript by Hornuvo et al., which describes the challenges and opportunities associated with plastic and mixed waste enterprise in the Greater Accra and Kisumu areas. I must admit that it is not the usual manuscript style I encounter, but I find it informative and helpful nonetheless.

I don't have many comments for the authors. As mentioned above, the manuscript style is somewhat different, but I don't think it diminishes its value. I just have a couple of small suggestions. First, I recommend using consistent precision across all tables. Currently, some columns have precision to one digit while others do not.

Additionally, please double-check the initials from lines 182 to 187. I am not sure if these are the initials of the authors or not. If they are, please ensure consistency.

Lastly, Table 5 has a different font style compared to all the other tables. Please standardize the font style for uniformity.

**********

6. PLOS authors have the option to publish the peer review history of their article (what does this mean?). If published, this will include your full peer review and any attached files.

If you choose “no”, your identity will remain anonymous but your review may still be made public.

**Do you want your identity to be public for this peer review?** For information about this choice, including consent withdrawal, please see our Privacy Policy.

Reviewer #1: No

Reviewer #2: No

**********

[NOTE: If reviewer comments were submitted as an attachment file, they will be attached to this email and accessible via the submission site. Please log into your account, locate the manuscript record, and check for the action link "View Attachments". If this link does not appear, there are no attachment files.]

While revising your submission, please upload your figure files to the Preflight Analysis and Conversion Engine (PACE) digital diagnostic tool, https://pacev2.apexcovantage.com/. PACE helps ensure that figures meet PLOS requirements. To use PACE, you must first register as a user. Registration is free. Then, login and navigate to the UPLOAD tab, where you will find detailed instructions on how to use the tool. If you encounter any issues or have any questions when using PACE, please email PLOS at figures@plos.org. Please note that Supporting Information files do not need this step.

Attachment Submitted filename: Comments.pdf

# Author comment: Author response to Decision Letter 1

**DOI:** 10.1371/journal.pone.0350670.r002

20 Aug 2025

Thank you for the constructive feedback provided on our manuscript. Below are comments made by reviewers and the editorial team, as well as point-by-point responses indicating how and where these have been addressed throughout the manuscript.

Editorial Comments

============

COMMENT: Please ensure that your manuscript meets PLOS ONE's style requirements, including those for file naming. The PLOS ONE style templates can be found at…

RESPONSE: The manuscript has been formatted in accordance with PLOS ONE’s manuscript style requirements. Please note that on reviewing PLOS ONE guidance on multi-part figures, we have separated the multipart Figure 1 in our original manuscript into two separate figures, now numbered Figure 2 and 3 (with the introduction of a new additional Figure 1 in response to Reviewer 1's comments).

COMMENT: Please include a complete copy of PLOS’ questionnaire on inclusivity in global research in your revised manuscript. Our policy for research in this area aims to improve transparency in the reporting of research performed outside of researchers’ own country or community. The policy applies to researchers who have travelled to a different country to conduct research, research with Indigenous populations or their lands, and research on cultural artefacts. The questionnaire can also be requested at the journal’s discretion for any other submissions, even if these conditions are not met. Please find more information on the policy and a link to download a blank copy of the questionnaire here: https://journals.plos.org/plosone/s/best-practices-in-research-reporting. Please upload a completed version of your questionnaire as Supporting Information when you resubmit your manuscript.

RESPONSE: We have completed the questionnaire on inclusivity in global research and uploaded it as Supporting Information as requested.

COMMENT: Thank you for stating in your Funding Statement:

[The work was undertaken through the ‘Expanding safe water and waste management service access to off-grid populations in Africa’ project, funded through UKRI Collective award via the Global Challenges Research Fund (ref: ES/T008121/1). The support of the UK Economic and Social Research Council (ESRC) is gratefully acknowledged.].

Please provide an amended statement that declares *all* the funding or sources of support (whether external or internal to your organization) received during this study, as detailed online in our guide for authors at http://journals.plos.org/plosone/s/submit-now. Please also include the statement “There was no additional external funding received for this study.” in your updated Funding Statement.

Please include your amended Funding Statement within your cover letter. We will change the online submission form on your behalf.

RESPONSE: As requested, we provide an updated funding including the explicit statement about no further external funding via our latest covering letter.

COMMENT: Thank you for stating the following in the Competing Interests section:

[MD is academic representative on Ghana’s National Technical Working Group on Sanitation, which informs solid waste management policy. The other authors declare no competing interests.].

Please confirm that this does not alter your adherence to all PLOS ONE policies on sharing data and materials, by including the following statement: "This does not alter our adherence to PLOS ONE policies on sharing data and materials.” (as detailed online in our guide for authors http://journals.plos.org/plosone/s/competing-interests). If there are restrictions on sharing of data and/or materials, please state these. Please note that we cannot proceed with consideration of your article until this information has been declared.

Please include your updated Competing Interests statement in your cover letter; we will change the online submission form on your behalf.

RESPONSE: The updated competing interest has been included in the cover letter.

COMMENT: We note that your paper includes detailed descriptions of individual participants. As per the PLOS ONE policy (http://journals.plos.org/plosone/s/submission-guidelines#loc-human-subjects-research) on papers that include identifying, or potentially identifying, information, the individual(s) or parent(s)/guardian(s) must be informed of the terms of the PLOS open-access (CC-BY) license and provide specific permission for publication of these details under the terms of this license. Please download the Consent Form for Publication in a PLOS Journal (http://journals.plos.org/plosone/s/file?id=8ce6/plos-consent-form-english.pdf). The signed consent form should not be submitted with the manuscript, but should be securely filed in the individual's case notes. Please amend the methods section and ethics statement of the manuscript to explicitly state that the patient/participant has provided consent for publication: “The individual in this manuscript has given written informed consent (as outlined in PLOS consent form) to publish these case details”.

RESPONSE: On reviewing our manuscript, we believe that this comment refers particularly to the supplementary materials (S1 Table) of our original submission, as per the comment below. This table actually described the demographic and educational characteristics of the Kenyan research team members, NOT OUR PARTICIPANTS. We included this table to provide detail on the backgrounds and nature of those conducting this qualitative research study, thereby meeting items on the COREQ checklist for reporting qualitative research (https://www.equator-network.org/reporting-guidelines/coreq/). Nonetheless, we accept that there is a data protection issue around reporting the personal characteristics of Kenyan project team members, so we have now deleted it without compromising COREQ compliance. We have also reviewed all quotations in the main manuscript, further redacting any personal details (e.g. of specific sites or occupations) that could potentially lead to inadvertent disclosure of participant identities. If we have misunderstood the editorial concerns, please let us know.

COMMENT: We note that there is identifying data in the Supporting Information file <S1 Table>. Due to the inclusion of these potentially identifying data, we have removed this file from your file inventory. Prior to sharing human research participant data, authors should consult with an ethics committee to ensure data are shared in accordance with participant consent and all applicable local laws.

Data sharing should never compromise participant privacy. It is therefore not appropriate to publicly share personally identifiable data on human research participants. The following are examples of data that should not be shared:

-Name, initials, physical address

-Ages more specific than whole numbers

-Internet protocol (IP) address

-Specific dates (birth dates, death dates, examination dates, etc.)

-Contact information such as phone number or email address

-Location data

-ID numbers that seem specific (long numbers, include initials, titled “Hospital ID”) rather than random (small numbers in numerical order

Data that are not directly identifying may also be inappropriate to share, as in combination they can become identifying. For example, data collected from a small group of participants, vulnerable populations, or private groups should not be shared if they involve indirect identifiers (such as sex, ethnicity, location, etc.) that may risk the identification of study participants.

Additional guidance on preparing raw data for publication can be found in our Data Policy (https://journals.plos.org/plosone/s/data-availability#loc-human-research-participant-data-and-other-sensitive-data) and in the following article: http://www.bmj.com/content/340/bmj.c181.long.

Please remove or anonymize all personal information (<specific identifying information in file to be removed>), ensure that the data shared are in accordance with participant consent, and re-upload a fully anonymized data set. Please note that spreadsheet columns with personal information must be removed and not hidden as all hidden columns will appear in the published file.

RESPONSE: As noted above, supplementary file S1 Table, which actually described the characteristics of Kenyan project research team members but NOT RESEARCH PARTICIPANTS, has now been deleted as supporting information.

Reviewer #1

========

1. Abstract:

• The role of formal-informal SWM collectors towards plastic waste collection is missing. This should be highlighted (in line 19) to establish a linkage and a stronger basis for the study. A more logical order will be: Who handles plastics? What challenges are they facing?

RESPONSE: This has been addressed in line 19 of the Abstract section (Page 2)

• How many participants participated in each focus group?

RESPONSE: This has been included in the methods section of the abstract and can be found in lines 26 and 27 (i.e. 6-12 participants per FGD (n=87))

2. Introduction

• The authors have provided an extensive background to the study and supported this with adequate literature. However, the sections look disjointed and verbose. The introduction could be shortened and more direct. It is recommended that the sections are re-ordered to ensure a chronology of the study’s background. This chronology could be interspersed or adopted: What is the global SWM (as already established in Lines 39 to 49), who are the actors of SWM, what are their specific roles in SWM, what is their socio-economic motivation, what research gaps remain in their specific role vis-à-vis plastic waste recycling?

RESPONSE: The introduction has been re-ordered for a better flow, with some content abridged to create a more concise background to the study.

3. Materials and Methods

• study sites

How much plastic waste/recyclables is generated in Ghana?

RESPONSE: This information has now been included under the study sites (page 4).

• Line 123,…and processed waste. Introduce “and”

RESPONSE: This has been introduced (line 128, page 5)

• Authors may consider including a map of the study areas

RESPONSE: We have inserted a new Figure 1 in our Materials and Methods section, showing maps of the two study areas, drawing on the CC BY license of geoboundaries.

• The authors may consider including information on the number of registered members of the SWM associations, the number of waste management companies, the number of recycling companies, among others.

RESPONSE: In our revised manuscript, we now include some additional statistics on numbers of recycling companies and example membership figures for a SWM association.

5. Results and Discussion

• Line 290 to 29 Confirm if the requested amount is GHS30 or 300

RESPONSE: The requested amount is indeed 300 cedis.

• The dollar equivalent should be highlighted in brackets.

RESPONSE: The dollar equivalent has been added (page 14) and for two other quotations, we have similarly added dollar equivalent values for KSh amounts.

• Line 301 to 302 Modify the sentence…but before we you get… (we and you appear together)

RESPONSE: This has been modified under the results section (Pages 14-15)

• Under Table 5, under Kisumu SWM groups line 2, intermediaries should be formatted in terms of font and font size RESPONSE: This table has been formatted accordingly

Maintain consistency in font size and type (sentence case, capitalisation) across all tables

RESPONSE: All tables have been formatted to have consistency in font size and type as well as capitalization.

• Line 458 to 462 How does this finding compare with other studies in the region or globally

RESPONSE: We have edited the text to address this comment.

RESPONSE: We now note that other studies have reported theft of personal positions (in insecure neighbourhoods) and of waste collection equipment, but not the specific theft of valuable separated plastic waste.

• Authors should examine the influence of security agencies on collectors, particularly regarding the collection of illegal monies, intimidation, and its effects on (de)motivation for waste collectors.

RESPONSE: We now return to the theme of harassment by authorities in paragraph 4 of our revised Discussion, noting that this is a recurrent theme in literature on informal waste collectors.

• Line 535 provide a space between references and “chains”

RESPONSE: This change has been effected (page 25)

Reviewer # 2

========

6. First, I recommend using consistent precision across all tables. Currently, some columns have precision to one digit while others do not.

RESPONSE: All tables have now been formatted accordingly, with all percentages given to 1 decimal place.

7. please double-check the initials from lines 182 to 187. I am not sure if these are the initials of the authors or not. If they are, please ensure consistency.

RESPONSE: We have amended the text here to make it clear that author initials are listed at this point, checking formatting of initials for consistency.

8. Lastly, Table 5 has a different font style compared to all the other tables. Please standardize the font style for uniformity.

RESPONSE: Table 5 has been formatted to have consistent font size and style with all the other tables.

Attachment Submitted filename: ResponseReviewers_August2025.docx

# Peer review: Decision Letter 1

**DOI:** 10.1371/journal.pone.0350670.r003

**Authors:** Alison Parker

18 Mar 2026

PONE-D-25-23611R1

Challenges and opportunities for plastic versus mixed waste enterprises in Greater Accra and Kisumu: a qualitative study

PLOS One

Dear Dr. Wright,

Thank you for submitting your manuscript to PLOS ONE. After careful consideration, we feel that it has merit but does not fully meet PLOS ONE’s publication criteria as it currently stands. Therefore, we invite you to submit a revised version of the manuscript that addresses the points raised during the review process.

Reviewer 1 has some additional minor comments and I also secured a further reviewer to ensure a full rigorous review, and they have some minor comments too.

Please submit your revised manuscript by May 02 2026 11:59PM. If you will need more time than this to complete your revisions, please reply to this message or contact the journal office at plosone@plos.org. When you're ready to submit your revision, log on to https://www.editorialmanager.com/pone/ and select the 'Submissions Needing Revision' folder to locate your manuscript file.

Please include the following items when submitting your revised manuscript:

- A letter that responds to each point raised by the academic editor and reviewer(s). You should upload this letter as a separate file labeled 'Response to Reviewers'.
- A marked-up copy of your manuscript that highlights changes made to the original version. You should upload this as a separate file labeled 'Revised Manuscript with Track Changes'.
- An unmarked version of your revised paper without tracked changes. You should upload this as a separate file labeled 'Manuscript'.

-->If you would like to make changes to your financial disclosure, please include your updated statement in your cover letter. Guidelines for resubmitting your figure files are available below the reviewer comments at the end of this letter.

If applicable, we recommend that you deposit your laboratory protocols in protocols.io to enhance the reproducibility of your results. Protocols.io assigns your protocol its own identifier (DOI) so that it can be cited independently in the future. For instructions see: https://journals.plos.org/plosone/s/submission-guidelines#loc-laboratory-protocols. Additionally, PLOS ONE offers an option for publishing peer-reviewed Lab Protocol articles, which describe protocols hosted on protocols.io. Read more information on sharing protocols at https://plos.org/protocols?utm_medium=editorial-email&utm_source=authorletters&utm_campaign=protocols.

We look forward to receiving your revised manuscript.

Kind regards,

Alison Parker

Academic Editor

PLOS One

Journal Requirements:

1. If the reviewer comments include a recommendation to cite specific previously published works, please review and evaluate these publications to determine whether they are relevant and should be cited. There is no requirement to cite these works unless the editor has indicated otherwise.

2. Please review your reference list to ensure that it is complete and correct. If you have cited papers that have been retracted, please include the rationale for doing so in the manuscript text, or remove these references and replace them with relevant current references. Any changes to the reference list should be mentioned in the rebuttal letter that accompanies your revised manuscript. If you need to cite a retracted article, indicate the article’s retracted status in the References list and also include a citation and full reference for the retraction notice.

[Note: HTML markup is below. Please do not edit.]

Reviewers' comments:

Reviewer's Responses to Questions

**Comments to the Author**

1. If the authors have adequately addressed your comments raised in a previous round of review and you feel that this manuscript is now acceptable for publication, you may indicate that here to bypass the “Comments to the Author” section, enter your conflict of interest statement in the “Confidential to Editor” section, and submit your "Accept" recommendation.

Reviewer #1: All comments have been addressed

Reviewer #2: All comments have been addressed

Reviewer #3: All comments have been addressed

**********

2. Is the manuscript technically sound, and do the data support the conclusions?

The manuscript must describe a technically sound piece of scientific research with data that supports the conclusions. Experiments must have been conducted rigorously, with appropriate controls, replication, and sample sizes. The conclusions must be drawn appropriately based on the data presented.

Reviewer #1: Yes

Reviewer #2: Yes

Reviewer #3: Yes

**********

3. Has the statistical analysis been performed appropriately and rigorously?

Reviewer #1: Yes

Reviewer #2: N/A

Reviewer #3: N/A

**********

4. Have the authors made all data underlying the findings in their manuscript fully available?

The PLOS Data policy requires authors to make all data underlying the findings described in their manuscript fully available without restriction, with rare exception (please refer to the Data Availability Statement in the manuscript PDF file). The data should be provided as part of the manuscript or its supporting information, or deposited to a public repository. For example, in addition to summary statistics, the data points behind means, medians and variance measures should be available. If there are restrictions on publicly sharing data—e.g. participant privacy or use of data from a third party—those must be specified.

Reviewer #1: Yes

Reviewer #2: Yes

Reviewer #3: Yes

**********

5. Is the manuscript presented in an intelligible fashion and written in standard English?

PLOS ONE does not copyedit accepted manuscripts, so the language in submitted articles must be clear, correct, and unambiguous. Any typographical or grammatical errors should be corrected at revision, so please note any specific errors here.

Reviewer #1: Yes

Reviewer #2: Yes

Reviewer #3: Yes

**********

6. Review Comments to the Author

Please use the space provided to explain your answers to the questions above. You may also include additional comments for the author, including concerns about dual publication, research ethics, or publication ethics. (Please upload your review as an attachment if it exceeds 20,000 characters)

Reviewer #1: Line 21 and 22 “The informal sector often fills gaps in plastic waste collection and recovery that are not met by formal SWM..”

There are two full stops at the end.

Line 65-66 Mismanaged solid waste, including plastics, therefore accumulates in the

environment, especially in off-grid areas..

There are two full stops at the end.

Table 5 Under all the headings, particularly in the rows, some of the writings have been left in sentence cases, while some are completely left in lowercase. There should be consistency in the writing; For example, “Competition from unregulated mixed waste collectors” versus “occupational health risks.” The first instance starts with a capital “C” while the second starts with a lower case “O”. This inconsistency runs throughout the table on pages 23 and 24.

Reviewer #2: The authors have adequately addressed my comments. I do not have any additional comments for the authors.

Reviewer #3: Comments from the first submission have been revised and updated appropriately in the current manuscript. I, have however added few comments in track changes.

**********

7. PLOS authors have the option to publish the peer review history of their article (what does this mean?). If published, this will include your full peer review and any attached files.

If you choose “no”, your identity will remain anonymous but your review may still be made public.

**Do you want your identity to be public for this peer review?** For information about this choice, including consent withdrawal, please see our Privacy Policy.

Reviewer #1: No

Reviewer #2: No

Reviewer #3: **Yes:** Brighton Austin Chunga

**********

[NOTE: If reviewer comments were submitted as an attachment file, they will be attached to this email and accessible via the submission site. Please log into your account, locate the manuscript record, and check for the action link "View Attachments". If this link does not appear, there are no attachment files.]

To ensure your figures meet our technical requirements, please review our figure guidelines: https://journals.plos.org/plosone/s/figures

You may also use PLOS’s free figure tool, NAAS, to help you prepare publication quality figures: https://journals.plos.org/plosone/s/figures#loc-tools-for-figure-preparation.

NAAS will assess whether your figures meet our technical requirements by comparing each figure against our figure specifications.

Attachment Submitted filename: PONE-D-25-23611_R1_Comments.pdf

# Author comment: Author response to Decision Letter 2

**DOI:** 10.1371/journal.pone.0350670.r004

14 May 2026

Response to Reviewers’ comments

Reviewer #1

1. Line 21 and 22 “The informal sector often fills gaps in plastic waste collection and recovery that are not met by formal SWM..”

There are two full stops at the end.

RESPONSE: This has been edited to only one full stop. This edit can be found on page 2, line 22.

2. Line 65-66 Mismanaged solid waste, including plastics, therefore accumulates in the

environment, especially in off-grid areas..

There are two full stops at the end

RESPONSE: This has been corrected to only one full stop. This edit can be found on page 3, lines 67-68.

3. Table 5 Under all the headings, particularly in the rows, some of the writings have been left in sentence cases, while some are completely left in lowercase. There should be consistency in the writing; For example, “Competition from unregulated mixed waste collectors” versus “occupational health risks.” The first instance starts with a capital “C” while the second starts with a lower case “O”. This inconsistency runs throughout the table on pages 23 and 24.

RESPONSE: This correction has been made under table 5. The first words begin with upper cases throughout the rows. These changes can be found on pages 24-25.

Reviewer #2 was satisfied with our previous version of this manuscript, so there are no comments from this reviewer to address.

Reviewer # 3 (transposed from annotated pdf)

4. Edit! double periods!

(Page 2, line 22)

RESPONSE: This has been edited to only one full stop. This edit can be found on page 2, line 22.

5. Since the aim of the study was looking at comparison of the plastic and general waste collectors - perhaps the FGDs should be explicit as well as how many were conducted with plastic and general and again it would be good to know how many were conducted in Ghana and Kenya! ((Page 2, lines 28-30))

RESPONSE: The number of FGDs conducted within the various waste collector sub-groups for each country has now been included in the methods section of the abstract (lines 28-31; Page 2)

6. Edit! (line 66)

RESPONSE: This has been edited to only one full stop. This edit can be found on page 3, lines 67-68.

7. I think this should be amplified as in some cases because informal waste pickers are ignored in the designing of the programs - in some cases they are not even allowed to dump the waste collected in designated landfills established by governments, municipal councils - and this results in indiscriminate disposal of such waste! so while the intention of the waste pickers is good - they end up worsening the situation!

Lines 76-77.

RESPONSE: This has been addressed and more emphasis has been included regarding the contributions and challenges that informal waste collectors encounter. We now highlight that lack of formal recognition often affects their work during waste collection, transportation and disposal. These revisions can be found on pages 3 and 4, lines 71-73 and lines 82-85.

8. This number should be highlighted in Abstract! at least for the reader to understand proportion of the FGDs in each city! Line 207

RESPONSE: The specific number of FGDs conducted within the various waste collector sub-groups for each country has been included in the methods section of the abstract (lines 28-31; Page 2)

9. How was this done? after initial analysis? sent a summary to participants?

(line 240)

RESPONSE: The detailed process has now been included, and can be found on page 11, lines 248-251.

RESPONSE TO EDITORIAL COMMENTS: Please note that we have also reviewed our three figures, using the NAAS tool to meet journal requirements as appropriate.

Attachment Submitted filename: PlosOne_Response_Reviewers22Apr2026.docx

# Editor report: Decision Letter 2

**DOI:** 10.1371/journal.pone.0350670.r005

**Authors:** Alison Parker

17 May 2026

Challenges and opportunities for plastic versus mixed waste enterprises in Greater Accra and Kisumu: a qualitative study

PONE-D-25-23611R2

Dear Dr. Wright,

We’re pleased to inform you that your manuscript has been judged scientifically suitable for publication and will be formally accepted for publication once it meets all outstanding technical requirements.

Within one week, you’ll receive an e-mail detailing the required amendments. When these have been addressed, you’ll receive a formal acceptance letter and your manuscript will be scheduled for publication.

An invoice will be generated when your article is formally accepted. Please note, if your institution has a publishing partnership with PLOS and your article meets the relevant criteria, all or part of your publication costs will be covered. Please make sure your user information is up-to-date by logging into Editorial Manager at Editorial Manager® and clicking the ‘Update My Information' link at the top of the page. For questions related to billing, please contact billing support.

If your institution or institutions have a press office, please notify them about your upcoming paper to help maximize its impact. If they’ll be preparing press materials, please inform our press team as soon as possible -- no later than 48 hours after receiving the formal acceptance. Your manuscript will remain under strict press embargo until 2 pm Eastern Time on the date of publication. For more information, please contact onepress@plos.org.

Kind regards,

Alison Parker

Academic Editor

PLOS One

Additional Editor Comments (optional):

Reviewers' comments:

# Editor report: Acceptance letter

**DOI:** 10.1371/journal.pone.0350670.r006

**Authors:** Alison Parker

PONE-D-25-23611R2

PLOS One

Dear Dr. Wright,

I'm pleased to inform you that your manuscript has been deemed suitable for publication in PLOS One. Congratulations! Your manuscript is now being handed over to our production team.

At this stage, our production department will prepare your paper for publication. This includes ensuring the following:

* All references, tables, and figures are properly cited

* All relevant supporting information is included in the manuscript submission,

* There are no issues that prevent the paper from being properly typeset

You will receive further instructions from the production team, including instructions on how to review your proof when it is ready. Please keep in mind that we are working through a large volume of accepted articles, so please give us a few days to review your paper and let you know the next and final steps.

Lastly, if your institution or institutions have a press office, please let them know about your upcoming paper now to help maximize its impact. If they'll be preparing press materials, please inform our press team within the next 48 hours. Your manuscript will remain under strict press embargo until 2 pm Eastern Time on the date of publication. For more information, please contact onepress@plos.org.

You will receive an invoice from PLOS for your publication fee after your manuscript has reached the completed accept phase. If you receive an email requesting payment before acceptance or for any other service, this may be a phishing scheme. Learn how to identify phishing emails and protect your accounts at https://explore.plos.org/phishing.

If we can help with anything else, please email us at customercare@plos.org.

Thank you for submitting your work to PLOS ONE and supporting open access.

Kind regards,

PLOS ONE Editorial Office Staff

on behalf of

Dr. Alison Parker

Academic Editor

PLOS One
